# Baseline gut microbiota and metabolome predict durable immunogenicity to SARS-CoV-2 vaccines

**DOI:** 10.1038/s41392-023-01629-8

**Published:** 2023-09-25

**Authors:** Ye Peng, Lin Zhang, Chris K. P. Mok, Jessica Y. L. Ching, Shilin Zhao, Matthew K. L. Wong, Jie Zhu, Chunke Chen, Shilan Wang, Shuai Yan, Biyan Qin, Yingzhi Liu, Xi Zhang, Chun Pun Cheung, Pui Kuan Cheong, Ka Long Ip, Adrian C. H. Fung, Kenneth K. Y. Wong, David S. C. Hui, Francis K. L. Chan, Siew C. Ng, Hein M. Tun

**Affiliations:** 1 Microbiota I-Center (MagIC), Hong Kong, China; 2 Jockey Club School of Public Health and Primary Care, The Chinese University of Hong Kong, Hong Kong, China; 3 Li Ka Shing Institute of Health Sciences, Faculty of Medicine, The Chinese University of Hong Kong, Hong Kong, China; 4 Department of Medicine and Therapeutics, The Chinese University of Hong Kong, Hong Kong, China; 5 Department of Surgery, LKS Faculty of Medicine, The University of Hong Kong, Hong Kong, China; 6 Stanley Ho Centre for Emerging Infectious Diseases, Faculty of Medicine, The Chinese University of Hong Kong, Hong Kong, China; 7 Centre for Gut Microbiota Research, The Chinese University of Hong Kong, Hong Kong, China

**Keywords:** Vaccines, Industrial microbiology

## Abstract

The role of gut microbiota in modulating the durability of COVID-19 vaccine immunity is yet to be characterised. In this cohort study, we collected blood and stool samples of 121 BNT162b2 and 40 CoronaVac vaccinees at baseline, 1 month, and 6 months post vaccination (p.v.). Neutralisation antibody, plasma cytokine and chemokines were measured and associated with the gut microbiota and metabolome composition. A significantly higher level of neutralising antibody (at 6 months p.v.) was found in BNT162b2 vaccinees who had higher relative abundances of *Bifidobacterium adolescentis*, *Bifidobacterium bifidum*, and *Roseburia faecis* as well as higher concentrations of nicotinic acid (Vitamin B) and γ-Aminobutyric acid (*P* < 0.05) at baseline. CoronaVac vaccinees with high neutralising antibodies at 6 months p.v. had an increased relative abundance of *Phocaeicola dorei*, a lower relative abundance of *Faecalibacterium prausnitzii*, and a higher concentration of L-tryptophan (*P* < 0.05) at baseline. A higher antibody level at 6 months p.v. was also associated with a higher relative abundance of *Dorea formicigenerans* at 1 month p.v. among CoronaVac vaccinees (Rho = 0.62, *p* = 0.001, FDR = 0.123). Of the species altered following vaccination, 79.4% and 42.0% in the CoronaVac and BNT162b2 groups, respectively, recovered at 6 months. Specific to CoronaVac vaccinees, both bacteriome and virome diversity depleted following vaccination and did not recover to baseline at 6 months p.v. (FDR < 0.1). In conclusion, this study identified potential microbiota-based adjuvants that may extend the durability of immune responses to SARS-CoV-2 vaccines.

## Introduction

Severe acute respiratory syndrome coronavirus 2 (SARS-CoV-2) vaccines have substantially reduced coronavirus disease 2019 (COVID-19) disease mortality and severe illness.^1–3^ However, immune responses to vaccination are variable and vaccine effectiveness wanes over time regardless of the number of doses or vaccine type,^4^ and boosters are often necessary.

Immune responses to COVID-19 vaccination are known to be affected by multiple factors including genetics,^5^ obesity,^6^ and an individual’s baseline gut microbiome composition.^7,8^ We previously reported an association between gut microbiome composition and short-term vaccine immunogenicity.^7^ The relative abundances of certain gut bacteria including *Roseburia faecis* and *Bifidobacterium adolescentis* were consistently higher in vaccine high responders and positively correlated with anti-SARS-CoV-2 antibody levels 1 month after a second dose of BNT162b2 and CoronaVac vaccination.^7^ However, assessments on long-term immunity are required to determine if and how gut microbiome composition affects COVID-19 vaccine protection durability.

Immunogenicity to COVID-19 vaccines was also shown to be correlated with the gut metabolome. In a cohort of 52 BNT162b2 vaccinees, Lunken et al. reported negative associations between levels of competitive binding antibodies to the receptor binding domain (RBD) at 12 weeks after the first vaccine dose and total gut concentrations of branched-chain fatty acids, isobutyric acid, and isovaleric acid at baseline.^8^ Among patients with inflammatory bowel diseases (IBDs) who received either the BNT162b2 or the ChAdOx1 nCoV-19 (Oxford/AstraZeneca) vaccine, those who had higher anti-SARS-CoV-2 antibody levels at 14–100 days after the second dose had higher concentrations of trimethylamine, isobutyrate, and omega-muricholic acid but lower concentrations of succinate, phenylalanine, taurolithocholate, and taurodeoxycholate in their gut at baseline.^9^ In addition, in a cohort of inactivated BBIBP-CorV vaccinees, anti-RBD antibody levels at 42 days p.v. were positively associated with faecal concentrations of isovaleric acid, butyric acid, and acetic acid at the same date (42 days p.v.), but not at baseline.^10^ However, the associations between baseline gut metabolome and immune responses to COVID-19 vaccines over a longer term have yet to be described.

COVID-19 vaccination can also change the composition of the gut microbiome. We found that 1 month after a second vaccine dose, the relative abundances of *Bacteroides caccae* (for both CoronaVac and BNT162b2) and *Alistipes shahii* (for BNT162b2 only) increased significantly.^7^ These findings have been corroborated by Lunken et al.^8^, who observed similar changes and reduced alpha diversity in the gut microbiome of a Canadian cohort following the first dose of BNT162b2. However, the ramifications of these changes have yet to be elucidated. Changes in microbiome composition and loss of diversity have been associated with the development of diabetes^11^ and IBD,^12^ among others.

We performed a prospective, longitudinal study in Hong Kong^7^ to further characterise the microbiota composition associated with the durability of vaccine-induced immunity and to determine the impact of vaccines on long-term gut microbiota changes and recovery. We analysed the gut microbiome, serum antibody levels, and immunological and metabolomics data over a period of 6 months to better elucidate the bi-directional interaction between gut microbiome composition and vaccination across two different vaccine types (CoronaVac vs. BNT162b2).

## Results

### Dynamics of immunogenicity at 6 months after BNT162b2 and CoronaVac vaccination

**Fig. 1:**
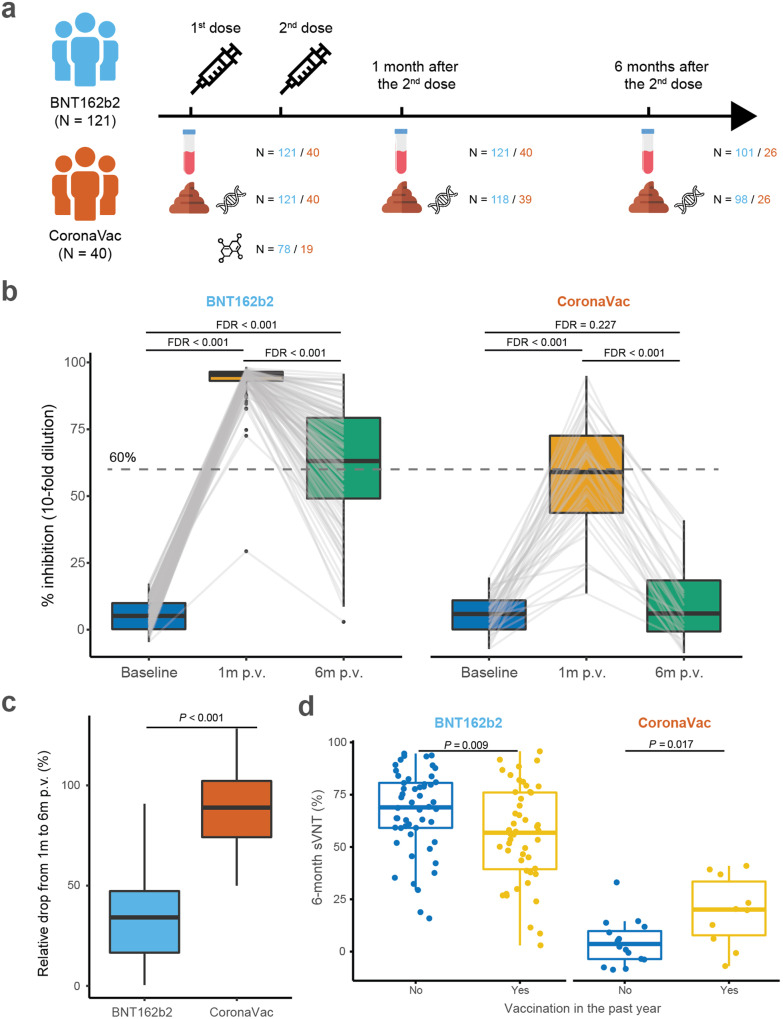
Study design and dynamics of immunity to SARS-CoV-2 from baseline to 6 months post-vaccination. **a** Study design. **b** sVNT levels from baseline to 6 months after the second vaccine dose (post vaccination, p.v.). *P* values were given by paired Wilcoxon’s rank-sum tests and were FDR-corrected. **c** Relative drop in sVNT level from 1 month to 6 months p.v. **d** sVNT levels at 6 months after the second vaccine dose by vaccination history. *P* value was given by Wilcoxon’s rank-sum test. Elements on boxplots: centre line, median; box limits, upper and lower quartiles; whiskers, 1.5 × IQR; points, outliers

From April 2021 to March 2022, we longitudinally followed 161 COVID-19 vaccinees who had received BNT162b2 (*N* = 121, 65.3% females) or CoronaVac vaccines (*N* = 40, 72.5% females). BNT162b2 vaccinees were younger compared to CoronaVac vaccinees [median (IQR) in years: 42 (29, 54) vs. 55 (39.75, 57), *P* = 0.005]; they also had less prevalence of hypertension [*N* (%): 7 (5.8) vs. 7 (17.5), *P* = 0.045]. These subjects had faecal samples and blood samples taken at baseline, and 1 and 6 months after the second vaccine dose (post vaccination, p.v.) during which they were not infected by SARS-CoV-2 and did not receive a booster dose (Fig. 1a and Table 1). The samples were subject for gut microbiome profiling (shotgun metagenome sequencing), gut metabolome analysis (GC-MS/MS), and immune outcome measurements [including surrogate virus neutralisation test (sVNT) (GenScript) and cytokine and chemokine measurement (LEGENDplex^TM^ assay), detailed in ‘Methods’].

**Table 1:** Demographic characteristics and immune responses to COVID-19 vaccines of the study population

Variable	BNT162b2 (*N* = 121)	CoronaVac (*N* = 40)	*P* value
*Characteristics*			
Age, years	42 (29, 54)	55 (39.75, 57)	0.005
Female^a^	79 (65.3)	29 (72.5)	0.443
BMI, kg/m^2^	21.64 (19.82, 24.46)	22 (20.39, 23.81)	0.725
Overweight or obese^b^	42 (34.7)	16 (40)	0.572
*Presence of comorbidity*			
Hypertension	7 (5.8)	7 (17.5)	0.045
Diabetes mellitus	3 (2.5)	1 (2.5)	1
Allergy history	55 (45.5)	12 (30)	0.098
Diarrhoea (in past 3 months prior to enrolment)	44 (36.7)	14 (35.9)	1
Other comorbidities^c^	16 (13.2)	4 (10)	0.784
*Current medication*			
Antibiotic intake			
Past 3 months prior to enrolment	7 (5.8)	0 (0)	0.194
3 months to 6 months p.v.	3 (3.2)	1 (2.8)	1
Hormone therapy	4 (3.3)	0 (0)	0.573
Immunomodulator	3 (2.5)	1 (2.5)	1
Probiotics	14 (11.7)	6 (15)	0.587
*Vaccination in the past year*	56 (46.7)	15 (37.5)	0.361
*Dietary habit*			
Vegetarian	2 (1.7)	0 (0)	1
*Diet change*			
During vaccination	0 (0)	0 (0)	N.A.
3 months to 6 months p.v.	2 (2.2)	0 (0)	1
*Alcohol intake (within 2 weeks prior to first vaccine dose)*	32 (26.4)	6 (15)	0.197
*Regular exercise (strenuous/moderate)*	70 (57.9)	26 (65)	0.462
*SARS-CoV-2 antibody response*			
sVNT (inhibition %) 1 month p.v.	95.3 (93.1, 96.5)	59.0 (43.7, 72.6)	<0.001
sVNT (inhibition %) 6 months p.v.	63.1 (49.1, 79.3)	6.1 (−0.7, 18.5)	<0.001
Relative drop in sVNT from 1 to 6 months p.v.	34.1 (16.6, 47.3)	88.9 (74.2, 102.2)	<0.001
*Adverse events after the first dose*^d^			<0.001
0	10 (8.5)	16 (40)	
1	44 (37.3)	16 (40)	
2	26 (22)	4 (10)	
≥3	38 (32.2)	4 (10)	
*Adverse events after the second dose*^d^			<0.001
0	5 (4.2)	14 (35)	
1	27 (22.9)	15 (37.5)	
2	22 (18.6)	5 (12.5)	
≥3	64 (54.2)	6 (15)	

Categorical data are presented as number (percentage) and continuous data as median (IQR). Within-group valid percentages are shown

*BMI* body mass index, *sVNT* surrogate virus neutralisation test

^a^One participant requested concealment of gender

^b^BMI between 23.0 and 25.0 kg/m^2^ is classified as overweight and BMI above 25.0 kg/m^2^ is classified as obese

^c^Other comorbidities: asthma, depression, eczema, high cholesterol, systemic lupus erythematosus, attention deficit hyperactivity disorder

^d^Adverse events: injection site pain/burn, fatigue, fever, injection site swelling/pruritus/erythema/induration, myalgia, drowsiness, headache, chills, dizziness, arthralgia, loss of appetite, abdominal pain, rhinorrhea, sore throat, diarrhoea, pruritus, coughing, constipation, abdominal distension, nausea, flushing, hypersensitivity, muscle spasms, nasal congestion, oedema, vomiting, tremor, eyelid oedema, nosebleeds, hyposmia, ocular congestion, lower back pain, increase in appetite, muscle pain, rib pain, eye pain, palpitations

Consistent with another Hong Kong-based epidemiological study,^13^ immunogenicity to the BNT162b2 vaccine was stronger and more durable than to the CoronaVac vaccine. At 6 months p.v., 57.3% of BNT162b2 vaccinees had an sVNT level greater than 60%, a threshold corresponding to twice the 50% protection titre^14^; all CoronaVac vaccinees had sVNT levels below this threshold (Fig. 1b). They were positively correlated to immunogenicity 1 month p.v. (BNT162b2: Spearman’s Rho = 0.59, *p* < 0.001; CoronaVac: Spearman’s Rho = 0.74, *p* < 0.001). Moreover, sVNT levels induced by the CoronaVac vaccine decreased faster than that of the BNT162b2 vaccine (*P* < 0.001, Fig. 1c).

Plasma cytokine and chemokine levels, measured by flow cytometry (LEGENDplex^TM^ assay, BioLegend, USA), were more stable following vaccination in the BNT162b2 group compared to the CoronaVac group, as evident from correlations between baseline and 1-month readouts (Supplementary Fig. 1). Durable immunogenicity, reflected by sVNT levels at 6 months p.v., was negatively associated with plasma monocyte chemoattractant protein-1(MCP-1), interferon (IFN)- γ, tumour necrosis factor (TNF)- α, interleukin (IL)-5, IL-13, IL-22, and eotaxin levels at baseline among CoronaVac vaccinees (FDR < 0.1, Supplementary Table 1, and Supplementary Fig. 2). sVNT levels at 6 months p.v. were not significantly associated with baseline plasma cytokine levels in the BNT162b2 group (Supplementary Table 1).

Interestingly, receiving vaccines other than those for SARS-CoV-2 (mostly influenza) was associated with a positive effect on the durable immunity to CoronaVac (*P* = 0.017), but a negative effect on the BNT162b2 one (*P* = 0.009) (Fig. 1d and Supplementary Table 2). These correlations remained statistically significant in multivariable modellings for both vaccine groups (Supplementary Table 3).

### Baseline gut microbiome composition and gut metabolome predicted durable immunity at 6 months post-vaccination

**Fig. 2:**
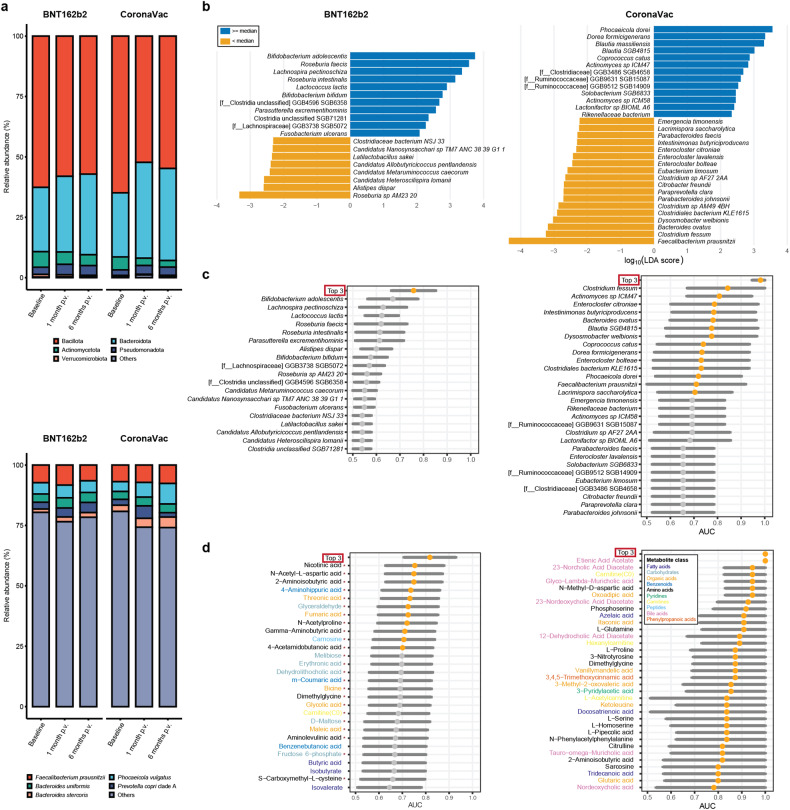
Baseline gut microbiome and metabolome biomarkers of sVNT levels to the BNT162b2 and CoronaVac vaccines at 6 months p.v. **a** Average relative abundances of the five most abundant and other bacterial phyla (upper panel) and species (lower panel). **b** Baseline gut microbiome biomarkers detected by LEfSe. **c** AUROC (95% CI) values of models based on individual microbiome biomarkers and a combined model based on the top three most predictive biomarkers for high vs. low sVNT levels at 6 months p.v. **d** AUROC (95% CI) values of models based on individual metabolite biomarkers and a combined model based on the top three most predictive metabolite biomarkers for high vs. low sVNT levels at 6 months p.v. Only markers with a lower bound for the AUROC of >0.5 were shown. An AUROC of >0.7 was presented as an orange dot. Red asterisks denote metabolites that were positively associated with durable immunity. Elements on forest plots: centre dot, mean AUROC; horizontal line, 95% confidence interval

In both vaccine groups, Bacillota, Bacteroidota, Actinomycetota, Pseudomonadota, and Verrucomicrobiota comprised >99% of the gut microbiota; Bacteroidota and Pseudomonadota increased, while Bacillota and Actinomycetota decreased in relative abundances following vaccinations (FDR < 0.1) (Fig. 2a, Supplementary Fig. 3, and Supplementary Table 4). The overall compositions of the microbiota did not differ between the two vaccine groups (Supplementary Fig. 4), nor did they correlate with age (Supplementary Fig. 5).

We first aimed to assess associations between baseline gut microbiota composition and vaccine durability. Among BNT162b2 vaccinees, higher relative abundances of *Roseburia faecis*, *B. adolescentis*, and *B. bifidum* at baseline were associated with higher sVNT levels at 6 months p.v., as identified by linear discriminant analyses (LEfSe, Methods) (Fig. 2b, Supplementary Fig. 6, and Supplementary Table 5). Using a combination of the top three bacteria species (*B. adolescentis*, *Lachnospira pectinoschiza*, and *Lactococcus lactis*) associated with 6-month sVNT values, we were able to differentiate subjects with high vaccine response from those with low vaccine response at 6 months with an area under the receiver operating characteristic curve (AUROC) of 0.758 (95% CI: 0.664, 0.851) (Methods, Fig. 2c). Among the CoronaVac group, higher relative abundances of short chain fatty acid (SCFA)-producing bacteria *Phocaeicola dorei*, *Blautia massiliensis*, and *Dorea formicigenerans* at baseline were associated with higher sVNT levels at 6 months p.v. (identified by LEfSe, Fig. 2b, Supplementary Fig. 7, and Supplementary Table 5). Surprisingly, the relative abundance of *Faecalibacterium prausnitzii* was negatively associated with sVNT levels at 6 months p.v. (Fig. 2b). The top three bacteria species (*Clostridium fessum*, *Actinomyces* sp. ICM47, and *Enterocloster citroniae*) collectively distinguished high vs. low sVNT levels at 6 months with an AUROC of 0.982 (95% CI: 0.947, 1.000) (Fig. 2c). Only two of these potential marker species appeared to be associated with age (uncorrected *P* value <0.05, Supplementary Table 6), but neither of them was top-ranking predictors in the modelling (Fig. 2c). In addition, 16 of 19 (84.2%) and 21 of 31 (67.7%) potential species markers in the BNT162b2 and CoronaVac groups, respectively, were also identified by additional compositionality-aware differential abundance analyses based on centred log-ratio transformed abundances (ALDEx2 and/or LINDA, Methods) (uncorrected *P* value < 0.05, Supplementary Tables 7 and 8).

We then identified correlations between durable vaccine responses and baseline gut metabolome quantified by liquid chromatography-tandem mass spectrometry (LC-MS/MS (Supplementary text), compositions of which were similar between the vaccine groups (Supplementary Fig. 4) and were not associated with age (Supplementary Fig. 5). Among the BNT162b2 group, we identified 28 differential metabolites between participants with high vs. low sVNT levels 6 months p.v., including nicotinic acid (Vitamin B), γ-Aminobutyric acid (GABA), fumaric acid, 2-Aminoisobutyric acid, m-Coumaric acid (an antioxidant), and threonic acid (a metabolite of vitamin C), that were enriched in high responders; butyrate, isobutyrate, isovalerate, and benzenebutanoic acid were elevated in low responders (Supplementary Table 9, ‘Methods’). None of these metabolites was associated with age (Supplementary Table 10). Among these metabolites, nicotinic acid was the most predictive of high vs. low sVNT levels at 6 months p.v., with an AUROC of 0.753 (95% CI: 0.629, 0.878; Fig. 2d).

Among CoronaVac vaccinees, we identified 42 potential metabolite markers for high vs. low sVNT levels at 6 months. These metabolites included L-glutamine, 2-Aminoisobutyric acid, fumaric acid, and L-tryptophan, the concentrations of which were all negatively associated with sVNT levels at 6 months p.v. (Supplementary Table 11). Of note, the concentrations of etienic acid acetate at baseline in the high-sVNT were all lower than those of the low-sVNT group (Fig. 2d). In addition, only one of these potential markers (Itaconic acid) was associated with age (Spearman’s Rho = 0.49, *P* = 0.047, Supplementary Table 12). Overall, the metabolites identified from CoronaVac vaccinees performed better than those identified from BNT162b2 vaccinees in classifying participants with high vs. low sVNT levels at 6 months p.v. (Fig. 2c, lower panel).

We also conducted pathway enrichment analyses of the identified metabolite markers to investigate potential metabolic pathways associated with sVNT levels. For both vaccines, the pathway for alanine/aspartate/glutamate metabolism was associated with higher sVNT levels at 6 months p.v. (Supplementary Fig. 8). The pathway for glycine/serine/threonine metabolism was implicated in both vaccine groups: it was correlated with higher BNT162b2 sVNT levels but lower CoronaVac sVNT levels at 6 months p.v. (Supplementary Fig. 8). In addition, metabolite markers of better BNT162b2 durable immunogenicity were enriched in a starch and sucrose metabolic pathway. As for CoronaVac durable immunogenicity, the positively associated metabolites were enriched in pathways for butanoate metabolism, propanoate metabolism, and the citric acid cycle; negative correlates were enriched in a pathway for propanoate metabolism and depleted in pathways responsible for tryptophan metabolism, D-glutamine and D-glutamate metabolism, and purine metabolism, among others (Supplementary Fig. 8).

Since baseline *B. adolescentis* relative abundance was positively associated with sVNT levels to BNT162b2 at 6 months p.v., we next examined the correlations between the identified metabolite markers and the relative abundance of *B. adolescentis* at baseline. We found that *B. adolescentis* relative abundance was significantly proportional to the concentrations of m-Coumaric acid (Rho = 0.32, *P* = 0.004, FDR = 0.037), threonic acid (Rho = 0.32, *P* = 0.004, FDR = 0.037), and GABA (Rho = 0.24, *P* = 0.038, FDR = 0.106), among others (Supplementary Table 13 and Supplementary Fig. 9).

### Gut microbiome at 1 month p.v. correlated with sVNT levels to CoronaVac at 6 months post vaccination

Since we have previously observed changes in gut microbiome composition at 1 month post-COVID-19 vaccination,^7^ we wanted to investigate the associations between the 1-month microbiome and durable immunity to vaccination.

**Fig. 3:**
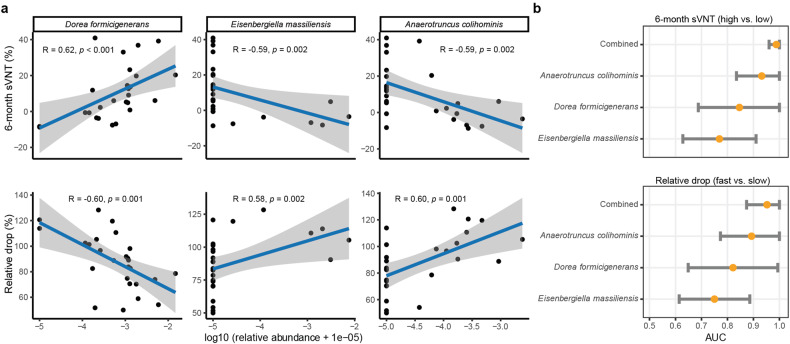
Correlations between bacterial relative abundances at 1 month and sVNT levels to the CoronaVac vaccine at 6 months p.v. **a** Correlations between bacterial relative abundances at 1 month p.v. and sVNT levels to CoronaVac at 6 months p.v. Coefficients and *P* values of the correlations were given by Spearman’s correlation tests. **b** Predictive power of bacterial relative abundances at 1 month on sVNT levels to CoronaVac at 6 months p.v. Elements on forest plots: centre dot, point estimate of the effect size; horizontal line, 95% confidence interval

Interestingly, among CoronaVac vaccinees, a higher relative abundance of *Dorea formicigenerans* at 1 month p.v. was associated with higher sVNT levels (Rho = 0.62, *P* = 0.001, FDR = 0.123) and lesser drop in sVNT levels at 6 months p.v. (Rho = −0.60, *P* = 0.001, FDR = 0.123), correlations stronger than those for relative abundance of the same species at baseline (Fig. 3a and Supplementary Table 14). In addition, 1-month relative abundances of *Eisenbergiella massiliensis* and *Anaerotruncus colihominis*, two bacterial species less abundant in human adults, correlated negatively with sVNT levels at 6 months (Rho = −0.59, *P* = 0.002, FDR = 0.123, and Rho = −0.58, *P* = 0.002, FDR = 0.123, respectively) and positively with reduction in sVNT from 1 month to 6 months p.v. (Rho = 0.58, *P* = 0.002, FDR = 0.128, and Rho = 0.60, *P* = 0.001, FDR = 0.123, respectively) (Supplementary Table 14). Among them, *A colihominis* outperformed the other two in predicting 6-month sVNT values, with an AUROC of 0.932 (95% CI: 0.835, 1.000). Combining the relative abundances of these three species further improve the prediction [AUROC = 0.960 (0.988, 1.000)] (Fig. 3b).

Among BNT162b2 vaccinees, correlations between bacterial relative abundance at 1 month p.v. and 6-month immune outcomes were generally weak, with the strongest being Rho = ±0.20 (FDR = 0.976–0.982) (Supplementary Table 15).

### Gut microbiome alterations and recovery after vaccination

**Fig. 4:**
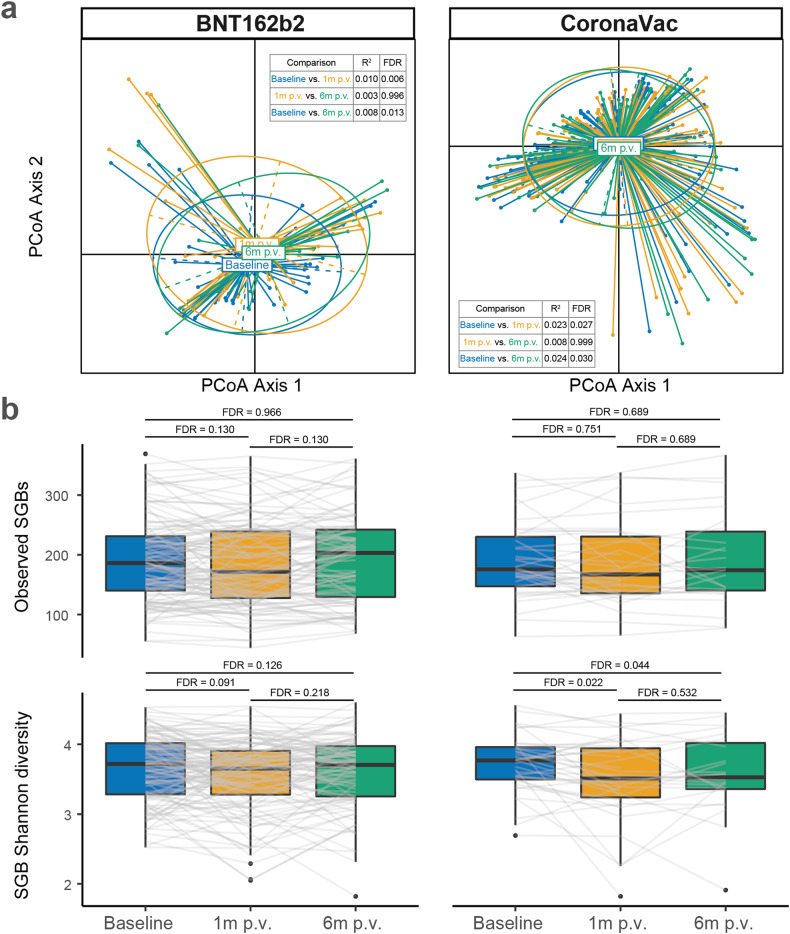
Gut microbial beta diversity and alpha diversity from baseline to 6 months post vaccination. **a** Beta diversity of the gut microbiota from baseline to 6 months p.v. *R*^2^ and *P* values were given by permutational multivariate analysis of variance (PerMANOVA). **b** Alpha diversity (observed species-level genome bins and Shannon diversity of species-level genome bins) indices of the gut microbiota from baseline to 6 months p.v. *P* values were given by paired Wilcoxon’s rank-sum tests and were FDR-corrected. Elements on boxplots: centre line, median; box limits, upper and lower quartiles; whiskers, 1.5×IQR; points, outliers. SGB: species-level genome bin

We further characterised gut microbiome alterations at 6 months p.v. In both vaccine groups, overall alterations of gut microbiota composition did not recover to baseline at 6 months p.v. (Fig. 4a). Alpha and Shannon diversity dropped significantly at 1 month p.v. in both vaccine groups. Microbial diversity recovered to that of baseline levels at 6 months p.v. in the BNT162b2 group (Fig. 4b). In contrast, the number of observed species-level genome bins did not vary significantly between time points for both vaccine groups (Fig. 4b).

**Fig. 5:**
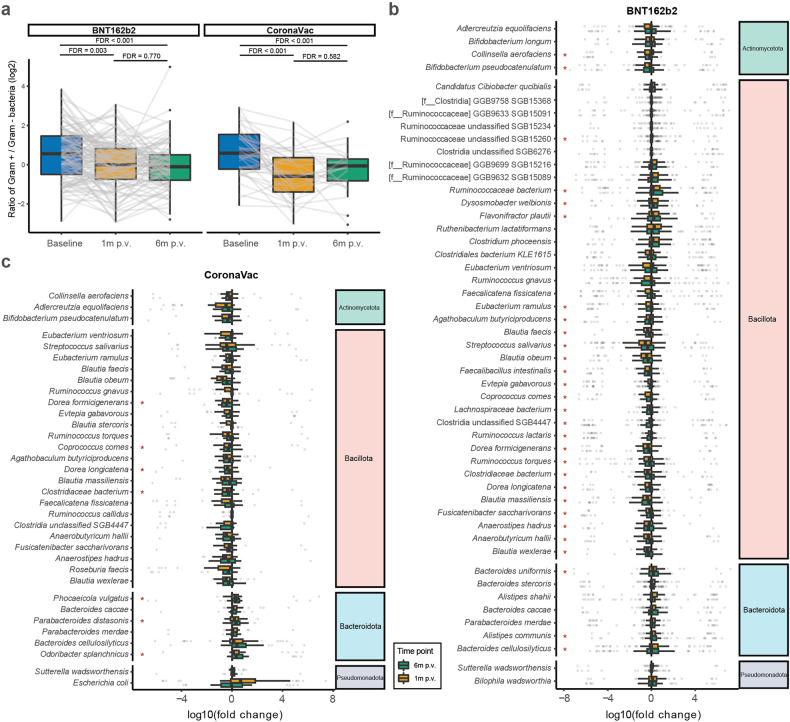
Vaccination-induced changes in gut Gram+/Gram− ratio and species abundance. **a** Ratios of Gram+/Gram– bacteria from baseline to 6 months p.v. (log2-transformed). *P* values were given by paired Wilcoxon’s rank-sum tests and were FDR-corrected. **b** Fold changes of abundances of species altered at 1 month vs. baseline in the BNT162b2 group. **c** Fold changes of abundances of species altered at 1 month vs. baseline in the CoronaVac group. Red asterisks denote species that did not recover to baseline at 6 months p.v. Elements on boxplots: centre line, median; box limits, upper and lower quartiles; whiskers, 1.5×IQR; points, outliers

There was a lower ratio of Gram+ vs. Gram- bacteria in both vaccine groups following vaccination (BNT162b2: FDR = 0.003, CoronaVac: FDR < 0.001) and this persisted into 6 months p.v. (FDR < 0.001 in both groups) (Fig. 5a). This may likely be attributed to the decreases in the relative abundances Gram+ Actinomycetota and Bacillota members and the increases in the relative abundances of Gram− Pseudomonadota and Bacteroidota members at 1 month p.v. (Fig. 5b, c). Twenty-seven of these species whose relative abundances varied were consistently differentially abundant in both vaccine groups (Supplementary Table 16), the majority of which were independent of age (Supplementary Table 17). While 79.4% (27 of 34) of the altered species recovered at 6 months p.v. in CoronaVac vaccinees (Fig. 5b), more than half of those in BNT162b2 vaccinees did not (58.0%, 29 of 50). These slow-recovering species in the BNT162b2 group were mainly Bacillota members (*n* = 20, 83.3%) (Fig. 5c). In addition, among the slow-recovering species, 5 of 7 (71.4%) in the CoronaVac group and 9 of 29 (31.0%) in the BNT162b2 group were reported to be differentially abundant between patients with and without post-acute COVID-19 syndrome at 6 months post infection,^15^ including *Dorea longicatena*, *Dorea formicigenerans*, and *Coprococcus comes*.

As for microbial functional pathways, 75 pathways were significantly changed in 1 month p.v. compared to baseline, of which 45 (60.0%) did not recovered to baseline in 6 months p.v. (FDR < 0.1, Supplementary Table 18) in the BNT162b2 group. The CoronaVac group saw a similar pattern, that 88 pathways were significantly changed in 1 month p.v. and 59 (67.0%) of them did not recovered to baseline in 6 months p.v. (FDR < 0.1, Supplementary Table 19). Among these pathways, 52 were changed in 1 month p.v. in both the BNT162b2 and CoronaVac groups. Of note, all of these pathways changed in the same direction in both vaccine groups, except a pathway responsible for dTDP-beta-L-rhamnose biosynthesis (decreased in BNT162b2 vaccinees but increased in CoronaVac vaccinees in 1 month compared to baseline). In both groups, several pathways responsible for histidine biosynthesis depleted, while several for degradations of methionine and arginine elevated in relative abundances. In addition, two pathways related to SCFA formation, including P41-PWY: pyruvate fermentation to acetate and (S)-lactate I, and PWY-5100: pyruvate fermentation to acetate and lactate II, were depleted in both vaccine groups after vaccination compared to baseline.

Finally, the proportion of sequencing reads that could not be classified to known prokaryotic and eukaryotic microbes was greater at 6 months p.v. compared to baseline (BNT162b2, FDR = 0.003; CoronaVac, FDR = 0.046) (Supplementary Fig. 10). In the CoronaVac group, this may have been due to an increase in total viral abundance (FDR = 0.069), a phenomenon that was coupled with a depletion in virome Shannon diversity (FDR = 0.050) at 6 months p.v. (Supplementary Fig. 10). This mirrored the dynamics of bacterial diversity (Fig. 5b and Supplementary Fig. 11).

## Discussion

To our knowledge, this is the first human study to have identified that gut microbiome and metabolome composition at baseline can potentially predict SARS-CoV-2 neutralising antibody levels up to 6 months after two doses of COVID-19 vaccines. We demonstrated that beneficial components of our gut microbiome, such as *B. adolescentis*, can play a role in modulating the durability of immunity to the BNT162b2 vaccine for 6 months and likely even longer, to overcome waning immune responses over time.

Previously, we reported within the same cohort that SARS-CoV-2 sVNT levels at 1 month p.v. were associated with baseline gut microbiota composition, including positive associations between *R. faecis* relative abundance and BNT162b2 immunogenicity, and between *B. adolescentis* relative abundance and CoronaVac immunogenicity. Here, we followed up the cohort to further establish the immune-modulating roles of baseline gut microbiota over a longer term. In the BNT162b2 group, the baseline relative abundances of *B. adolescentis* and *B. bifidum* were surprisingly positively associated with sVNT levels at 6 months p.v. Their relative abundances were, however, not associated with sVNT levels at 1 month p.v. in the same vaccine group.^7^ Perhaps, the beneficial roles of these two species were masked at 1 month by the high immunogenicity of BNT162b2 and can only be observed at a later stage. In addition, the relative abundances of *R. faecis* and *R. intestinalis*, two species with anti-inflammatory properties,^16^ were positively associated with sVNT levels at 6 months p.v. to BNT162b2. Supplementation of these bacteria may therefore help with maintaining immunity to BNT162b2. In the CoronaVac group, a higher sVNT at 6 months p.v. was associated with higher relative abundances of *Phocaeicola* (*Bacteroides*) *dorei* and lower *Faecalibacterium prausnitzii* at baseline, a pattern that was also observed among COVID-19 patients when compared to uninfected controls.^17^ Since our cohort participants had no history of SARS-CoV-2 infection at enrolment, this baseline gut microbiota signature might not be related to viral infection, but it could prime CoronaVac recipients for more durable immune responses.

Among the metabolites that were positively associated with durable immunity to BNT162b2, fumaric acid, which is derived from dimethyl fumarate, can exert anti-inflammatory therapeutic and neuroprotective effects via a nuclear factor-erythroid factor 2-related factor 2 (Nrf2)-dependent mechanism, inhibiting SARS-CoV-2 replication in vitro.^18,19^ GABA biosynthesis in arginine and proline metabolism was also positively correlated to the durability of the immune response in the BNT162b2 group. Primarily a neurotransmitter, GABA plays an anti-inflammatory role and agonists of the GABA receptor have been shown to reduce viral load in SARS-CoV-2-infected mice.^20^ Interestingly, the relative abundance of *B. adolescentis*, a key producer of GABA in the human gut^21^ was positively associated with GABA concentration in our study. In addition, the negative associations between the baseline concentrations of two branched-chain amino acids, isobutyric acid and isovaleric acid, and immune responses to BNT162b2 were consistent with observations described in a recent study.^8^

Among CoronaVac vaccinees, stool tryptophan concentration was negatively associated with durable immunity to the vaccine. A recent study has described how inhibiting the conversion of plasma tryptophan into kynurenine led to a marked decline in plasma pro-inflammatory cytokines in a *Rhesus macaque* model, potentially controlling cytokine release syndrome during SARS-CoV-2 infection.^22^ Moreover, in the CoronaVac vaccine group, those who had a higher sVNT level at 6 months p.v. had a lower baseline concentration of etienic acid acetate, a metabolite of deoxycorticosterone. This phenomenon corroborates the observed negative association between deoxycorticosterone levels at admission and COVID-19 disease severity,^23^ with the latter being positively associated with SARS-CoV-2 neutralising antibody levels among COVID-19 patients.^24^ Future studies are warranted to validate the prediction power of both the species and metabolite markers, as we could not identify a qualified independent cohort currently (Supplementary text, Supplementary Fig. 12).

We further showed that abundances of microbial species at 1 month p.v. were associated with durable immunogenicity to CoronaVac, but not to BNT162b2. Specifically, a higher sVNT level at 6 months p.v. was associated with a higher relative abundance of *D. formicigenerans* and lower relative abundancess of *E. massiliensis* and *A. colihominis* in CoronaVac vaccinees. *A. colihominis* was less frequently found in healthy individuals.^25^ This species was also more abundant in the gut of melanoma patients who did not respond to anti-PD-1 immunotherapy,^26^ indicating that its presence may influence the immune system negatively. The use of biotherapeutics specifically targeting this species may therefore improve the durability of immunity.

With samples collected from multiple time points, we were able to describe the persistence and recovery, if any, of alterations in gut microbiome composition following two vaccine doses. Potentially reflecting the different mechanisms of action of the two vaccines, the gut microbiota of the BNT162b2 group recovered faster in alpha diversity but had a greater proportion of altered species that did not recover to baseline levels at 6 months p.v. (58.0%) compared to those induced by CoronaVac (21.6%). However, a greater proportion of these slow-recovering species in the CoronaVac group (71.4%) vs. the BNT162b2 group (31.0%) were associated with post-acute COVID-19 syndrome.^15^ Moreover, among CoronaVac vaccinees, the persistent reduction in microbial diversity coincided with a depletion in gut DNA viral diversity. Interestingly, this loss of both gut microbial and viral diversity has been observed in COVID-19 patients during infection.^27–29^ These findings suggest that gut microbiota alterations related to CoronaVac vaccination, rather than BNT162b2, better resembled those induced by SARS-CoV-2 infection than alterations related to BNT162b2 vaccination. These impacts of vaccines on gut microbiota composition could partly be attributed to cross-reactivity with microbial antigens, as were previously reported.^30,31^ In support of this notion, the alterations in gut microbiota were more profound following vaccination with the inactivated vaccine (diverse viral components as sources of epitopes), vs. vaccination with the mRNA vaccine (the spike protein as the sole source of epitopes). Apart from cross-reactivity, SARS-CoV-2 vaccines could induce systemic inflammation and increase oxidative stress, which can also induce alterations in microbiota.^32^

Some changes in the gut microbiota were common across both vaccine groups: all SARS-CoV-2-vaccinated participants had decreased relative abundances of Bacillota and Actinomycetota, and increased relative abundances of Bacteroidota and Pseudomonadota, corresponding to a reduced gut microbiota Gram+/Gram− ratio post-vaccination. This alteration persisted into the 6^th^ month p.v., indicating different responses and/or resistance to altered host physiology between Gram+ and Gram- bacteria. While the mechanisms are yet to be elucidated, a lower Gram+/Gram− ratio has been proposed as a potential indicator of intestinal inflammation.^33^ In our study, this reduction in Gram+/Gram− ratio was also coupled with a persistent depletion in SCFA-producing Bacillota members, such as *Blautia* spp. and *Dorea* spp. Therefore, this long-term gut microbiota alteration warrants further follow-up investigations. Meanwhile, alterations in the gut microbiome have also been observed in a non-infected and non-vaccinated cohort of the same population during COVID-19.^34^ Therefore, COVID-19 control measures and lifestyle changes, among other factors, may also contributed to these changes.

As for microbial functional pathways, we observed depleted relative abundances of pathways for histidine biosynthesis, and increased relative abundances of pathways for methionine and arginine degradations. These phenomena might indicate increased availability of the corresponding amino acid substrates in the gut following vaccination, perhaps due to altered absorption associated with vaccine-induced inflammation. In addition, the depletion in relative abundances of pathways responsible for converting pyruvate into SCFAs corroborates with another study reporting that microbial pathways responsible for fatty acid biosynthesis and fermentation to SCFAs decreased in relative abundance after the second dose of BBIBP-CorV vaccination.^10^

Interestingly, we found that vaccine history affected durable immunogenicity to both the BNT162b2 and CoronaVac vaccines differently. This, again, is likely due to different mechanisms of action of the vaccines. Immunogenicity to the inactivated vaccine, like live-attenuated vaccines, may benefit from trained-immunity induced by other vaccines.^35^ The mRNA-based vaccine, though having better immunogenicity, may have been hindered by the trained immunity.

Our study is limited by using relative abundances, where the measurement of a taxon’s relative abundance is dependent on the abundance of other taxa. Future studies with absolute quantification are needed. In addition, our current findings are correlative, and future experimental studies are necessary to establish causality and mechanisms. While gut microbiome composition did not fully recover to baseline at 6 months p.v. for both vaccine groups, we are unsure if vaccination permanently changes gut microbiome composition or just that the microbiota takes longer to recover. Longer-term studies are therefore warranted. In addition, as of March 2023, 83.9% of the Hong Kong population had received the third vaccine dose.^36^ How microbiota compositions influence immune responses to booster doses (>2) and vice versa deserve further investigation.

In summary, durable immunity among BNT162b2 vaccinees was correlated with higher *B. adolescentis* relative abundances and higher concentrations of beneficial metabolites at baseline. Meanwhile, a greater immunity to CoronaVac at 6 months was associated with a gut microbiota primed by prior vaccination unrelated to SARS-CoV-2. Compared to gut microbiota alterations following BNT162b2 vaccination, those following CoronaVac vaccination resembled gut microbiota changes induced by SARS-CoV-2 infection more than alterations following BNT162b2 vaccination. The effects of increasing vaccine doses on gut microbiota composition, its recovery, and long-term health in both vaccine groups deserve further monitoring.

## Materials and methods

### Study cohort

Subject recruitment, demographic data collection, and sample processing have been discussed in detail in our previous paper.^7^ Briefly, between April 2021 and June 2021, we recruited healthy subjects from two vaccination centres in Hong Kong who were aged 18 years or above receiving the BNT162b2 (*N* = 121) or CoronaVac vaccine (*N* = 40). Exclusion criteria included prior COVID-19 infection, gastrointestinal surgery, IBD, immunocompromised status, and the use of antibiotics, probiotics, or proton pump inhibitors in the preceding month. At baseline, 1 month after the second vaccine dose (post vaccination, p.v.), and 6 months p.v., stool samples were self-collected in a DNA preservative tube at home and transferred at room temperature to laboratories within an average of 48 h and stored at −80 °C until DNA extraction; blood samples were collected at hospital clinics and transported to laboratories for the separation of plasma for serological tests. All participants provided written informed consent and completed two doses of vaccines.

### Ethics approval statements

The study was approved by The Joint Chinese University of Hong Kong – New Territories East Cluster Clinical Research Ethics Committee (The Joint CUHK-NTEC CREC) (2021.260) and The Institutional Review Board of the University of Hong Kong/Hospital Authority Hong Kong West Cluster (HKU/HA HKW) (UW 21–203). The study was conducted in accordance with the Declaration of Helsinki (1975) and Good Clinical Practice.

### Blood samples sVNT and cytokine/chemokine measurements

Blood samples were subjected to SARS-CoV-2 surrogate virus neutralisation test (sVNT) (GenScript, NJ, USA, Catalogue No. L00847-A) and cytokine and chemokine measurements (LEGENDplex^TM^ assay, BioLegend, USA, Catalogue No. 741027 and 740984, respectively) as per the manufacturer’s instructions. sVNT data was expressed as percentage inhibition (%). Durable immunity to the vaccines was described by two dimensions: (1) sVNT level at 6 months p.v. and (2) relative drop in sVNT from 1 month to 6 months p.v. In addition, sVNT levels were dichotomised into high (>median) vs. low (≤median) at 6 months p.v. (63.1% and 6.1% for BNT162b2 and CoronaVac, respectively). Cytokine and chemokine data were normalised by calculating their ratios to the readouts of negative controls.

### Stool samples, metagenomic and metabolomic analysis

DNA from the stool samples were extracted and sequenced on the Illumina NovaSeq 6000 platform (250/150 base pairs paired-end) at Microbiota I-Center, Hong Kong, China (Supplementary text). Following quality control and pre-processing of the sequencing data (Supplementary text), the microbiota was profiled with MetaPhlAn4^37^ with reference to the CHOCOPhlAnSGB database (vJan21_202103) using default settings. Based on profiles of species-level genome bins (SGBs), alpha (and Shannon diversity) and beta diversity (Bray–Curtis dissimilarity) indices were calculated. Gram positive (+) and Gram negative (−) species were identified per the JGI Genome Online Database (GOLD).^38^ The Gram+/Gram− ratio was then calculated based on the total relative abundances of these two bacterial groups. Gut viromes were analysed using CoverM (https://github.com/wwood/CoverM), referencing the Gut Virome Database.^39^ For each viral genome, coverage depth was averaged, with the 5% of bases with the highest and lowest depths removed.^39^ Relative abundance was then attained by normalising the averaged depth to 1GB sequencing bases. Those with a relative abundance of <0.1 were considered absent in the sample (relative abundance set as 0).

Stool metabolomes were profiled by liquid chromatography-tandem mass spectrometry (LC-MS/MS) using Waters UPLC I-Class Plus (Waters, USA) equipped with QTRAP 6500 Plus (SCIEX, USA), targeting around 400 metabolites and 10 C2-C6 short-chain fatty acids in human faecal samples (Supplementary text).

### Statistical methods

Comparisons of data between two groups were done using Fisher’s exact test for categorical variables and Wilcoxon rank-sum test for continuous variables. Paired Wilcoxon rank-sum tests (two sided) were performed to compare continuous variables measured at different time points from the same subjects. Correlations between continuous variables were analysed using Spearman’s correlation tests. In these correlation tests, relative abundances less than 0.001% were considered as 0, and only those that were present in 25% samples were included (except for the correlation analysis for baseline species markers). Based on linear discriminant analysis (LDA) effect size (LEfSe), differentially abundant species between groups were identified as those with a LDA score >2 and *P* < 0.05.^40^ In a sensitivity analysis, read count profiles with centred log-ration transformation were analysed by compositionality-aware methods, including ANOVA-like differential expression (ALDEx2) analysis^41^ and linear regression framework for differential abundance analysis (LINDA)^42^ with default parameters. Metabolite markers for binary sVNT levels were identified as those with Variable Importance in the Projection (VIP) > 1, a fold change <0.8 or >1.25, and a *P* value of <0.05 in the Wilcoxon’s rank-sum test. Pathway enrichment analyses were conducted based on differential metabolites between groups using MetaboAnalystR. Generalised linear regression modelling (GLM) was used to examine the correlations between demographic factors, gut microbiota (relative abundance or colonisation) and metabolome, and sVNT outcome. The predictive power of baseline bacterial and metabolite biomarkers for high vs. low sVNT levels was assessed based on the area under the receiver operating characteristic curve (AUROC) of each GLM model. False discovery rate (FDR) correction was applied for multiple testings. Analyses where FDR correction were not applicable included binary analysis of demographics, GLM, correlations between *Bifidobacterium adolescentis* abundance and select metabolite markers, and Wilcoxon’s tests of metabolite concentrations. FDR correction was not applicable because these analyses did not include multiple testing, were based on a priori hypotheses, or had several metrics (multivariable and univariable tests) for marker identification.

Data was analysed and visualised in R V4.2.0 with the following packages: tidyverse, dplyr, glm, vegan, ade4, ape, MetaboAnalystR, mixOmics, pROC, ggtext, and ggplot2. *P* values < 0.05 and FDR < 0.1 were considered statistically significant.

### Supplementary information

R3-Supplementary_materials.docx

## Data Availability

Quality-controlled and human DNA-removed sequence data have been deposited into the European Nucleotide Archive under BioProjects PRJEB48269 and PRJEB60773. Additional datasets generated and/or analysed in this study are available from the corresponding author upon reasonable request.

## References

[1] McMenamin ME (2022). Vaccine effectiveness of one, two, and three doses of BNT162b2 and CoronaVac against COVID-19 in Hong Kong: a population-based observational study. Lancet Infect. Dis..

[2] Polack FP (2020). Safety and efficacy of the BNT162b2 mRNA Covid-19 vaccine. N. Engl. J. Med..

[3] World Health Organization. WHO coronavirus (COVID-19) dashboard. https://covid19.who.int/ (WHO, 2023).

[4] Patalon T (2022). Waning effectiveness of the third dose of the BNT162b2 mRNA COVID-19 vaccine. Nat. Commun..

[5] Mentzer AJ (2023). Human leukocyte antigen alleles associate with COVID-19 vaccine immunogenicity and risk of breakthrough infection. Nat. Med..

[6] Ou X (2023). Antibody responses to COVID-19 vaccination in people with obesity: a systematic review and meta-analysis. Influenza Other Respir. Viruses.

[7] Ng, S. C. et al. Gut microbiota composition is associated with SARS-CoV-2 vaccine immunogenicity and adverse events. *Gut* **71**, 1106–1116 (2022). 10.1136/gutjnl-2021-326563 PMC8844967 35140064

[8] Lunken, G. R. et al. Gut microbiome and dietary fibre intake strongly associate with IgG function and maturation following SARS-CoV-2 mRNA vaccination. *Gut*10.1136/gutjnl-2022-328556 (2022).10.1136/gutjnl-2022-32855636549875

[9] Alexander JL (2023). The gut microbiota and metabolome are associated with diminished COVID-19 vaccine-induced antibody responses in immunosuppressed inflammatory bowel disease patients. EBioMedicine.

[10] Tang B (2022). Correlation of gut microbiota and metabolic functions with the antibody response to the BBIBP-CorV vaccine. Cell Rep. Med..

[11] Li X, Watanabe K, Kimura I (2017). Gut microbiota dysbiosis drives and implies novel therapeutic strategies for diabetes mellitus and related metabolic diseases. Front. Immunol..

[12] Sultan S (2021). Metabolic influences of gut microbiota dysbiosis on inflammatory bowel disease. Front. Physiol..

[13] Cowling BJ (2022). Strength and durability of antibody responses to BNT162b2 and CoronaVac. Vaccine.

[14] Lau EH (2021). Long-term persistence of SARS-CoV-2 neutralizing antibody responses after infection and estimates of the duration of protection. EClinicalMedicine.

[15] Liu Q (2022). Gut microbiota dynamics in a prospective cohort of patients with post-acute COVID-19 syndrome. Gut.

[16] Tamanai-Shacoori Z (2017). Roseburia spp.: a marker of health?. Future Microbiol..

[17] Yeoh YK (2021). Gut microbiota composition reflects disease severity and dysfunctional immune responses in patients with COVID-19. Gut.

[18] Linker RA (2011). Fumaric acid esters exert neuroprotective effects in neuroinflammation via activation of the Nrf2 antioxidant pathway. Brain.

[19] Olagnier D (2020). SARS-CoV2-mediated suppression of NRF2-signaling reveals potent antiviral and anti-inflammatory activity of 4-octyl-itaconate and dimethyl fumarate. Nat. Commun..

[20] Tian J., Dillion B. J., Henley J., Comai L., Kaufman D. L. A GABA-receptor agonist reduces pneumonitis severity, viral load, and death rate in SARS-CoV-2-infected mice. *Front. Immunol.* **13**, 1007955 (2022). 10.3389/fimmu.2022.1007955 PMC9640739 36389819

[21] Duranti S (2020). Bifidobacterium adolescentis as a key member of the human gut microbiota in the production of GABA. Sci. Rep..

[22] Xiao N (2021). Integrated cytokine and metabolite analysis reveals immunometabolic reprogramming in COVID-19 patients with therapeutic implications. Nat. Commun..

[23] Sezer S (2022). COVID-19 patients with altered steroid hormone levels are more likely to have higher disease severity. Endocrine.

[24] Garcia-Beltran WF (2021). COVID-19-neutralizing antibodies predict disease severity and survival. Cell.

[25] Gupta VK (2020). A predictive index for health status using species-level gut microbiome profiling. Nat. Commun..

[26] Gopalakrishnan V (2018). Gut microbiome modulates response to anti-PD-1 immunotherapy in melanoma patients. Science.

[27] Cao J (2021). Integrated gut virome and bacteriome dynamics in COVID-19 patients. Gut Microbes.

[28] Lu ZH (2021). Alterations in the composition of intestinal DNA virome in patients with COVID-19. Front. Cell. Infect. Microbiol..

[29] Zuo, T. et al. Alterations in gut microbiota of patients with COVID-19 during time of hospitalization. *Gastroenterology* **159**, 944.8–955.e8 (2020). 10.1053/j.gastro.2020.05.048 PMC7237927 32442562

[30] Li S., Zhou Y., Yan D., Wan Y. An update on the mutual impact between SARS-CoV-2 infection and gut microbiota. *Viruses* **14**, 1774 (2022). 10.3390/v14081774 PMC9415881 36016396

[31] Vojdani A, Vojdani E, Melgar AL, Redd J (2022). Reaction of SARS-CoV-2 antibodies with other pathogens, vaccines, and food antigens. Front. Immunol..

[32] Trougakos IP (2022). Adverse effects of COVID-19 mRNA vaccines: the spike hypothesis. Trends Mol. Med..

[33] Di Pierro, F. Gut microbiota parameters potentially useful in clinical perspective. *Microorganisms* **9**, 2402 (2021). 10.3390/microorganisms9112402 PMC8623243 34835527

[34] Peng, Y. et al. Gut microbiome and resistome changes during the first wave of the COVID-19 pandemic in comparison with pre-pandemic travel-related changes. *J. Travel Med.* **28**, taab067 (2021). 10.1093/jtm/taab067 PMC8135950 33949663

[35] Geckin B, Konstantin Fohse F, Dominguez-Andres J, Netea MG (2022). Trained immunity: implications for vaccination. Curr. Opin. Immunol..

[36] Department of Health for Disease Prevention and Control HKSAR. Hong Kong vaccination dashboard. https://www.coronavirus.gov.hk/eng/index.html (2023).

[37] Blanco-Miguez, A. et al. Extending and improving metagenomic taxonomic profiling with uncharacterized species using MetaPhlAn 4. *Nat. Biotechnol*. 10.1038/s41587-023-01688-w (2023). 10.1038/s41587-023-01688-w PMC10635831 36823356

[38] Mukherjee S (2023). Twenty-five years of Genomes OnLine Database (GOLD): data updates and new features in v.9. Nucleic Acids Res..

[39] Gregory AC (2020). The gut virome database reveals age-dependent patterns of virome diversity in the human gut. Cell Host Microbe.

[40] Segata, N. et al. Metagenomic biomarker discovery and explanation. *Genome Biol.* **12**, R60 (2011). 10.1186/gb-2011-12-6-r60 PMC3218848 21702898

[41] Fernandes AD (2014). Unifying the analysis of high-throughput sequencing datasets: characterizing RNA-seq, 16S rRNA gene sequencing and selective growth experiments by compositional data analysis. Microbiome.

[42] Zhou H, He K, Chen J, Zhang X (2022). LinDA: linear models for differential abundance analysis of microbiome compositional data. Genome Biol..

