# Supplementary material for: Baseline gut microbiota and metabolome predict durable immunogenicity to SARS-CoV-2 vaccines
Source: Signal Transduct Target Ther. 2023 Sep 25;8:373. doi: 10.1038/s41392-023-01629-8 (PMC10518331; doi:10.1038/s41392-023-01629-8)
Supplement: Supplementary file 1 — R3-Supplementary_materials.docx [file 41392_2023_1629_MOESM1_ESM.docx]

Supplementary Materials for

Baseline gut microbiota and metabolome predict durable immunogenicity to SARS-CoV-2 vaccines

Ye Peng^†^, Lin Zhang^†^, Chris KP Mok, Jessica YL Ching, Shilin Zhao, Matthew KL Wong, Jie Zhu, Chunke Chen, Shilan Wang, Shuai Yan, Biyan Qin, Yingzhi Liu, Xi Zhang, Chun Pun Cheung, Pui Kuan Cheong, Ka Long Ip, Adrian CH Fung, Kenneth KY Wong, David SC Hui, Francis KL Chan, Siew C Ng^#^, Hein M Tun^#^

Correspondence to: [heintun@cuhk.edu.hk](mailto:heintun@cuhk.edu.hk) and [siewchienng@cuhk.edu.hk](mailto:siewchienng@cuhk.edu.hk)

**This PDF file includes:**

Materials and Methods

Figures. S1 to S12

Tables S1 to S19

Materials and Methods

SARS-CoV-2 surrogate virus neutralization test (sVNT)

We followed the same procedures as described in our previous paper ^1^, except that a dilution factor of 1:10 was used for plasma samples collected at 6 months post-vaccination for both vaccine groups. Briefly, 10ul of plasma was diluted and mixed with an equal volume of horseradish peroxidase (HRP) conjugated to SARS-CoV-2 spike receptor binding domain (RBD) (6 ng). After incubation for 30 min at 37°C, 100-µl of each mixture was added to each well on the microtiter plate coated with ACE-2 receptor. The plate was sealed and incubated at room temperature for 15 min at 37°C. The plate was then washed with wash solution and 100µl of 3,3’,5,5’- tetramethylbenzidine (TMB) solution was added to each well and incubated in the dark at room temperature for 15 mins. The reaction was stopped by the addition of 50 µl of Stop Solution to each well and the absorbance read at 450 nm in an ELISA microplate reader. The assay validity was based on values representing optical density at 450 nm (OD450) for positive and negative results falling within the range of recommended values. Percentage of inhibition of each plasma was calculated as follows: Inhibition (%) = (1 - sample OD value/negative-control OD value) x 100.

Stool metagenomic analysis

Fecal DNA was extracted from the pellet using Maxwell RSC PureFood GMO and Authentication Kit (Promega, Madison, WI). Briefly, the fecal pellet was added to 1 mL of CTAB buffer and vortexed for 30 seconds, then the sample was heated at 95°C for 5 minutes. After that, the samples were vortexed thoroughly with beads at maximum speed for 15 minutes. Then, 40 µL of proteinase K and 20 µL of RNase A was added to the sample and the mixture was incubated at 70°C for 10 minutes. The supernatant was then obtained by centrifuging at 13,000g for 5 minutes and was added into the Maxwell RSC machine for DNA extraction. Extracted DNA was then subject to DNA libraries construction. This was completed through the processes of end repairing, adding A to tails, purification and PCR amplification using Illumina DNA Preparation kit (Illumina, San Diego, CA).

After sequencing, raw reads were quality filtered and trimmed using KneadData v0.10.0 with Trimmomatic v0.39 to remove adaptor and low-quality sequences (Parametersetting: “MINLEN:50 ILLUMINACLIP:TruSeq3-PE.fa:2:40:15 SLIDINGWINDOW:4:20”) and with Bowtie2 (Parameter settings: “--very-sensitive –dovetail”) to remove human host DNA by mapping reads onto human reference genome GRCh38. Following this, microbiota taxonomic compositions were profiled using MetaPhlAn (v4.0) with default settings. Beta diversity (Bray-Curtis dissimilarity) and alpha diversity indices (observed species genome bins and Shannon diversity index) were calculated based on the species genome bin profiles.

Stool metabolomic analysis

We used high throughput targeted quantification for metabolites kit (HM400 Metabolome) that can realize the absolute quantification of more than 400 metabolite small molecules, including amino acids, organic acids, fatty acids, sugars, bile acids, carnitine, phenyl or benzyl derivatives, and indoles. Briefly, 5 mg of freeze-dried stool samples were reconstituted and homogenised in ultrapure water. This was followed by extraction with internal standards solution and centrifugation at 18000g, 4 °C for 10 minutes to remove debris. For the stool SCFAs, the mixed standards of SCFAs were prepared and gradient dilution was first carried out. Then, 20 µL sample and standard curve sample were collected and 60 µL cold MeOH/ACN (2:1) of standard added. After shaking (5 minutes), precipitating (-20 °C ,4 hours), and centrifugation (20,000 g, 4 °C, 15 minutes), 40 µL supernatant was taken for derivatisation. The solution then was sequentially mixed with 20 μL of 200 mM 3NPH (solvent 50% acetonitrile) and 20 μL of a mixed 120 mM EDC-6% pyridine solution. The mixtures were incubated with shaking at 40 °C for 30 minutes. After the reaction, the mixtures were cooled down to room temperature on ice and centrifuge briefly following dilution by 80 µL 1000 D internal standard. The supernatant was collected and analysed by LC-MS/MS ^2^. Different parameters were set for measuring stool metabolites and SCFAs as follows.

|  | Liquid chromatographic parameters | Mass spectrometry parameters | **Concentration of metabolites** |
| --- | --- | --- | --- |
| HM400 | Column: Waters BEH C18 (model:1.7um*2.1*100mm), column temperature 40℃.  Mobile phase: phase Ultra-pure water (mobile phase A) and acetonitrile (ACN, mobile phase B).  0-1min, 5% mobile phase B；  1-5min, 5%-30% mobile phase B;  5-9min, 30%-50% mobile phase B；  9-11 min, 50%-78% mobile phase B；  11-13.5min, 78%-95% mobile phase B；  13.5-16 min, 95%-100% mobile phase B；  16.1-18 min, 5% mobile phase B.  The flow rate at 0.4mL/min | For the QTRAP 6500 Plus equipped with an EST Turbo Ion-Spray interface, the source parameters were set as follows:  source temperature: 500 °C; ion spray voltage (IS): 4500 V (positive mode) or -4500 V (negative mode); Ion source gas I (GS1), gas II (GS 2) and curtain gas (CUR) were set at 40, 40 and 20 psi, respectively. MRM methods were set at schedule mode with MRM transitions, collision energy (CE), declustering potential energy (DP) and retention time for target metabolites. | The skline (v.21.1.0.146) was used to perform the metabolite identification and quantification.  Software information：  skyline  Version：v.21.1.0.146  Parameter：Mass: Monoisotopic peaks  Mass Tolerance：0.6 Da  Mass Range：50-1500 Da  Official Website：<https://skyline.ms/project/home/software/Skyline/begin.view> |
| SCFAs | Column: Waters BEH C18 (model:1.7 um*2.1*100mm), column temperature 40℃.    Mobile phase:  Phase A: H2O+0.1% formic acid, Phase B: ACN+0.1% formic acid.  The binary solvent elution gradient was optimized at 15% B for 2min, 15%-55% B in 9min, and then held at 100% B for 1min. The column was equilibrated for 3min at 15% B between injections.    The flow rate at 0.35mL/min | Ion source: ESI    Detection mode: negative ion mode    Ion source temperature: 450℃    Detector voltage: -4200 V | In MultiQuant software (SCIEX, USA), the default parameters are used for automatic identification and integration of each MRM transition (ion pair), and manual inspection is assisted.    The content of short-chain fatty acids (ng/mL) equals C.  C is the concentration value (ng/ml) obtained by substituting the integrated peak area of the target index in the sample into the standard curve. |

Systematic review

We conducted a systematic review of literature to identify independent cohort to validate our microbiome-bassed prediction of durable vaccine-induced immunity against SARS-CoV-2. On Jun 29, 2023, five databases (PubMed, EmBase, MEDLINE, Scopus, and Web of Science) and one registry platform (Cochrane) were systematically searched to identify the eligible papers reporting both gut microbiota and immune responses to SARS-CoV-2 vaccines between the 1st of January, 2020, and the 29th of June, 2023. We searched the eligible paper based on the key words as below.

*Vaccination: "Vaccines" OR "COVID-19 Vaccines" OR "SARS-CoV-2 vaccine*"*

*Microbiota : "Microbiota" OR "Gastrointestinal Microbiome" OR "Bacteroides" OR "Bifidobacterium" OR "Lactobacillus" OR microbio*)*

We also screened for papers conneted with (<https://www.connectedpapers.com/>) and those citing our first publication ^1^.

After a series of exclusion (Figure S12), we screened the full text of 19 papers, but none of these studies could provide qualified independent dataset, because of 1) no human gut microbiota profiled (n = 9), 2) no baseline gut microbiota (n = 1), 3) no measurement for levels of neutralizing antibodies against SARS-CoV-2 (n = 2), 4) short follow-ups (n = 6) or being a preprint manuscript of another paper (n = 1). Therefore, whiling acknowledging the importance of validation with external datasets, we are currently not able to perform that.

**References**

1. Ng SC*, et al.* Gut microbiota composition is associated with SARS-CoV-2 vaccine immunogenicity and adverse events. *Gut*, (2022).

2. Xie G*, et al.* A Metabolite Array Technology for Precision Medicine. *Anal Chem* **93**, 5709-5717 (2021).

**Supplementary Figures**

**Figure S1. Correlations of cytokine and chemokine levels between baseline and 1 month p.v.**

 **Figure S2. Significant correlations between baseline cytokine and chemokine levels and 6-month sVNT levels among CoronaVac vaccinees.**

** Figure S3. Major bacterial phyla and species in the collected gut microbiota. a** Relative abundances of the five most abundant and other bacterial phyla in each sample. **b** Relative abundances of the five most abundant and other bacterial species in each sample. The participants are arranged according to the relative abundance of Bacillota in their baseline microbiota.

**
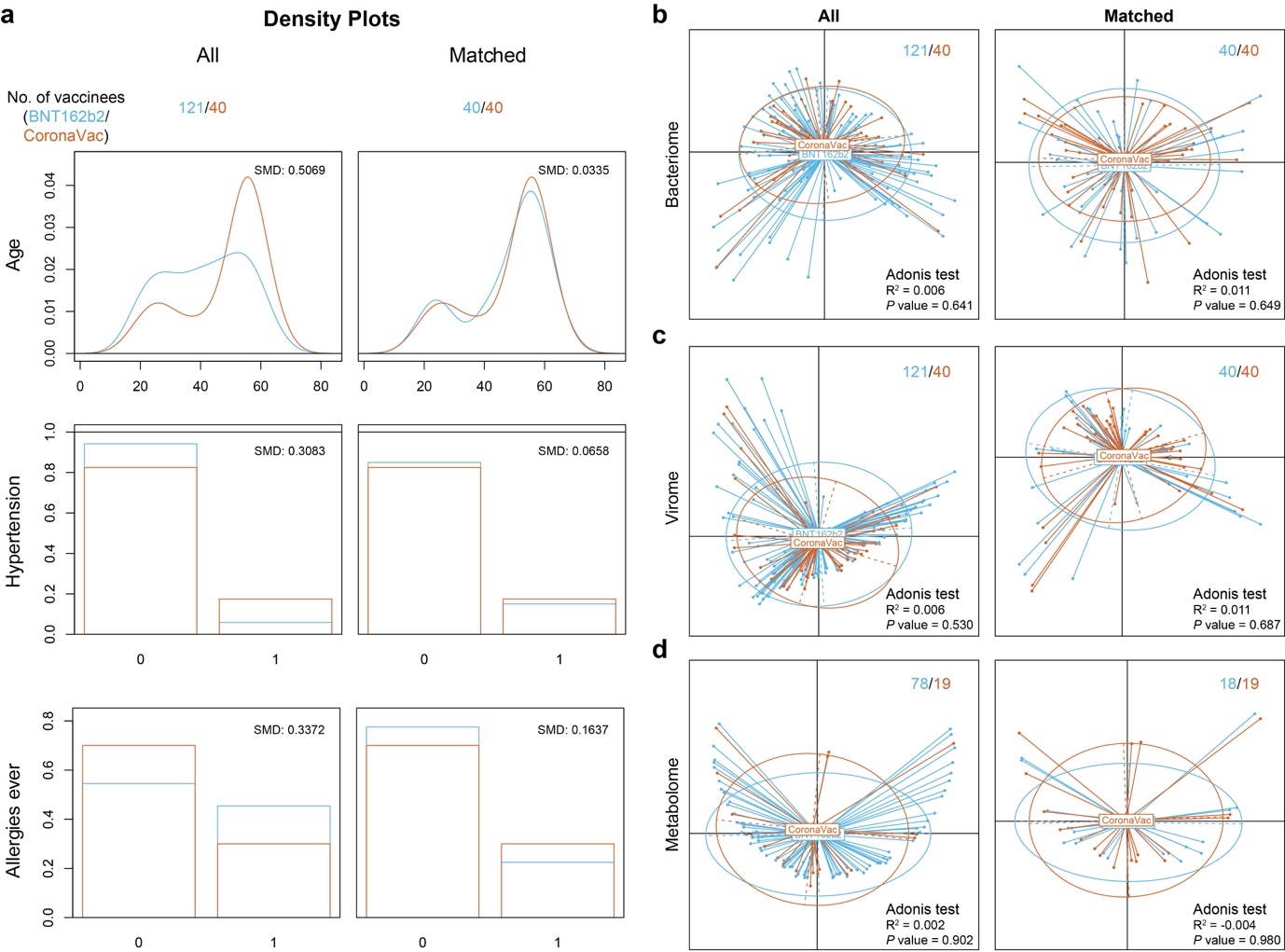
 Figure S4. Comparisons of overall compositions of baseline gut microbiota, virome and metabolome between BNT162b2 and CoronaVac groups.** **a** One-to-one matching of BNT162b2 and CoronaVac vaccinees per age, hypertension and allergy. **b-d** Comparisons of overall compositions of baseline gut microbiota, virome and metabolome between the two vaccine groups in all and matched vaccinees, respectively.

**
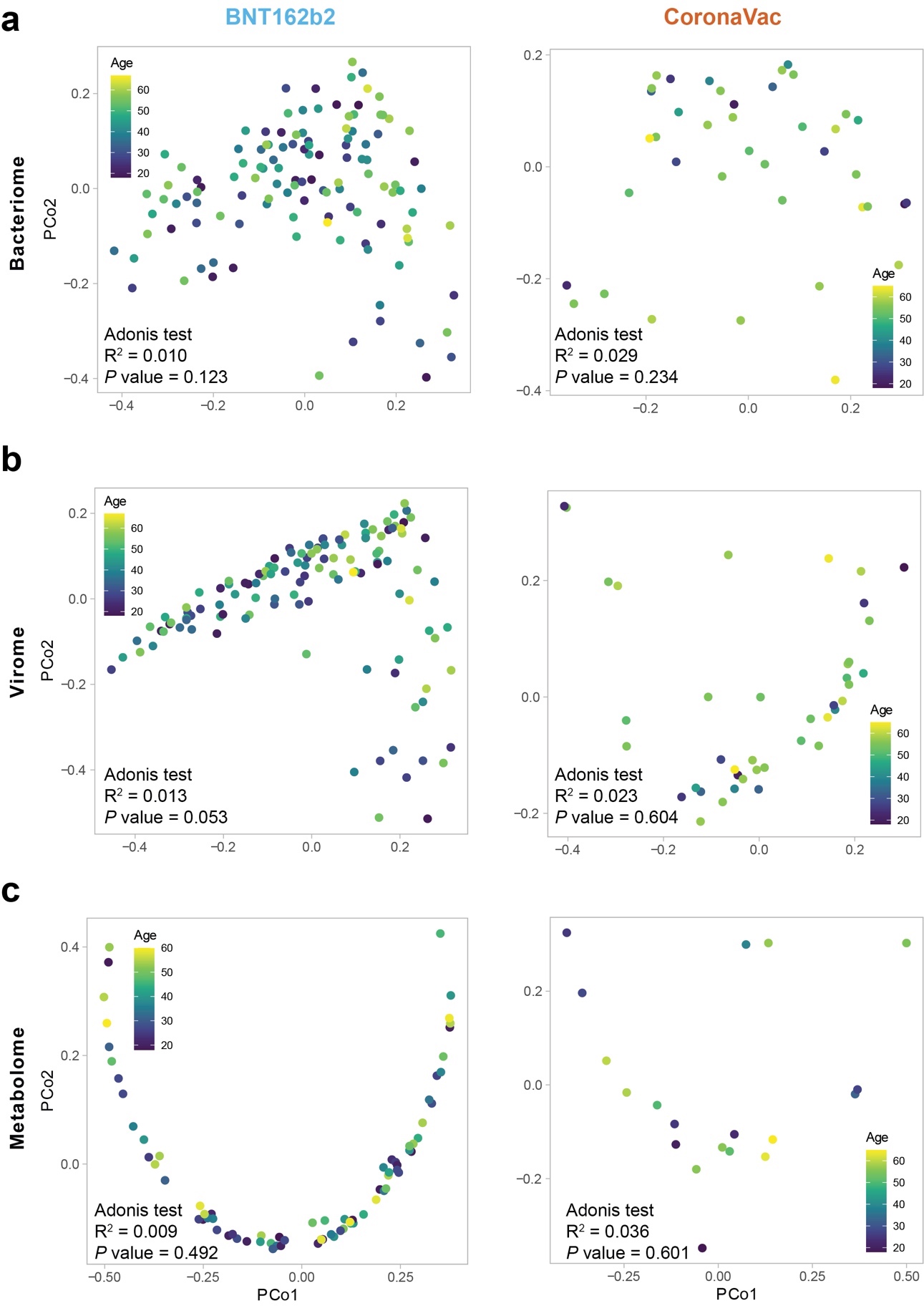
 Figure S5. Correlations between age and overall compositions of baseline gut microbiota, virome and metabolome.** **a** Correlations between age and overall compositions of baseline gut microbiota. **b** Correlations between age and overall compositions of baseline gut virome. **c** Correlations between age and overall compositions of baseline gut metabolome.

**
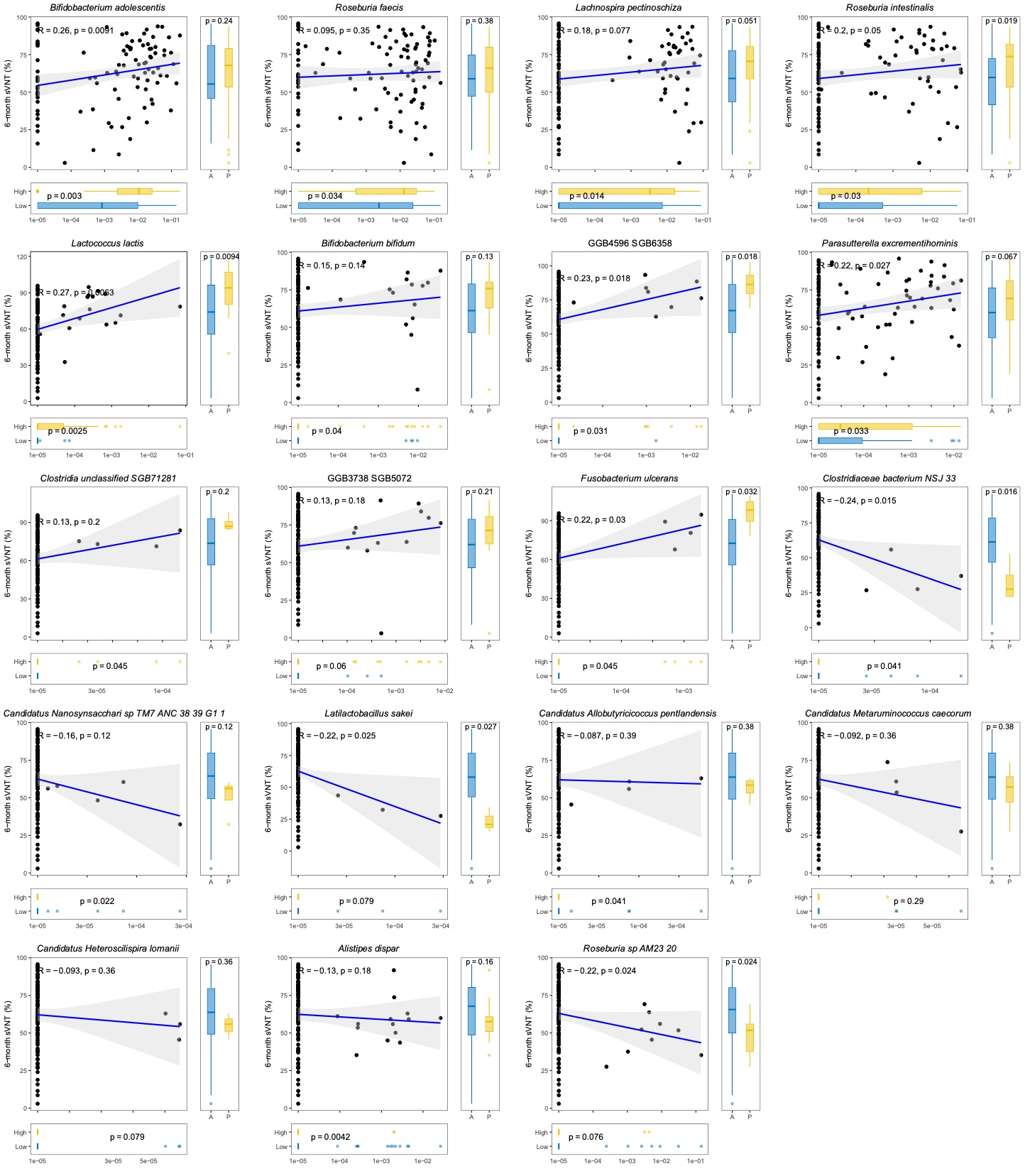
 Figure S6. Correlations between bacterial relative abundances at baseline and 6-month sVNT levels among BNT161b2 vaccinees.**

**
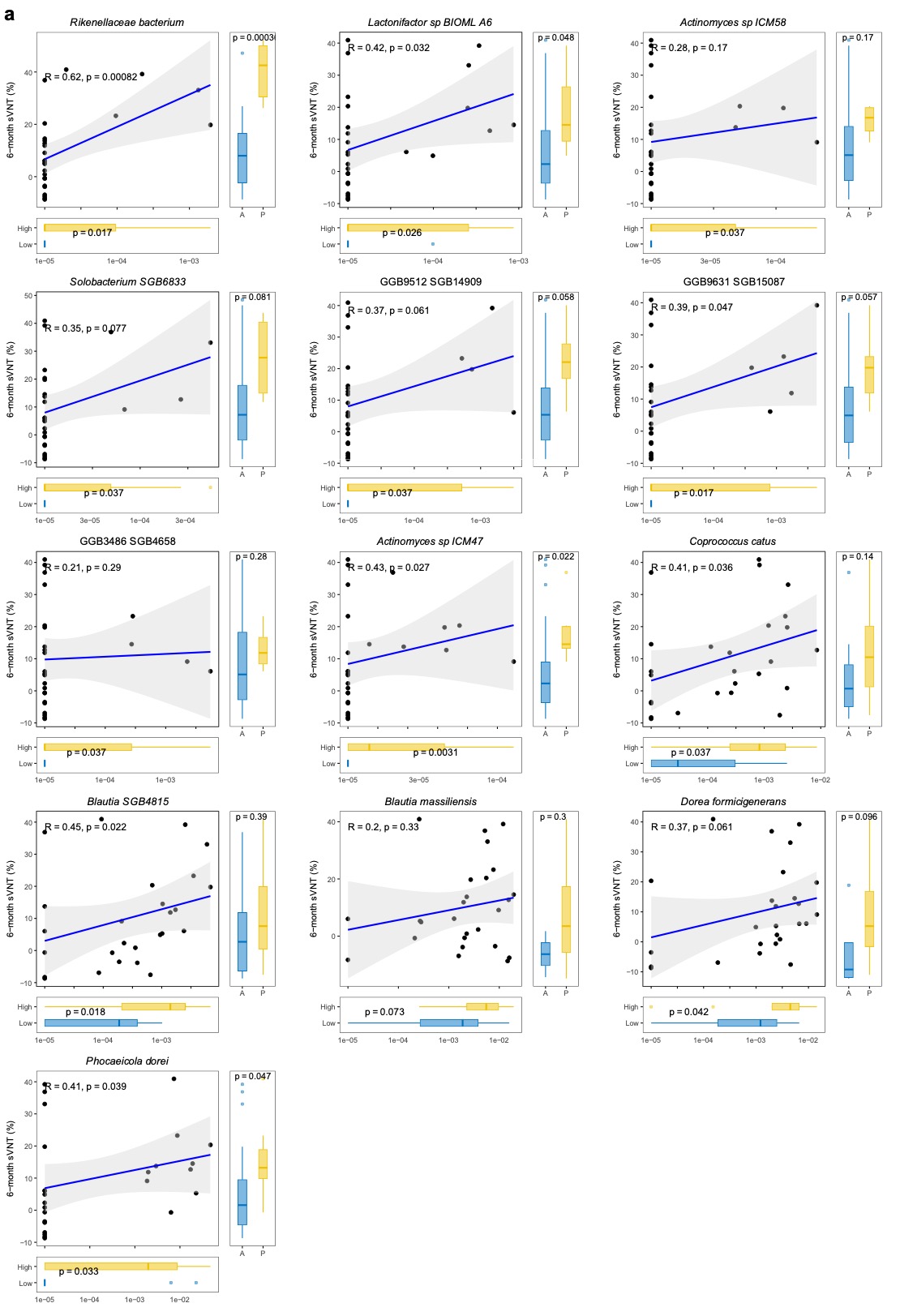
**

**
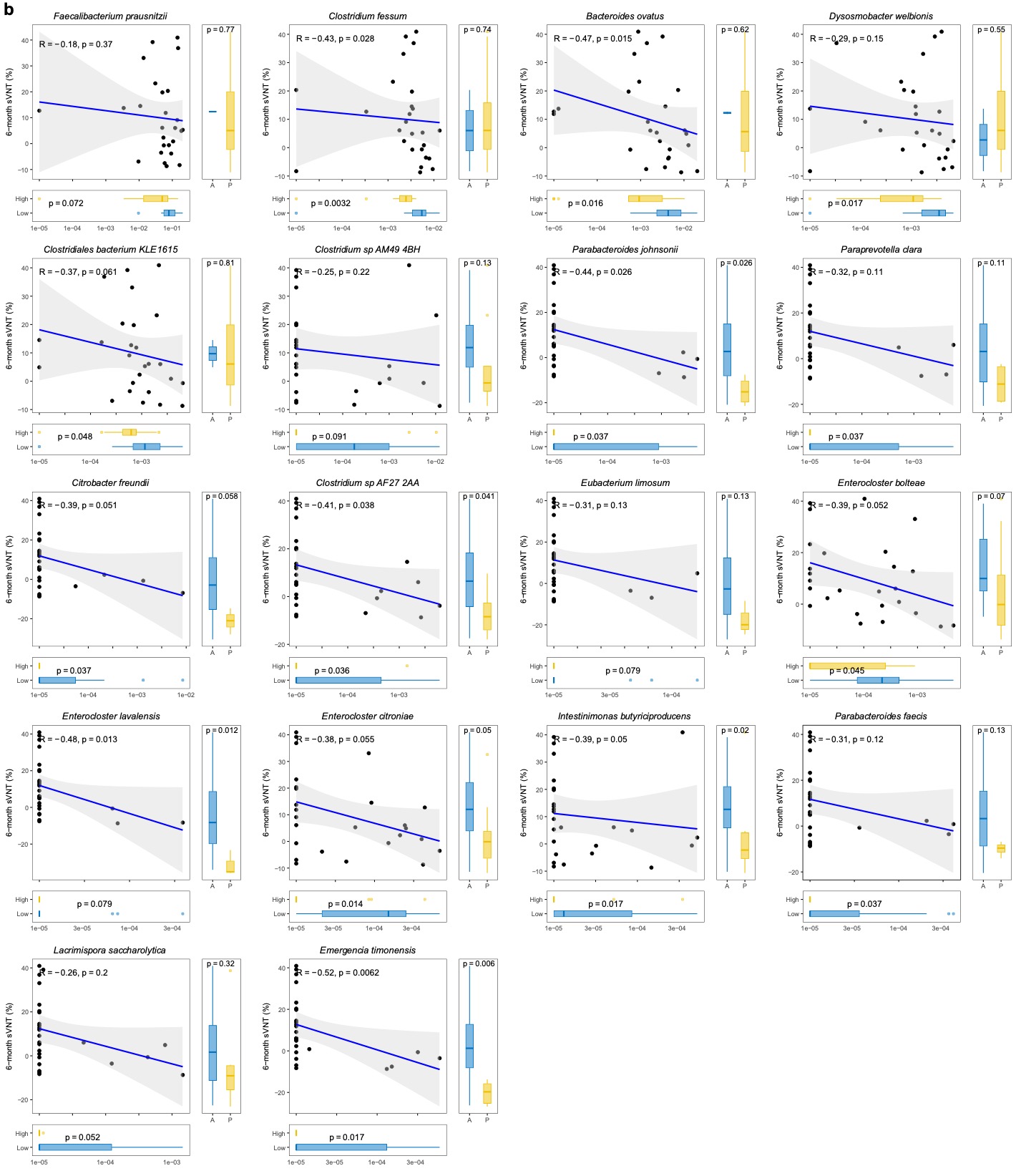
 Figure S7. Correlations between bacterial relative abundances at baseline and 6-month sVNT levels among CoronaVac vaccinees. a** Species with relative abundances positively associated with 6-month sVNT levels. **b** Species with relative abundances negatively associated with 6-month sVNT levels.

** Figure S8. Pathway enrichment of the metabolite markers for high vs. low sVNT levels to the BNT162b2 and CoronaVac vaccines.** **a** Pathways enriched for metabolite markers for high sVNT levels to BNT162b2. **b** Pathways enriched for metabolite markers for high sVNT levels to CoronaVac. **c** Pathways enriched for metabolite markers for low sVNT levels to CoronaVac. Pathways enriched in both vaccine groups were highlighted in bold.

**
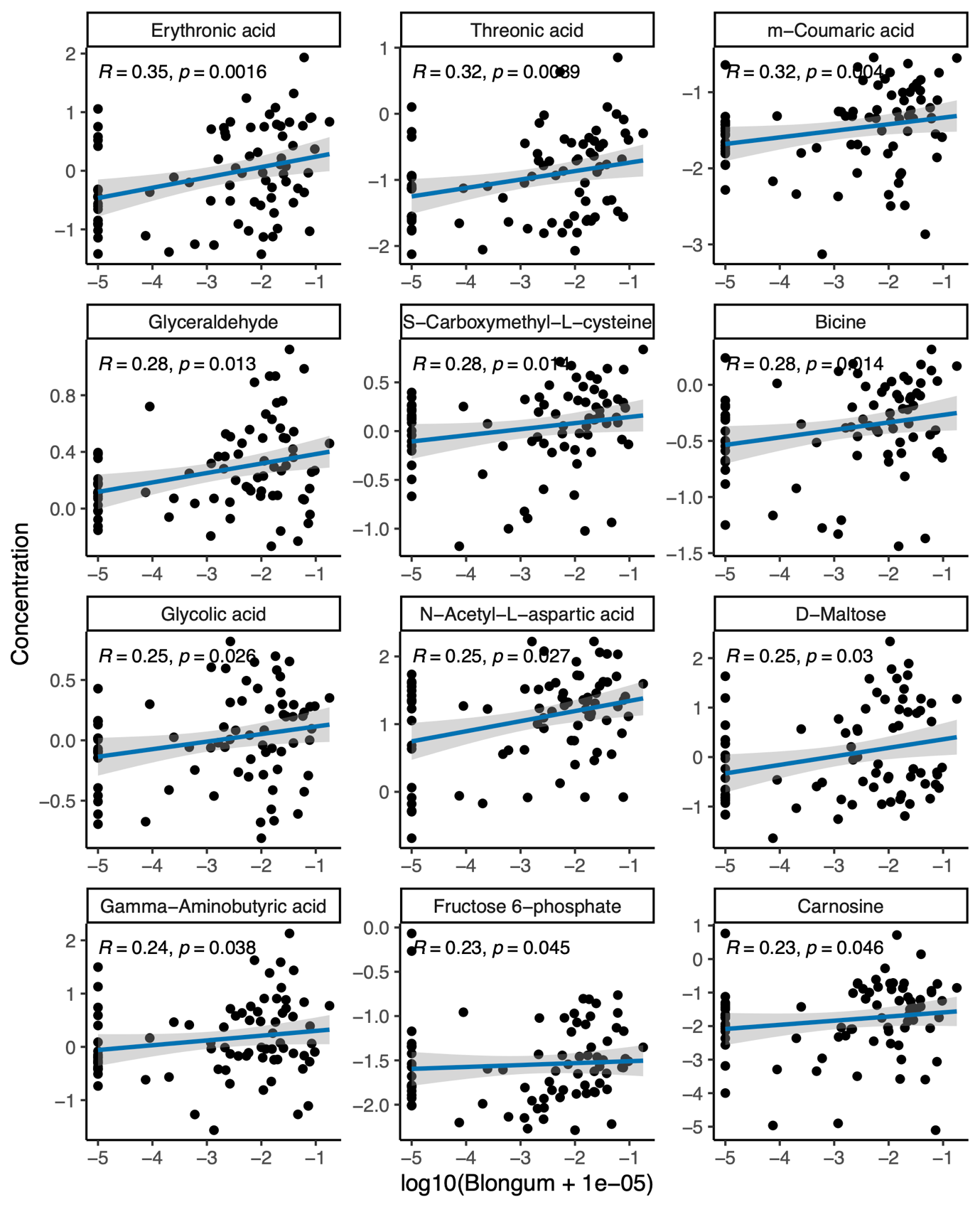
 Figure S9. Correlations between baseline relative abundance of *Bifidobacterium longum* and baseline levels of potential metabolite markers among BNT162b2 vaccinees.**


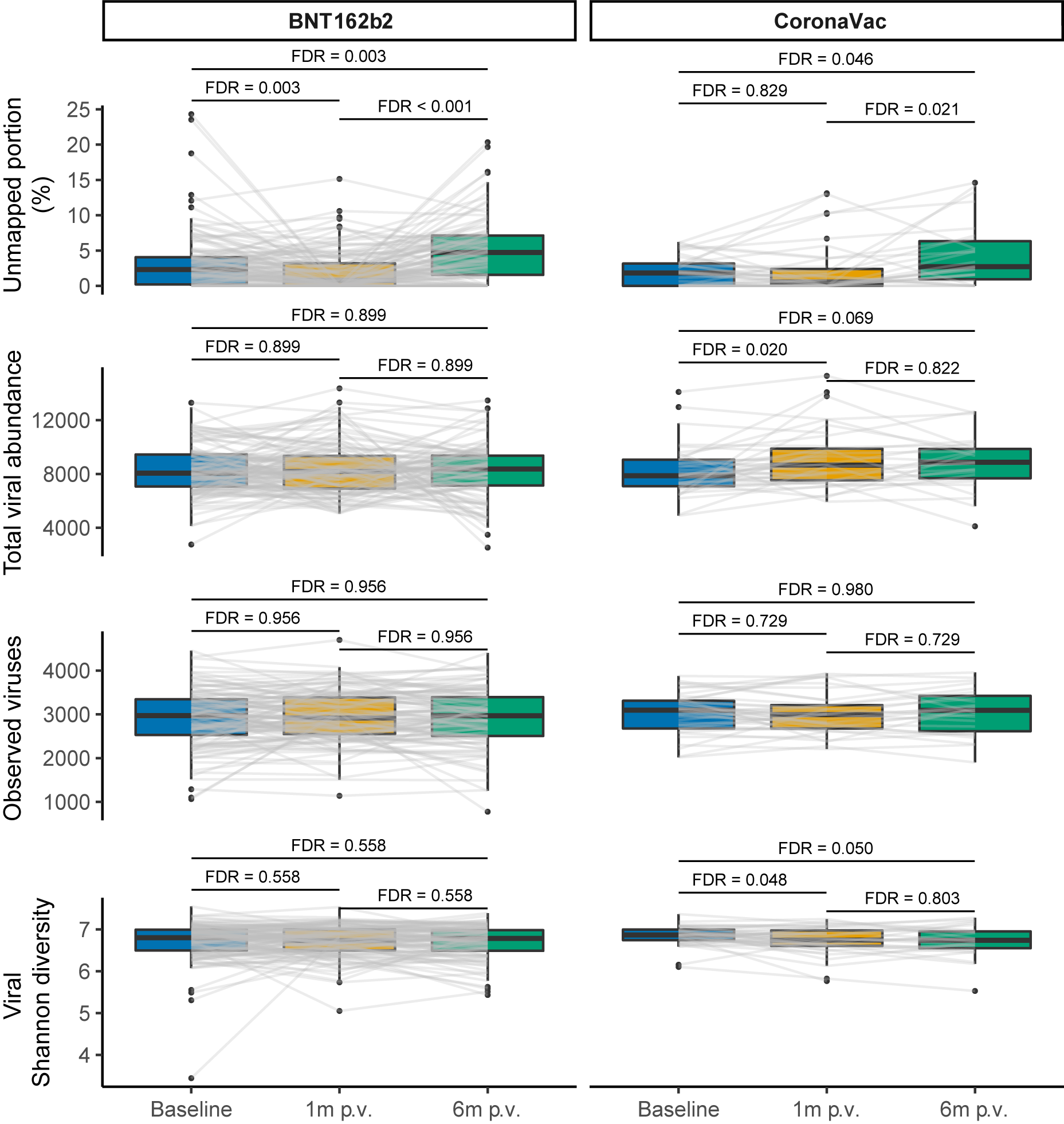
 **Figure S10. Dynamics of the proportion of reads not mapped to cellular microbiome and viral abundance and diversity.** *P* values were given by paired Wilcoxon’s rank-sum tests and were FDR-corrected. Elements on boxplots: centre line, median; box limits, upper and lower quartiles; whiskers, 1.5×IQR; points, outliers.


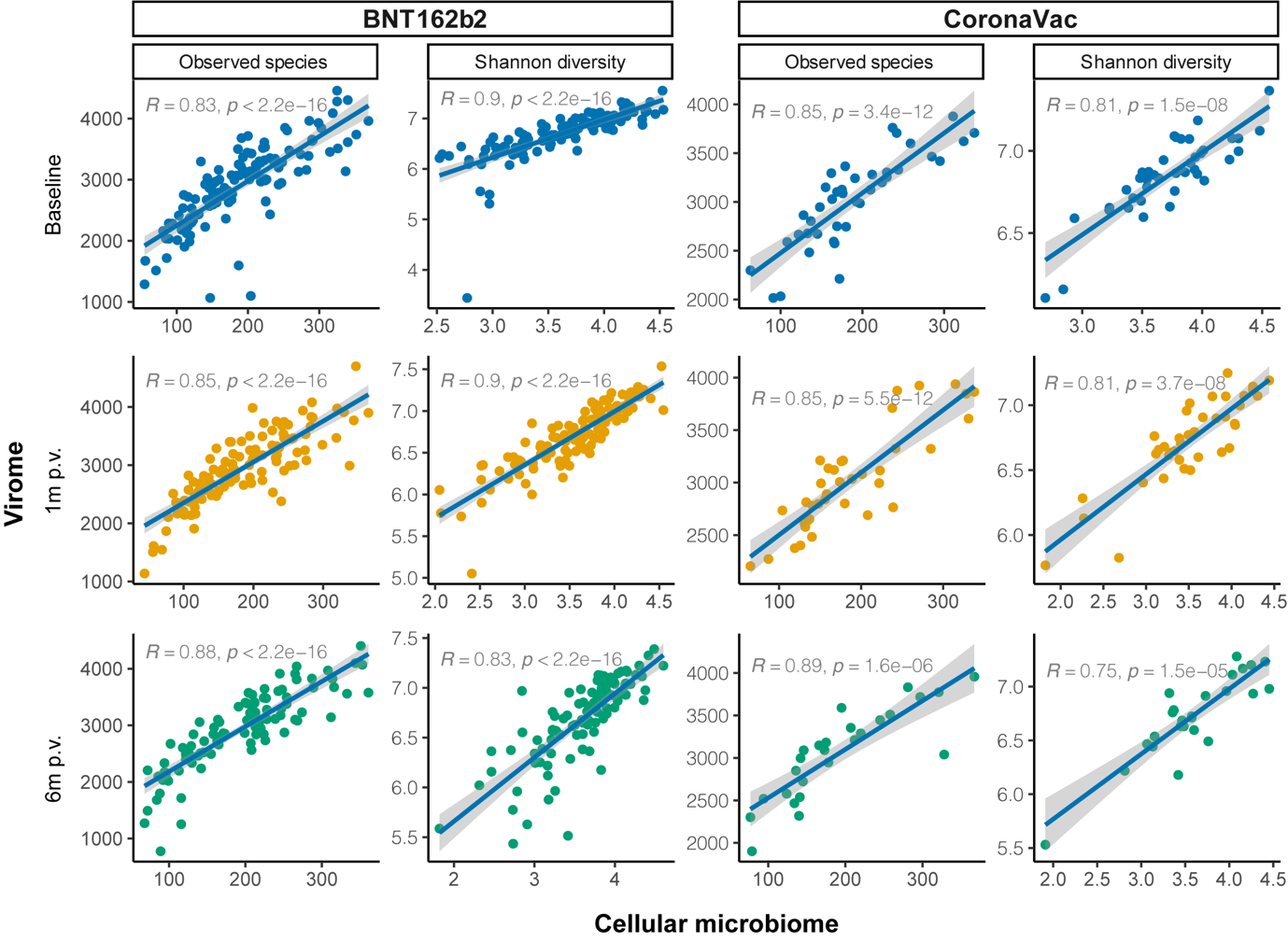
 **Figure. S11. Correlations between diversity indices of gut cellular microbiome and gut virome.** Correlation coefficients and *p* values were given by Spearman’s correlation tests.


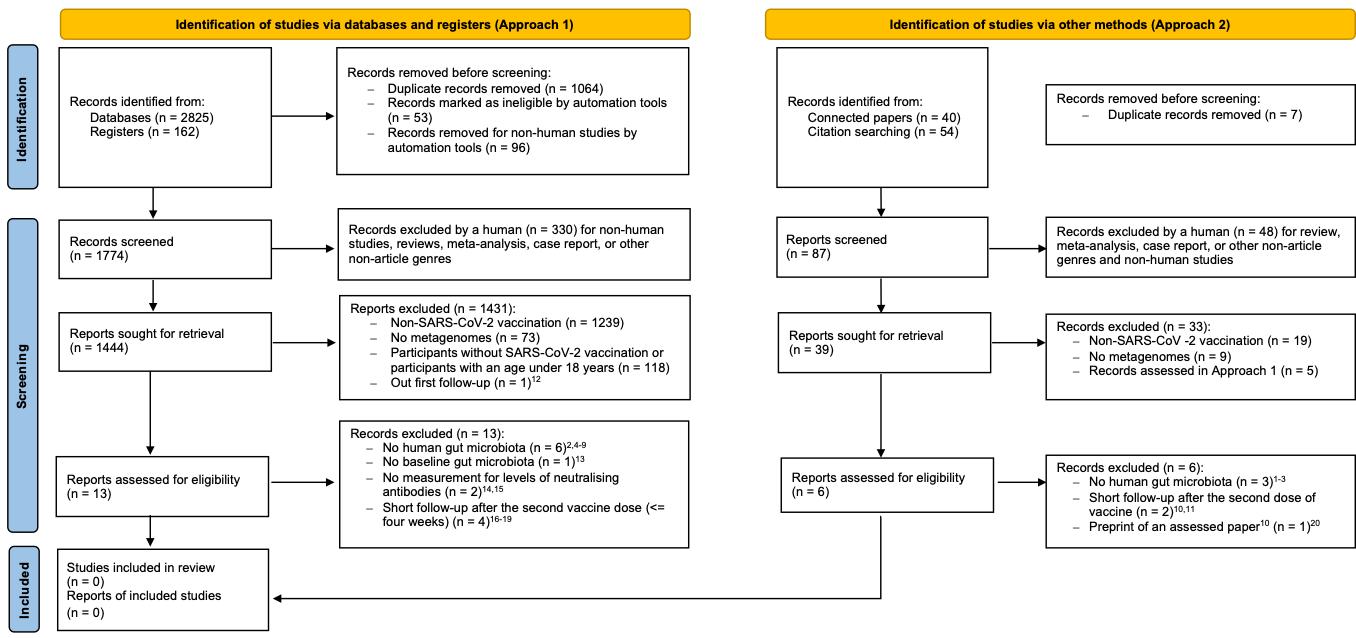


**Figure S12. PRISMA (Preferred Reporting Items for Systematic Reviews and Meta-Analyses) flow diagram for systematic search for external validation cohorts.**

**References**

1. Ghorbani M*, et al.* Persistence of salivary antibody responses after COVID-19 vaccination is associated with oral microbiome variation in both healthy and people living with HIV. *Front Immunol* **13**, 1079995 (2022).

2. Uehara O*, et al.* Alterations in the oral microbiome of individuals with a healthy oral environment following COVID-19 vaccination. *BMC Oral Health* **22**, 50 (2022).

3. Zhao S*, et al.* COVID-19 mRNA vaccine-mediated antibodies in human breast milk and their association with breast milk microbiota composition. *Research Square Preprint*, (2023).

4. Shen Y*, et al.* BBIBP-CorV Vaccination against the SARS-CoV-2 Virus Affects the Gut Microbiome. *VACCINES* **11**, (2023).

5. Rahaman MM*, et al.* Genomic characterization of the dominating Beta, V2 variant carrying vaccinated (Oxford−AstraZeneca) and nonvaccinated COVID-19 patient samples in Bangladesh: A metagenomics and whole-genome approach. *Journal of Medical Virology* **94**, 1670-1688 (2022).

6. Fernández-Ferreiro A*, et al.* Effects of Loigolactobacillus coryniformis K8 CECT 5711 on the Immune Response of Elderly Subjects to COVID-19 Vaccination: A Randomized Controlled Trial. *Nutrients* **14**, (2022).

7. Geanes ES*, et al.* Cross-reactive antibodies elicited to conserved epitopes on SARS-CoV-2 spike protein after infection and vaccination. *SCIENTIFIC REPORTS* **12**, (2022).

8. Jia L*, et al.* Preexisting antibodies targeting SARS-CoV-2 S2 cross-react with commensal gut bacteria and impact COVID-19 vaccine induced immunity. *Gut Microbes* **14**, (2022).

9. Devi P*, et al.* Transcriptionally active nasopharyngeal commensals and opportunistic microbial dynamics define mild symptoms in the COVID 19 vaccination breakthroughs. *PLoS Pathogens* **19**, (2023).

10. Lunken GR*, et al.* Gut microbiome and dietary fibre intake strongly associate with IgG function and maturation following SARS-CoV-2 mRNA vaccination. *Gut*, (2022).

11. Han M*, et al.* Dynamic changes in host immune system and gut microbiota are associated with the production of SARS-CoV-2 antibodies. *Gut*, (2022).

12. Ng SC*, et al.* Gut microbiota composition is associated with SARS-CoV-2 vaccine immunogenicity and adverse events. *Gut*, (2022).

13. Hirota M*, et al.* Human immune and gut microbial parameters associated with inter-individual variations in COVID-19 mRNA vaccine-induced immunity. *Communications Biology* **6**, (2023).

14. Wong MCS*, et al.* Effects of Gut Microbiome Modulation on Reducing Adverse Health Outcomes among Elderly and Diabetes Patients during the COVID-19 Pandemic: A Randomised, Double-Blind, Placebo-Controlled Trial (IMPACT Study). *Nutrients* **15**, (2023).

15. Jiao J*, et al.* Characterization of the Intestinal Microbiome in Healthy Adults over Sars-Cov-2 Vaccination. *FRONTIERS IN BIOSCIENCE-LANDMARK* **27**, (2022).

16. Tang B*, et al.* Correlation of gut microbiota and metabolic functions with the antibody response to the BBIBP-CorV vaccine. *Cell Rep Med* **3**, 100752 (2022).

17. Gao X*, et al.* Dendrobium officinale aqueous extract influences the immune response following vaccination against SARS-CoV-2. *Biomedicine and Pharmacotherapy* **162**, (2023).

18. Alexander JL*, et al.* The gut microbiota and metabolome are associated with diminished COVID-19 vaccine-induced antibody responses in immunosuppressed inflammatory bowel disease patients. *EBioMedicine* **88**, 104430 (2023).

19. Seong H*, et al.* Gut microbiota as a potential key to modulating humoral immunogenicity of new platform COVID-19 vaccines. *Signal Transduct Target Ther* **8**, 178 (2023).

20. Healey GR*, et al.* Variability in mRNA SARS-CoV-2 BNT162b2 vaccine immunogenicity is associated with differences in the gut microbiome and habitual dietary fibre intake. *MedRxiv preprint*.

**Supplementary Tables**

**Table S1. Correlations between baseline cytokines/chemokines and sVNT levels to the BNT162b2 and CoronaVac vaccines at 6 months p.v.**

|  |  | BNT162b2 | | |  | CoronaVac | | |
| --- | --- | --- | --- | --- | --- | --- | --- | --- |
| Panel | Target (Bead ID) | Spearman's Rho | P value | FDR |  | Spearman's Rho | P value | FDR |
| Human Proinflammatory Chemokine Panel 1 | IL-8 (A4) | 0.01 | 0.911 | 0.988 |  | -0.44 | 0.032 | 0.125 |
|  | IP-10 (A5) | -0.05 | 0.668 | 0.961 |  | -0.16 | 0.447 | 0.567 |
|  | Eotaxin (A6) | -0.06 | 0.588 | 0.961 |  | -0.66 | <0.001 | **0.005** |
|  | TARC (A7) | -0.04 | 0.736 | 0.961 |  | -0.19 | 0.38 | 0.547 |
|  | MCP-1 (A8) | -0.03 | 0.784 | 0.961 |  | -0.5 | 0.012 | **0.062** |
|  | RANTES (A10) | 0 | 0.986 | 0.988 |  | -0.28 | 0.179 | 0.379 |
|  | MIP-1α (B2) | 0.02 | 0.874 | 0.977 |  | -0.3 | 0.16 | 0.361 |
|  | MIG (B3) | -0.09 | 0.408 | 0.961 |  | -0.09 | 0.66 | 0.719 |
|  | ENA-78 (B4) | 0.06 | 0.608 | 0.961 |  | -0.22 | 0.306 | 0.501 |
|  | MIP-3α (B5) | -0.15 | 0.172 | 0.961 |  | -0.27 | 0.21 | 0.414 |
|  | GROα (B6) | -0.04 | 0.699 | 0.961 |  | -0.11 | 0.612 | 0.688 |
|  | I-TAC (B7) | 0.02 | 0.865 | 0.977 |  | -0.34 | 0.102 | 0.305 |
|  | MIP-1β (B9) | 0.09 | 0.435 | 0.961 |  | 0.01 | 0.959 | 0.97 |
| Human Th Cytokine Panel | IL-5 (A4) | 0.09 | 0.413 | 0.961 |  | -0.71 | <0.001 | **0.004** |
|  | IL-13 (A5) | 0.1 | 0.351 | 0.961 |  | -0.53 | 0.008 | **0.062** |
|  | IL-2 (A6) | -0.05 | 0.635 | 0.961 |  | -0.03 | 0.879 | 0.931 |
|  | IL-6 (A7) | 0.09 | 0.41 | 0.961 |  | -0.2 | 0.343 | 0.537 |
|  | IL-9 (A8) | 0 | 0.988 | 0.988 |  | -0.01 | 0.97 | 0.97 |
|  | IL-10 (A10) | 0.06 | 0.611 | 0.961 |  | NA | NA | NA |
|  | IFN-γ (B2) | 0.12 | 0.278 | 0.961 |  | -0.66 | <0.001 | **0.005** |
|  | TNF-α (B3) | 0.06 | 0.595 | 0.961 |  | -0.49 | 0.014 | **0.063** |
|  | IL-17A (B4) | -0.1 | 0.352 | 0.961 |  | -0.14 | 0.51 | 0.612 |
|  | IL-17F (B5) | -0.04 | 0.705 | 0.961 |  | -0.3 | 0.15 | 0.359 |
|  | IL-4 (B6) | 0.03 | 0.816 | 0.969 |  | -0.32 | 0.123 | 0.339 |
|  | IL-22 (B9) | 0.1 | 0.39 | 0.961 |  | -0.52 | 0.009 | **0.062** |
| Human Inflammation Panel 1 | IL-1β (A4) | 0.11 | 0.339 | 0.961 |  | NA | NA | NA |
|  | IFN-α2 (A5) | 0.05 | 0.624 | 0.961 |  | -0.43 | 0.035 | 0.125 |
|  | IFN-γ (A6) | 0.07 | 0.51 | 0.961 |  | -0.32 | 0.132 | 0.339 |
|  | TNF-α (A7) | 0.04 | 0.745 | 0.961 |  | -0.25 | 0.242 | 0.426 |
|  | MCP-1 (A8) | 0.01 | 0.953 | 0.988 |  | -0.51 | 0.012 | **0.062** |
|  | IL-6 (A10) | 0.07 | 0.545 | 0.961 |  | 0.17 | 0.439 | 0.567 |
|  | IL-8 (B2) | 0.08 | 0.468 | 0.961 |  | -0.25 | 0.248 | 0.426 |
|  | IL-10 (B3) | 0.24 | 0.032 | 0.961 |  | -0.26 | 0.218 | 0.414 |
|  | IL-12p70 (B4) | 0.05 | 0.651 | 0.961 |  | -0.13 | 0.559 | 0.649 |
|  | IL-17A (B5) | -0.09 | 0.425 | 0.961 |  | -0.35 | 0.097 | 0.305 |
|  | IL-18 (B6) | 0.19 | 0.089 | 0.961 |  | 0.19 | 0.364 | 0.547 |
|  | IL-23 (B7) | -0.08 | 0.494 | 0.961 |  | 0.17 | 0.428 | 0.567 |
|  | IL-33 (B9) | 0.03 | 0.775 | 0.961 |  | -0.16 | 0.457 | 0.567 |

**Table S2. Associations between demographic factors and sVNT levels to the BNT162b2 and CoronaVac vaccines at 6 months p.v.**

|  | BNT162b2 | |  | CoronaVac | |
| --- | --- | --- | --- | --- | --- |
| *Continuous variable* | Spearman's Rho | *P* value |  | Spearman's Rho | *P* value |
| Age, years | -0.1 | 0.334 |  | 0.09 | 0.65 |
| BMI, kg/m^2^ | 0.04 | 0.731 |  | 0.01 | 0.957 |
| *Categorical variable* | Median sVNT (%) 6 months p.v. (IQR) | *P* value |  | Median sVNT (%) 6 months p.v. (IQR) | *P* value |
| Sex |  | 0.207 |  |  | 0.224 |
| Female | 63.7 (51.8, 80.9) |  |  | 5.3 (-0.7, 12.7) |  |
| Male | 60.5 (44, 77.3) |  |  | 19.8 (13.7, 23.3) |  |
| Overweight or obese |  | 0.228 |  |  | 0.833 |
| No | 64.5 (53.2, 80.4) |  |  | 9.1 (-0.7, 20.3) |  |
| Yes | 59.8 (41.4, 78.2) |  |  | 5.3 (0.9, 13.7) |  |
| Hypertension |  | 0.442 |  |  | 0.429 |
| No | 63.4 (50.5, 79.2) |  |  | 5.7 (-2.8, 18.5) |  |
| Yes | 49.5 (38.2, 79.3) |  |  | 13.2 (9.7, 16.1) |  |
| Diabetes mellitus |  | 0.111 |  |  | 0.231 |
| No | 63 (48.9, 79) |  |  | 6.1 (-0.6, 19.8) |  |
| Yes | 93.5 (93.5, 93.5) |  |  | -7.6 (-7.6, -7.6) |  |
| Allergy history |  | 0.173 |  |  | 0.054 |
| No | 66 (54.8, 79.1) |  |  | 10.5 (5, 18.5) |  |
| Yes | 59.5 (40.6, 79.3) |  |  | -5.2 (-7.8, 8.7) |  |
| Diarrhea (in past 3 months prior to enrollment) |  | 0.57 |  |  | 0.534 |
| No | 62.9 (45.3, 78.3) |  |  | 5.7 (-2.8, 13.3) |  |
| Yes | 66 (49.5, 80.1) |  |  | 14.5 (0.1, 21.5) |  |
| Other comorbidities |  | 0.627 |  |  | 0.394 |
| No | 63.7 (49.8, 79.5) |  |  | 5.7 (-1.4, 15.8) |  |
| Yes | 58.5 (45, 74.3) |  |  | 21.4 (13.7, 29.2) |  |
| Antibiotic intake, past 3 months prior to enrollment |  | 0.459 |  |  |  |
| No | 62.9 (48.7, 79.5) |  |  | -- |  |
| Yes | 73.7 (57.9, 78.1) |  |  | -- |  |
| Hormone therapy |  | 0.62 |  |  |  |
| No | 63.1 (49.5, 79.3) |  |  | -- |  |
| Yes | 57.3 (35.6, 73.4) |  |  | -- |  |
| Immunomodulator |  | 0.171 |  |  | 0.231 |
| No | 63.4 (49.6, 79.6) |  |  | 6 (-0.7, 14.5) |  |
| Yes | 49.1 (26, 58.6) |  |  | 36.9 (36.9, 36.9) |  |
| Probiotics |  | 0.772 |  |  | **0.048** |
| No | 64.5 (48.6, 79.2) |  |  | 7.6 (1.2, 20.2) |  |
| Yes | 59.5 (50.8, 80.4) |  |  | -4.5 (-8.4, 2.4) |  |
| Vaccination in the past year |  | **0.009** |  |  | **0.017** |
| No | 68.9 (59.1, 80.6) |  |  | 3.6 (-3.6, 9.8) |  |
| Yes | 56.8 (39.4, 76) |  |  | 20.1 (7.8, 33.5) |  |
| Dietary habit |  | 0.424 |  |  |  |
| Vegetarian | 53 (49.3, 56.8) |  |  | -- |  |
| Normal | 63.4 (49.2, 79.2) |  |  | -- |  |
| Alcohol intake (within 2 weeks prior to first vaccine dose) |  | 0.672 |  |  | 0.706 |
| No | 63.7 (46, 80.3) |  |  | 7.6 (-0.7, 20.2) |  |
| Yes | 61.6 (51.8, 73.5) |  |  | 4.2 (0.9, 8.2) |  |
| Regular exercise (strenuous/moderate) |  | 0.503 |  |  | **0.017** |
| No | 61.2 (44.6, 80.6) |  |  | -2.1 (-6.6, 5.2) |  |
| Yes | 64.5 (52.6, 78.9) |  |  | 13.2 (5.2, 21.1) |  |
| Antibiotics intake, 3 months to 6 months p.v. |  | 0.742 |  |  | 0.75 |
| No | 63.7 (48.7, 80.3) |  |  | 6.1 (-0.6, 17.1) |  |
| Yes | 75.2 (63.6, 77.1) |  |  | 2.3 (2.3, 2.3) |  |

**Table S3. Multivariable generalised linear regression modeling of demographic factors potentially associated with sVNT levels to the CoronaVac vaccine at 6 months p.v.**

| Variable | Coefficient (95% CI) | *P* value |
| --- | --- | --- |
| Intercept | 2.41 (-6.14, 10.97) | 0.586 |
| Vaccination in the past year | 11.55 (1.12, 21.99) | 0.041 |
| Regular exercise | 7.82 (-2.6, 18.24) | 0.155 |
| Probiotics | -10.67 (-24.16, 2.82) | 0.135 |

**Table S4. Relative abundances of major bacterial phyla from baseline to 6 months post vaccination.**

|  |  | **Median (IQR) relative abundance (%)** | | |  | **Wilcoxon's *p*** | |  | **FDR-corrected *p* value** | |
| --- | --- | --- | --- | --- | --- | --- | --- | --- | --- | --- |
|  |  | Baseline | 1 month p.v. | 6 months p.v. |  | Baseline vs. 1 month p.v. | Baseline vs. 6 months p.v. |  | Baseline vs. 1 month p.v. | Baseline vs. 6 months p.v. |
| **BNT162b2** | Bacillota | 62.6 (50.1, 75.6) | 62.4 (45.3, 70.4) | 57.1 (48.1, 68.4) |  | 0.005 | 0.002 |  | 0.011 | 0.007 |
|  | Bacteroidota | 24.6 (12.8, 39.3) | 28 (18.3, 42.5) | 32.5 (22.1, 42.6) |  | 0.008 | <0.001 |  | 0.013 | 0.002 |
|  | Actinomycetota | 3.7 (2.2, 8.6) | 2.9 (1.2, 6) | 2.8 (1.4, 5.7) |  | 0.012 | 0.008 |  | 0.017 | 0.013 |
|  | Pseudomonadota | 2.2 (1.5, 3.3) | 2.8 (1.7, 5.4) | 2.5 (1.6, 3.8) |  | <0.001 | 0.019 |  | 0.001 | 0.023 |
|  | Verrucomicrobiota | 0 (0, 0.1) | 0 (0, 0.1) | 0 (0, 0.1) |  | 0.873 | 0.992 |  | 0.970 | 0.992 |
| **CoronaVac** | Bacillota | 68.7 (52.5, 76.7) | 52.6 (38.1, 67.8) | 55.8 (44.8, 66.3) |  | <0.001 | <0.001 |  | 0.001 | 0.001 |
|  | Bacteroidota | 22.2 (15.2, 36.8) | 39.7 (27.4, 52.3) | 35.6 (25.9, 45.7) |  | <0.001 | <0.001 |  | 0.001 | 0.001 |
|  | Actinomycetota | 3.2 (1.6, 5.8) | 1.3 (0.7, 3.3) | 1.3 (1.1, 3) |  | 0.010 | 0.004 |  | 0.015 | 0.007 |
|  | Pseudomonadota | 1.5 (1, 2.5) | 2.7 (2, 3.7) | 2.4 (1.8, 3.9) |  | 0.002 | 0.038 |  | 0.004 | 0.048 |
|  | Verrucomicrobiota | 0 (0, 0) | 0 (0, 0) | 0 (0, 0.1) |  | 0.478 | 0.230 |  | 0.478 | 0.256 |

FDR correction was performed across all comparisons within one vaccine group.

**Table S5.** **Correlations between baseline relative abundances of potential species markers and 6-month sVNT levles in the BNT162b2 group.**

|  | Species | N (%) | Spearman's Rho | P value | FDR |
| --- | --- | --- | --- | --- | --- |
| BNT162b2 markers | s__Lactococcus_lactis | 17 (16.8) | 0.27 | 0.006 | 0.07 |
|  | s__Bifidobacterium_adolescentis | 73 (72.3) | 0.26 | 0.009 | 0.07 |
|  | s__Clostridiaceae_bacterium_NSJ_33 | 4 (4) | -0.24 | 0.015 | 0.07 |
|  | s__GGB4596_SGB6358 | 8 (7.9) | 0.23 | 0.018 | 0.07 |
|  | s__Roseburia_sp_AM23_20 | 9 (8.9) | -0.22 | 0.024 | 0.07 |
|  | s__Latilactobacillus_sakei | 3 (3) | -0.22 | 0.025 | 0.07 |
|  | s__Parasutterella_excrementihominis | 47 (46.5) | 0.22 | 0.027 | 0.07 |
|  | s__Fusobacterium_ulcerans | 4 (4) | 0.22 | 0.03 | 0.07 |
|  | s__Roseburia_intestinalis | 46 (45.5) | 0.2 | 0.05 | 0.106 |
|  | s__Lachnospira_pectinoschiza | 44 (43.6) | 0.18 | 0.077 | 0.146 |
|  | s__Candidatus_Nanosynsacchari_sp_TM7_ANC_38_39_G1_1 | 5 (5) | -0.16 | 0.119 | 0.205 |
|  | s__Bifidobacterium_bifidum | 16 (15.8) | 0.15 | 0.138 | 0.219 |
|  | s__Alistipes_dispar | 14 (13.9) | -0.13 | 0.18 | 0.246 |
|  | s__GGB3738_SGB5072 | 12 (11.9) | 0.13 | 0.181 | 0.246 |
|  | s__Clostridia_unclassified_SGB71281 | 4 (4) | 0.13 | 0.196 | 0.248 |
|  | s__Roseburia_faecis | 75 (74.3) | 0.09 | 0.346 | 0.381 |
|  | s__Candidatus_Heteroscilispira_lomanii | 3 (3) | -0.09 | 0.357 | 0.381 |
|  | s__Candidatus_Metaruminococcus_caecorum | 4 (4) | -0.09 | 0.361 | 0.381 |
|  | s__Candidatus_Allobutyricicoccus_pentlandensis | 4 (4) | -0.09 | 0.386 | 0.386 |
| CoronaVac markers | s__Rikenellaceae_bacterium | 5 (19.2) | 0.62 | 0.001 | 0.025 |
|  | s__Emergencia_timonensis | 5 (19.2) | -0.52 | 0.006 | 0.095 |
|  | s__Enterocloster_lavalensis | 3 (11.5) | -0.48 | 0.013 | 0.095 |
|  | s__Bacteroides_ovatus | 24 (92.3) | -0.47 | 0.015 | 0.095 |
|  | s__Blautia_SGB4815 | 20 (76.9) | 0.45 | 0.022 | 0.095 |
|  | s__Parabacteroides_johnsonii | 4 (15.4) | -0.44 | 0.026 | 0.095 |
|  | s__Actinomyces_sp_ICM47 | 7 (26.9) | 0.43 | 0.027 | 0.095 |
|  | s__Clostridium_fessum | 24 (92.3) | -0.43 | 0.028 | 0.095 |
|  | s__Lactonifactor_sp_BIOML_A6 | 7 (26.9) | 0.42 | 0.032 | 0.095 |
|  | s__Coprococcus_catus | 18 (69.2) | 0.41 | 0.036 | 0.095 |
|  | s__Clostridium_sp_AF27_2AA | 7 (26.9) | -0.41 | 0.038 | 0.095 |
|  | s__Phocaeicola_dorei | 10 (38.5) | 0.41 | 0.039 | 0.095 |
|  | s__GGB9631_SGB15087 | 5 (19.2) | 0.39 | 0.047 | 0.095 |
|  | s__Intestinimonas_butyriciproducens | 11 (42.3) | -0.39 | 0.05 | 0.095 |
|  | s__Citrobacter_freundii | 4 (15.4) | -0.39 | 0.051 | 0.095 |
|  | s__Enterocloster_bolteae | 18 (69.2) | -0.39 | 0.052 | 0.095 |
|  | s__Enterocloster_citroniae | 13 (50) | -0.38 | 0.055 | 0.095 |
|  | s__Clostridiales_bacterium_KLE1615 | 24 (92.3) | -0.37 | 0.061 | 0.095 |
|  | s__Dorea_formicigenerans | 22 (84.6) | 0.37 | 0.061 | 0.095 |
|  | s__GGB9512_SGB14909 | 4 (15.4) | 0.37 | 0.061 | 0.095 |
|  | s__Solobacterium_SGB6833 | 4 (15.4) | 0.35 | 0.077 | 0.113 |
|  | s__Paraprevotella_clara | 4 (15.4) | -0.32 | 0.109 | 0.154 |
|  | s__Parabacteroides_faecis | 4 (15.4) | -0.31 | 0.123 | 0.166 |
|  | s__Eubacterium_limosum | 3 (11.5) | -0.31 | 0.128 | 0.166 |
|  | s__Dysosmobacter_welbionis | 24 (92.3) | -0.29 | 0.145 | 0.18 |
|  | s__Actinomyces_sp_ICM58 | 4 (15.4) | 0.28 | 0.17 | 0.202 |
|  | s__Lacrimispora_saccharolytica | 6 (23.1) | -0.26 | 0.196 | 0.225 |
|  | s__Clostridium_sp_AM49_4BH | 9 (34.6) | -0.25 | 0.218 | 0.241 |
|  | s__GGB3486_SGB4658 | 4 (15.4) | 0.21 | 0.293 | 0.313 |
|  | s__Blautia_massiliensis | 24 (92.3) | 0.2 | 0.335 | 0.346 |
|  | s__Faecalibacterium_prausnitzii | 25 (96.2) | -0.18 | 0.373 | 0.373 |

**Table S6. Correlations between age and baseline relative abundances of potential species markers.**

| **BNT162b2** | | |  | **CoronaVac** | | |
| --- | --- | --- | --- | --- | --- | --- |
| Feature | Spearman's Rho | *P* value |  | Feature | Spearman's Rho | *P* value |
| s__Candidatus_Heteroscilispira_lomanii | -0.18 | **0.044** |  | s__Parabacteroides_faecis | -0.25 | 0.113 |
| s__Roseburia_sp_AM23_20 | -0.07 | 0.437 |  | s__Intestinimonas_butyriciproducens | -0.08 | 0.613 |
| s__Candidatus_Allobutyricicoccus_pentlandensis | -0.13 | 0.157 |  | s__Faecalibacterium_prausnitzii | 0.03 | 0.833 |
| s__Bifidobacterium_adolescentis | 0.03 | 0.756 |  | s__Enterocloster_bolteae | 0.01 | 0.967 |
| s__Roseburia_intestinalis | 0.11 | 0.211 |  | s__GGB3486_SGB4658 | -0.11 | 0.486 |
| s__Candidatus_Nanosynsacchari_sp_TM7_ANC_38_39_G1_1 | -0.03 | 0.729 |  | s__Paraprevotella_clara | 0.13 | 0.411 |
| s__GGB3738_SGB5072 | 0.10 | 0.270 |  | s__Enterocloster_lavalensis | 0.07 | 0.666 |
| s__Parasutterella_excrementihominis | -0.12 | 0.194 |  | s__Actinomyces_sp_ICM47 | 0.01 | 0.930 |
| s__Latilactobacillus_sakei | 0.01 | 0.948 |  | s__Clostridium_sp_AF27_2AA | 0.02 | 0.886 |
| s__Clostridiaceae_bacterium_NSJ_33 | 0.03 | 0.747 |  | s__Coprococcus_catus | 0.05 | 0.776 |
| s__Candidatus_Metaruminococcus_caecorum | 0.04 | 0.681 |  | s__GGB9512_SGB14909 | 0.01 | 0.942 |
| s__Roseburia_faecis | -0.04 | 0.636 |  | s__Blautia_SGB4815 | 0.04 | 0.821 |
| s__Bifidobacterium_bifidum | -0.16 | 0.083 |  | s__Blautia_massiliensis | 0.03 | 0.841 |
| s__Fusobacterium_ulcerans | -0.08 | 0.383 |  | s__Clostridium_fessum | -0.02 | 0.887 |
| s__Lachnospira_pectinoschiza | 0.01 | 0.893 |  | s__Dorea_formicigenerans | 0.04 | 0.789 |
| s__Alistipes_dispar | -0.05 | 0.576 |  | s__Dysosmobacter_welbionis | -0.05 | 0.737 |
| s__Clostridia_unclassified_SGB71281 | -0.09 | 0.332 |  | s__Actinomyces_sp_ICM58 | -0.16 | 0.320 |
| s__GGB4596_SGB6358 | 0.15 | 0.090 |  | s__Lacrimispora_saccharolytica | 0.12 | 0.457 |
| s__Lactococcus_lactis | -0.17 | 0.056 |  | s__Phocaeicola_dorei | 0.06 | 0.710 |
|  |  |  |  | s__Enterocloster_citroniae | -0.07 | 0.687 |
|  |  |  |  | s__Eubacterium_limosum | -0.22 | 0.173 |
|  |  |  |  | s__Bacteroides_ovatus | -0.21 | 0.199 |
|  |  |  |  | s__Clostridium_sp_AM49_4BH | 0.23 | 0.160 |
|  |  |  |  | s__GGB9631_SGB15087 | 0.05 | 0.778 |
|  |  |  |  | s__Rikenellaceae_bacterium | 0.22 | 0.182 |
|  |  |  |  | s__Emergencia_timonensis | -0.05 | 0.753 |
|  |  |  |  | s__Parabacteroides_johnsonii | -0.19 | 0.240 |
|  |  |  |  | s__Citrobacter_freundii | -0.40 | **0.011** |
|  |  |  |  | s__Clostridiales_bacterium_KLE1615 | 0.27 | 0.090 |
|  |  |  |  | s__Lactonifactor_sp_BIOML_A6 | 0.03 | 0.865 |
|  |  |  |  | s__Solobacterium_SGB6833 | -0.19 | 0.236 |

**Table S7. Validation of baseline species markers for 6-month sVNT levels in the BNT162b2 group.**

|  | ALDEx2 | | | | |  | LINDA | | | |  | LEfSe | | |
| --- | --- | --- | --- | --- | --- | --- | --- | --- | --- | --- | --- | --- | --- | --- |
| Potential species markers | Wilcoxon p value | FDR | Diff. between groups | Diff. within groups | Effect |  | log2FoldChange | lfcSE | P value | FDR |  | Enrichment group | logLDA | P value |
| s__Bifidobacterium_adolescentis | **0.004** | 0.814 | 2.75 | 17.24 | 0.20 |  | 4.16 | 1.66 | **0.014** | 1.000 |  | High | 3.74 | 0.003 |
| s__Lachnospira_pectinoschiza | **0.027** | 0.895 | 2.78 | 19.24 | 0.20 |  | 5.23 | 1.84 | **0.005** | 1.000 |  | High | 3.35 | 0.014 |
| s__Roseburia_intestinalis | **0.040** | 0.931 | 2.69 | 18.00 | 0.19 |  | 4.26 | 1.77 | **0.018** | 1.000 |  | High | 3.15 | 0.030 |
| s__Parasutterella_excrementihominis | 0.052 | 0.932 | 2.80 | 14.57 | 0.20 |  | 3.17 | 1.48 | **0.034** | 1.000 |  | High | 2.57 | 0.033 |
| s__Roseburia_faecis | 0.065 | 0.956 | 1.84 | 16.95 | 0.13 |  | 3.34 | 1.67 | **0.048** | 1.000 |  | High | 3.56 | 0.036 |
| s__Lactococcus_lactis | 0.090 | 0.901 | 1.81 | 9.75 | 0.19 |  | 3.22 | 0.95 | **0.001** | 1.000 |  | High | 2.90 | 0.001 |
| s__GGB4596_SGB6358 | 0.245 | 0.956 | 0.97 | 5.78 | 0.12 |  | 1.70 | 0.75 | **0.027** | 1.000 |  | High | 2.67 | 0.031 |
| s__Bifidobacterium_bifidum | 0.246 | 0.954 | 1.19 | 9.37 | 0.11 |  | 2.43 | 1.23 | 0.050 | 1.000 |  | High | 2.77 | 0.039 |
| s__GGB3738_SGB5072 | 0.274 | 0.958 | 0.97 | 7.40 | 0.10 |  | 2.03 | 0.95 | **0.035** | 1.000 |  | High | 2.27 | 0.036 |
| s__Fusobacterium_ulcerans | 0.427 | 0.968 | 0.45 | 5.23 | 0.06 |  | 1.33 | 0.65 | **0.042** | 1.000 |  | High | 2.08 | 0.024 |
| s__Clostridia_unclassified_SGB71281 | 0.438 | 0.970 | 0.46 | 4.97 | 0.07 |  | 0.81 | 0.40 | **0.044** | 1.000 |  | High | 2.35 | 0.044 |
| s__Alistipes_dispar | 0.152 | 0.926 | -1.29 | 8.25 | -0.14 |  | -3.13 | 1.05 | **0.004** | 1.000 |  | Low | 2.60 | 0.004 |
| s__Roseburia_sp_AM23_20 | 0.369 | 0.968 | -0.69 | 6.71 | -0.07 |  | -1.90 | 1.00 | 0.061 | 1.000 |  | Low | 3.33 | 0.044 |
| s__Candidatus_Metaruminococcus_caecorum | 0.372 | 0.966 | -0.63 | 5.39 | -0.10 |  | -0.87 | 0.42 | **0.043** | 1.000 |  | Low | 2.41 | 0.048 |
| s__Candidatus_Heteroscilispira_lomanii | 0.402 | 0.966 | -0.47 | 4.92 | -0.08 |  | -0.66 | 0.30 | **0.029** | 1.000 |  | Low | 2.59 | 0.040 |
| s__Candidatus_Nanosynsacchari_sp_TM7_ANC_38_39_G1_1 | 0.411 | 0.970 | -0.52 | 5.22 | -0.09 |  | -0.82 | 0.40 | **0.045** | 1.000 |  | Low | 2.33 | 0.021 |
| s__Clostridiaceae_bacterium_NSJ_33 | 0.423 | 0.970 | -0.55 | 5.05 | -0.09 |  | -0.84 | 0.39 | **0.035** | 1.000 |  | Low | 2.31 | 0.040 |
| s__Candidatus_Allobutyricicoccus_pentlandensis | 0.426 | 0.972 | -0.46 | 5.07 | -0.07 |  | -0.76 | 0.34 | **0.026** | 1.000 |  | Low | 2.39 | 0.040 |
| s__Latilactobacillus_sakei | 0.466 | 0.973 | -0.35 | 5.03 | -0.06 |  | -0.63 | 0.32 | 0.051 | 1.000 |  | Low | 2.35 | 0.040 |
| s__Anaerotruncus_colihominis | 0.057 | 0.927 | -2.14 | 13.18 | -0.19 |  | -3.01 | 1.27 | **0.020** | 1.000 |  |  |  |  |
| s__GGB9707_SGB15229 | 0.140 | 0.949 | 1.50 | 12.83 | 0.13 |  | 2.53 | 1.20 | **0.039** | 1.000 |  |  |  |  |
| s__Intestinimonas_massiliensis | 0.173 | 0.945 | -1.34 | 8.24 | -0.15 |  | -2.35 | 0.85 | **0.007** | 1.000 |  |  |  |  |
| s__Enterobacter_hormaechei | 0.212 | 0.959 | 1.30 | 9.89 | 0.13 |  | 2.15 | 1.00 | **0.035** | 1.000 |  |  |  |  |
| s__Dielma_fastidiosa | 0.236 | 0.952 | -1.13 | 7.42 | -0.13 |  | -2.02 | 0.79 | **0.012** | 1.000 |  |  |  |  |
| s__GGB9618_SGB15065 | 0.279 | 0.945 | 0.90 | 5.93 | 0.12 |  | 1.62 | 0.69 | **0.021** | 1.000 |  |  |  |  |
| s__Eisenbergiella_tayi | 0.289 | 0.963 | -1.03 | 8.29 | -0.11 |  | -2.08 | 1.00 | **0.039** | 1.000 |  |  |  |  |
| s__GGB3005_SGB3996 | 0.306 | 0.964 | -0.92 | 8.76 | -0.10 |  | -1.75 | 0.86 | **0.045** | 1.000 |  |  |  |  |
| s__Intestinibacter_SGB6139 | 0.344 | 0.966 | 0.70 | 5.68 | 0.09 |  | 1.36 | 0.59 | **0.023** | 1.000 |  |  |  |  |
| s__Enorma_massiliensis | 0.402 | 0.968 | 0.46 | 5.23 | 0.08 |  | 1.17 | 0.53 | **0.031** | 1.000 |  |  |  |  |
| s__Eisenbergiella_massiliensis | 0.403 | 0.971 | -0.72 | 8.21 | -0.08 |  | -2.03 | 0.98 | **0.041** | 1.000 |  |  |  |  |
| s__GGB9710_SGB15239 | 0.435 | 0.972 | 0.40 | 5.10 | 0.07 |  | 0.89 | 0.42 | **0.035** | 1.000 |  |  |  |  |
| s__Clostridiales_bacterium_UBA1390 | 0.447 | 0.970 | -0.53 | 5.71 | -0.08 |  | -0.94 | 0.46 | **0.045** | 1.000 |  |  |  |  |

**Table S8. Validation of baseline species markers for 6-month sVNT levels in the CoronaVac group.**

|  | ALDEx2 | | | | |  | LINDA | | | |  | LEfSe | | |
| --- | --- | --- | --- | --- | --- | --- | --- | --- | --- | --- | --- | --- | --- | --- |
| Potential species markers | Wilcoxon p value | FDR | Diff. between groups | Diff. within groups | Effect |  | log2FoldChange | lfcSE | P value | FDR |  | Enrichment group | logLDA | P value |
| s__Actinomyces_sp_ICM47 | **0.018** | 0.724 | 9.78 | 8.19 | 0.95 |  | 5.58 | 1.64 | **0.002** | 0.501 |  | High | 2.83 | 0.001 |
| s__Blautia_SGB4815 | **0.025** | 0.844 | 2.72 | 11.63 | 0.30 |  | 2.91 | 2.35 | 0.227 | 0.726 |  | High | 3.02 | 0.009 |
| s__Coprococcus_catus | 0.067 | 0.889 | 4.26 | 12.94 | 0.42 |  | 4.52 | 2.58 | 0.092 | 0.501 |  | High | 2.87 | 0.047 |
| s__Rikenellaceae_bacterium | 0.085 | 0.840 | 4.10 | 8.72 | 0.52 |  | 4.26 | 1.74 | **0.022** | 0.501 |  | High | 2.33 | 0.027 |
| s__Phocaeicola_dorei | 0.088 | 0.873 | 15.46 | 18.36 | 0.77 |  | 7.94 | 3.50 | **0.033** | 0.501 |  | High | 3.56 | 0.046 |
| s__GGB9631_SGB15087 | 0.091 | 0.834 | 4.77 | 10.20 | 0.61 |  | 5.04 | 2.01 | **0.019** | 0.501 |  | High | 2.61 | 0.011 |
| s__Lactonifactor_sp_BIOML_A6 | 0.103 | 0.872 | 4.10 | 12.18 | 0.44 |  | 4.24 | 2.36 | 0.085 | 0.501 |  | High | 2.41 | 0.036 |
| s__Dorea_formicigenerans | 0.119 | 0.910 | 1.69 | 6.70 | 0.19 |  | 2.07 | 2.27 | 0.370 | 0.907 |  | High | 3.34 | 0.019 |
| s__Blautia_massiliensis | 0.171 | 0.925 | 1.97 | 4.50 | 0.32 |  | 2.70 | 1.80 | 0.148 | 0.575 |  | High | 3.30 | 0.034 |
| s__GGB9512_SGB14909 | 0.182 | 0.887 | 2.89 | 8.07 | 0.39 |  | 3.87 | 1.86 | **0.049** | 0.501 |  | High | 2.53 | 0.027 |
| s__Actinomyces_sp_ICM58 | 0.230 | 0.903 | 3.54 | 9.70 | 0.50 |  | 3.48 | 1.76 | 0.059 | 0.501 |  | High | 2.45 | 0.011 |
| s__GGB3486_SGB4658 | 0.231 | 0.909 | 2.79 | 8.25 | 0.30 |  | 4.10 | 2.08 | 0.060 | 0.501 |  | High | 2.68 | 0.027 |
| s__Solobacterium_SGB6833 | 0.248 | 0.900 | 2.56 | 8.34 | 0.31 |  | 2.98 | 1.69 | 0.090 | 0.501 |  | High | 2.46 | 0.027 |
| s__Enterocloster_citroniae | **0.010** | 0.794 | -6.31 | 9.23 | -0.73 |  | -7.06 | 2.07 | **0.002** | 0.501 |  | Low | 2.33 | 0.016 |
| s__Intestinimonas_butyriciproducens | **0.011** | 0.750 | -9.97 | 8.42 | -0.99 |  | -7.60 | 1.92 | **0.001** | 0.435 |  | Low | 2.31 | 0.002 |
| s__Dysosmobacter_welbionis | **0.013** | 0.812 | -2.01 | 3.09 | -0.49 |  | -2.10 | 1.38 | 0.140 | 0.558 |  | Low | 3.03 | 0.009 |
| s__Bacteroides_ovatus | **0.023** | 0.846 | -2.64 | 4.35 | -0.49 |  | -4.56 | 1.60 | **0.009** | 0.501 |  | Low | 3.18 | 0.022 |
| s__Enterocloster_bolteae | 0.062 | 0.886 | -8.66 | 12.05 | -0.70 |  | -7.68 | 2.60 | **0.007** | 0.501 |  | Low | 2.45 | 0.038 |
| s__Clostridiales_bacterium_KLE1615 | 0.065 | 0.885 | -1.56 | 3.07 | -0.24 |  | -2.15 | 1.79 | 0.241 | 0.753 |  | Low | 2.91 | 0.028 |
| s__Faecalibacterium_prausnitzii | 0.074 | 0.891 | -1.78 | 3.27 | -0.39 |  | -3.56 | 1.75 | 0.053 | 0.501 |  | Low | 4.35 | 0.034 |
| s__Clostridium_fessum | 0.099 | 0.902 | -1.30 | 2.73 | -0.27 |  | -2.19 | 1.83 | 0.243 | 0.756 |  | Low | 3.24 | 0.003 |
| s__Clostridium_sp_AF27_2AA | 0.126 | 0.872 | -4.54 | 12.70 | -0.46 |  | -6.81 | 2.62 | **0.016** | 0.501 |  | Low | 2.66 | 0.043 |
| s__Citrobacter_freundii | 0.154 | 0.858 | -3.34 | 8.69 | -0.39 |  | -5.57 | 1.93 | **0.008** | 0.501 |  | Low | 2.70 | 0.041 |
| s__Lacrimispora_saccharolytica | 0.177 | 0.894 | -5.09 | 10.20 | -0.51 |  | -6.20 | 2.17 | **0.009** | 0.501 |  | Low | 2.25 | 0.031 |
| s__Emergencia_timonensis | 0.194 | 0.886 | -3.55 | 9.24 | -0.44 |  | -5.90 | 1.95 | **0.006** | 0.501 |  | Low | 2.23 | 0.020 |
| s__Clostridium_sp_AM49_4BH | 0.202 | 0.920 | -5.42 | 15.06 | -0.56 |  | -6.63 | 3.00 | **0.037** | 0.501 |  | Low | 2.87 | 0.030 |
| s__Parabacteroides_johnsonii | 0.214 | 0.906 | -2.69 | 9.60 | -0.26 |  | -6.18 | 2.31 | **0.013** | 0.501 |  | Low | 2.72 | 0.041 |
| s__Parabacteroides_faecis | 0.217 | 0.897 | -2.64 | 8.78 | -0.28 |  | -5.27 | 1.92 | **0.011** | 0.501 |  | Low | 2.30 | 0.041 |
| s__Paraprevotella_clara | 0.292 | 0.922 | -2.54 | 8.85 | -0.25 |  | -6.00 | 2.28 | **0.015** | 0.501 |  | Low | 2.71 | 0.041 |
| s__Eubacterium_limosum | 0.296 | 0.911 | -2.32 | 7.98 | -0.34 |  | -4.29 | 1.55 | **0.011** | 0.501 |  | Low | 2.59 | 0.041 |
| s__Enterocloster_lavalensis | 0.312 | 0.922 | -2.37 | 8.43 | -0.27 |  | -4.54 | 1.83 | **0.021** | 0.501 |  | Low | 2.43 | 0.041 |
| s__GGB9614_SGB15049 | **0.043** | 0.872 | -2.76 | 11.43 | -0.32 |  | -4.83 | 2.33 | **0.049** | 0.501 |  |  |  |  |
| s__Christensenellaceae_bacterium | 0.056 | 0.864 | -6.40 | 11.39 | -0.60 |  | -5.90 | 2.23 | **0.014** | 0.501 |  |  |  |  |
| s__Anaerotruncus_colihominis | 0.066 | 0.883 | -9.90 | 11.93 | -0.73 |  | -6.88 | 2.46 | **0.010** | 0.501 |  |  |  |  |
| s__Parasutterella_excrementihominis | 0.079 | 0.886 | -6.12 | 12.97 | -0.51 |  | -5.97 | 2.66 | **0.034** | 0.501 |  |  |  |  |
| s__Phocea_massiliensis | 0.082 | 0.883 | -4.40 | 11.62 | -0.49 |  | -5.35 | 2.35 | **0.032** | 0.501 |  |  |  |  |
| s__Clostridium_phoceensis | 0.083 | 0.893 | -2.19 | 5.88 | -0.31 |  | -5.96 | 2.07 | **0.008** | 0.501 |  |  |  |  |
| s__Mesosutterella_multiformis | 0.100 | 0.893 | -7.77 | 13.83 | -0.61 |  | -6.46 | 2.80 | **0.030** | 0.501 |  |  |  |  |
| s__Roseburia_sp_AF02_12 | 0.108 | 0.899 | -5.72 | 14.17 | -0.46 |  | -6.15 | 2.88 | **0.043** | 0.501 |  |  |  |  |
| s__GGB9699_SGB15216 | 0.147 | 0.920 | -2.31 | 9.55 | -0.27 |  | -4.97 | 2.38 | **0.047** | 0.501 |  |  |  |  |
| s__Eubacterium_sp_NSJ_61 | 0.157 | 0.901 | -5.33 | 11.67 | -0.46 |  | -5.39 | 2.37 | **0.032** | 0.501 |  |  |  |  |
| s__Clostridiaceae_bacterium | 0.159 | 0.921 | -0.95 | 3.10 | -0.30 |  | -1.98 | 0.84 | **0.027** | 0.501 |  |  |  |  |
| s__Blautia_hydrogenotrophica | 0.177 | 0.912 | -4.37 | 13.38 | -0.40 |  | -5.48 | 2.60 | **0.046** | 0.501 |  |  |  |  |
| s__Candidatus_Pararuminococcus_gallinarum | 0.208 | 0.914 | -2.99 | 9.18 | -0.38 |  | -3.97 | 1.78 | **0.035** | 0.501 |  |  |  |  |
| s__Hydrogeniiclostidium_mannosilyticum | 0.215 | 0.924 | -5.72 | 12.92 | -0.53 |  | -5.45 | 2.47 | **0.037** | 0.501 |  |  |  |  |
| s__Hungatella_hathewayi | 0.227 | 0.929 | -9.42 | 14.90 | -0.61 |  | -6.19 | 2.92 | **0.045** | 0.501 |  |  |  |  |
| s__Holdemania_massiliensis | 0.228 | 0.917 | -2.85 | 10.57 | -0.35 |  | -4.76 | 2.06 | **0.030** | 0.501 |  |  |  |  |
| s__Bacteroides_timonensis | 0.236 | 0.910 | -3.04 | 9.83 | -0.35 |  | -4.70 | 2.03 | **0.029** | 0.501 |  |  |  |  |
| s__Bacteroides_uniformis | 0.256 | 0.939 | -0.98 | 3.02 | -0.31 |  | -1.75 | 0.81 | **0.041** | 0.501 |  |  |  |  |
| s__GGB51959_SGB72479 | 0.264 | 0.920 | -2.79 | 8.01 | -0.36 |  | -3.77 | 1.53 | **0.021** | 0.501 |  |  |  |  |
| s__Clostridiaceae_bacterium_NSJ_31 | 0.307 | 0.919 | -2.11 | 6.79 | -0.31 |  | -3.39 | 1.29 | **0.015** | 0.501 |  |  |  |  |
| s__Christensenella_massiliensis | 0.313 | 0.923 | -2.07 | 6.65 | -0.29 |  | -3.50 | 1.31 | **0.013** | 0.501 |  |  |  |  |
| s__Alistipes_senegalensis | 0.321 | 0.913 | -2.00 | 7.50 | -0.23 |  | -3.82 | 1.56 | **0.022** | 0.501 |  |  |  |  |
| s__Enterococcus_faecalis | 0.329 | 0.929 | -2.25 | 9.95 | -0.24 |  | -4.00 | 1.89 | **0.044** | 0.501 |  |  |  |  |
| s__Weissella_cibaria | 0.350 | 0.917 | -1.92 | 6.50 | -0.25 |  | -3.23 | 1.24 | **0.015** | 0.501 |  |  |  |  |
| s__Bacteroides_thetaiotaomicron | 0.353 | 0.953 | -1.02 | 3.65 | -0.21 |  | -2.00 | 0.97 | 0.050 | 0.501 |  |  |  |  |
| s__GGB3537_SGB4727 | 0.354 | 0.919 | -1.79 | 6.60 | -0.24 |  | -3.28 | 1.32 | **0.020** | 0.501 |  |  |  |  |
| s__Gemmiger_SGB15299 | 0.404 | 0.932 | -1.30 | 6.14 | -0.18 |  | -3.01 | 1.37 | **0.038** | 0.501 |  |  |  |  |
| s__Intestinibacillus_sp_Marseille_P6563 | 0.405 | 0.940 | -1.59 | 7.35 | -0.22 |  | -3.52 | 1.53 | **0.031** | 0.501 |  |  |  |  |
| s__Erysipelotrichaceae_bacterium_3_1_53 | 0.411 | 0.943 | -1.71 | 7.57 | -0.18 |  | -3.72 | 1.62 | **0.030** | 0.501 |  |  |  |  |
| s__GGB6649_SGB9391 | 0.431 | 0.944 | -1.17 | 5.78 | -0.19 |  | -2.40 | 0.98 | **0.022** | 0.501 |  |  |  |  |
| s__Lawsonibacter_sp_NSJ_51 | 0.441 | 0.933 | -1.13 | 5.80 | -0.16 |  | -2.69 | 1.17 | **0.030** | 0.501 |  |  |  |  |
| s__GGB45432_SGB63101 | 0.458 | 0.952 | -1.16 | 5.67 | -0.18 |  | -2.36 | 1.03 | **0.032** | 0.501 |  |  |  |  |
| s__GGB9695_SGB15209 | 0.458 | 0.954 | -1.02 | 5.90 | -0.14 |  | -2.54 | 1.16 | **0.038** | 0.501 |  |  |  |  |
| s__Christensenellaceae_bacterium_NSJ_44 | 0.462 | 0.951 | -1.01 | 5.97 | -0.13 |  | -2.42 | 1.15 | **0.046** | 0.501 |  |  |  |  |
| s__GGB33516_SGB54347 | 0.464 | 0.955 | -1.18 | 5.80 | -0.17 |  | -2.37 | 0.97 | **0.022** | 0.501 |  |  |  |  |
| s__GGB9694_SGB15204 | 0.464 | 0.949 | -1.16 | 5.87 | -0.15 |  | -2.92 | 1.39 | **0.046** | 0.501 |  |  |  |  |
| s__GGB9695_SGB15210 | 0.467 | 0.946 | -1.19 | 5.92 | -0.17 |  | -2.24 | 0.94 | **0.025** | 0.501 |  |  |  |  |
| s__Raoultibacter_massiliensis | 0.491 | 0.951 | -0.52 | 5.12 | -0.09 |  | -1.31 | 0.56 | **0.028** | 0.501 |  |  |  |  |
| s__GGB4710_SGB6522 | 0.495 | 0.957 | -0.69 | 5.20 | -0.11 |  | -1.45 | 0.69 | **0.046** | 0.501 |  |  |  |  |
| s__Pseudoflavonifractor_capillosus | 0.516 | 0.956 | -0.46 | 5.22 | -0.07 |  | -1.39 | 0.62 | **0.035** | 0.501 |  |  |  |  |
| s__Bifidobacterium_dentium | 0.519 | 0.955 | -0.40 | 5.12 | -0.08 |  | -1.36 | 0.60 | **0.033** | 0.501 |  |  |  |  |
| s__Kluyvera_georgiana | 0.526 | 0.961 | -0.61 | 5.15 | -0.10 |  | -1.20 | 0.49 | **0.021** | 0.501 |  |  |  |  |
| s__Citrobacter_amalonaticus | 0.534 | 0.955 | -0.80 | 5.35 | -0.13 |  | -1.42 | 0.65 | **0.038** | 0.501 |  |  |  |  |
| s__Bacteroides_faecalis | 0.536 | 0.952 | -0.64 | 5.13 | -0.12 |  | -1.41 | 0.65 | **0.041** | 0.501 |  |  |  |  |
| s__Clostridiales_bacterium_BX7 | 0.536 | 0.954 | -0.60 | 5.18 | -0.10 |  | -1.48 | 0.70 | **0.044** | 0.501 |  |  |  |  |
| s__GGB9640_SGB15115 | 0.538 | 0.961 | -0.34 | 5.32 | -0.05 |  | -1.42 | 0.64 | **0.037** | 0.501 |  |  |  |  |
| s__GGB45540_SGB63220 | 0.540 | 0.959 | -0.47 | 5.32 | -0.09 |  | -1.38 | 0.62 | **0.035** | 0.501 |  |  |  |  |
| s__Enterococcus_thailandicus | 0.559 | 0.964 | -0.63 | 5.22 | -0.12 |  | -1.32 | 0.57 | **0.029** | 0.501 |  |  |  |  |

**Table S9. Baseline metabolite markers of sVNT levels to the BNT162b2 vaccine at 6 months p.v.**

|  | 6-month sVNT level | | | | |
| --- | --- | --- | --- | --- | --- |
| Metabolite | >median | ≤median | Wilcox's *p* | log2FC | VIP |
| 2-Aminoisobutyric acid | 0.41 (0.23, 0.59) | 0.2 (0.12, 0.31) | <0.001 | 0.92 | 1.274 |
| 4-Acetamidobutanoic acid | 0.1 (0.07, 0.17) | 0.07 (0.04, 0.11) | 0.005 | 0.5 | 1.188 |
| 4-Aminohippuric acid | 0.02 (0.01, 0.03) | 0.01 (0.01, 0.02) | 0.002 | 0.93 | 1.28 |
| Aminolevulinic acid | 0.41 (0.32, 0.55) | 0.29 (0.19, 0.4) | 0.024 | 0.34 | 1.027 |
| Benzenebutanoic acid | 0 (0, 0) | 0 (0, 0.01) | 0.02 | -0.53 | 1.002 |
| Bicine | 0.72 (0.52, 1.01) | 0.47 (0.38, 0.72) | 0.012 | 0.52 | 1.014 |
| Butyric acid | 48409.01 (30804.27, 74483) | 67595.93 (45235.62, 136281.24) | 0.029 | -0.71 | 1.644 |
| Carnitine(C0) | 0.13 (0.08, 0.31) | 0.08 (0.05, 0.14) | 0.009 | 0.7 | 1.111 |
| Carnosine | 0.12 (0.08, 0.24) | 0.05 (0.02, 0.14) | 0.004 | 1.49 | 1.07 |
| D-Maltose | 2.59 (0.45, 5.61) | 0.53 (0.34, 1.18) | 0.012 | 1.72 | 1.146 |
| Dehydrolithocholic acid | 14.47 (2.55, 44.11) | 2.54 (1.49, 7.21) | 0.008 | 1.61 | 1.15 |
| Dimethylglycine | 0.12 (0.1, 0.17) | 0.09 (0.06, 0.13) | 0.013 | 0.41 | 1.21 |
| Erythronic acid | 1.15 (0.51, 3.28) | 0.52 (0.24, 1.01) | 0.006 | 1.2 | 1.035 |
| Fructose 6-phosphate | 0.1 (0.07, 0.18) | 0.07 (0.05, 0.11) | 0.017 | 0.43 | 1.018 |
| Fumaric acid | 0.08 (0.06, 0.11) | 0.05 (0.04, 0.08) | 0.002 | 0.59 | 1.347 |
| Gamma-Aminobutyric acid | 1.91 (0.78, 3.05) | 0.81 (0.53, 1.6) | 0.008 | 1.08 | 1.172 |
| Glyceraldehyde | 1.76 (1.3, 2.18) | 1.19 (1.06, 1.54) | 0.002 | 0.53 | 1.348 |
| Glycolic acid | 1.14 (0.9, 1.61) | 0.91 (0.53, 1.28) | 0.014 | 0.46 | 1.071 |
| Isobutyrate | 18620.54 (12293.73, 29353.43) | 26897.54 (18147.33, 54894.96) | 0.026 | -0.71 | 1.65 |
| Isovalerate | 9622.98 (4374.06, 17566.68) | 17214.43 (9090.54, 37007.11) | 0.041 | -1.03 | 1.199 |
| m-Coumaric acid | 0.14 (0.09, 0.23) | 0.09 (0.05, 0.14) | 0.018 | 0.6 | 1.062 |
| Maleic acid | 0.26 (0.18, 0.36) | 0.17 (0.1, 0.29) | 0.017 | 0.65 | 1.145 |
| Melibiose | 0.05 (0.04, 0.07) | 0.03 (0.02, 0.05) | 0.011 | 0.51 | 1.226 |
| N-Acetyl-L-aspartic acid | 7.81 (5.75, 10.91) | 4.02 (2.04, 7.32) | 0.002 | 0.75 | 1.069 |
| N-Acetylproline | 0.5 (0.3, 0.78) | 0.21 (0.1, 0.46) | 0.003 | 0.65 | 1.207 |
| Nicotinic acid | 0.77 (0.56, 1.39) | 0.39 (0.18, 0.6) | 0.001 | 0.7 | 1.269 |
| S-Carboxymethyl-L-cysteine | 1.36 (1.09, 1.62) | 0.94 (0.68, 1.37) | 0.032 | 0.37 | 1.025 |
| Threonic acid | 0.31 (0.2, 0.55) | 0.14 (0.08, 0.31) | 0.002 | 0.83 | 1.161 |

**Table S10. Correlations between age and baseline concentration of potential metabolite markers in the BNT162b2 group.**

| Feature | Spearman's Rho | P value |
| --- | --- | --- |
| 2-Aminoisobutyric acid | -0.12 | 0.295 |
| 4-Acetamidobutanoic acid | -0.08 | 0.465 |
| 4-Aminohippuric acid | -0.17 | 0.131 |
| Aminolevulinic acid | -0.10 | 0.393 |
| Benzenebutanoic acid | 0.17 | 0.136 |
| Bicine | -0.08 | 0.466 |
| Butyric acid | -0.08 | 0.481 |
| Carnitine(C0) | -0.09 | 0.436 |
| Carnosine | -0.16 | 0.168 |
| D-Maltose | -0.13 | 0.273 |
| Dehydrolithocholic acid | -0.10 | 0.394 |
| Dimethylglycine | -0.16 | 0.159 |
| Erythronic acid | -0.13 | 0.265 |
| Fructose 6-phosphate | -0.08 | 0.468 |
| Fumaric acid | -0.02 | 0.838 |
| Gamma-Aminobutyric acid | -0.14 | 0.223 |
| Glyceraldehyde | 0.08 | 0.490 |
| Glycolic acid | 0.02 | 0.864 |
| Isobutyrate | -0.07 | 0.539 |
| Isovalerate | -0.10 | 0.397 |
| m-Coumaric acid | -0.03 | 0.808 |
| Maleic acid | 0.07 | 0.555 |
| Melibiose | -0.12 | 0.279 |
| N-Acetyl-L-aspartic acid | 0.01 | 0.897 |
| N-Acetylproline | -0.09 | 0.454 |
| Nicotinic acid | -0.05 | 0.692 |
| S-Carboxymethyl-L-cysteine | -0.16 | 0.169 |
| Threonic acid | -0.09 | 0.429 |

**Table S11. Baseline metabolite markers of sVNT levels to the CoronaVac vaccine at 6 months p.v.**

|  | 6-month sVNT level | | | | |
| --- | --- | --- | --- | --- | --- |
| Metabolite | >median | ≤median | Wilcox's *p* | log2FC | VIP |
| 12-Dehydrocholic Acid Diacetate | 0 (0, 0) | 0.01 (0.01, 0.01) | 0.005 | -1.36 | 1.481 |
| 13C,16C-DOCOSADIENOIC ACID | 0 (0, 0) | 0 (0, 0) | 0.037 | -0.91 | 1.027 |
| 2-Aminoisobutyric acid | 0.14 (0.09, 0.39) | 0.55 (0.33, 0.82) | 0.02 | -1.42 | 1.599 |
| 23-Norcholic Acid Diacetate | 0.01 (0.01, 0.01) | 0.02 (0.01, 0.02) | 0.001 | -1.32 | 1.411 |
| 23-Nordeoxycholic Acid Diacetate | 0 (0, 0) | 0 (0, 0) | 0.002 | -1.47 | 1.5 |
| 3-Methyl-2-oxovaleric acid | 0.05 (0.03, 0.06) | 0.16 (0.08, 0.24) | 0.015 | -2.12 | 1.178 |
| 3-Nitrotyrosine | 0.06 (0.04, 0.07) | 0.09 (0.07, 0.09) | 0.027 | -0.66 | 1.089 |
| 3-Pyridylacetic acid | 0 (0, 0) | 0 (0, 0.01) | 0.03 | -1.71 | 1.447 |
| 3,4,5-Trimethoxycinnamic acid | 0 (0, 0) | 0 (0, 0.01) | 0.02 | -0.95 | 1.035 |
| Azelaic acid | 0.23 (0.2, 0.24) | 0.81 (0.67, 0.9) | 0.003 | -1.74 | 1.48 |
| Carnitine(C0) | 0.05 (0.02, 0.06) | 0.1 (0.09, 0.12) | 0.001 | -1.33 | 1.432 |
| Citrulline | 1.61 (1.31, 2.28) | 4.77 (3.18, 7.17) | 0.027 | -1.51 | 1.312 |
| Dehydrolithocholic Acid Methyl Ester | 0.48 (0.42, 0.57) | 0.38 (0.32, 0.4) | 0.048 | 0.46 | 1.461 |
| Dimethylglycine | 0.08 (0.02, 0.1) | 0.11 (0.1, 0.18) | 0.05 | -1.24 | 1.272 |
| Docosatrienoic acid | 0.01 (0.01, 0.01) | 0.06 (0.03, 0.1) | 0.015 | -1.57 | 1.123 |
| Etiadienic Acid 3-Acetate | 0 (0, 0) | 0 (0, 0) | 0.048 | -0.76 | 1.084 |
| Etienic Acid Acetate | 0 (0, 0) | 0 (0, 0) | 0 | -1.26 | 1.606 |
| Glutaric acid | 0.69 (0.36, 1.25) | 1.97 (1.17, 2.49) | 0.048 | -1.34 | 1.071 |
| Glyco-Lambda-Muricholic Acid | 0.51 (0.32, 0.52) | 0.82 (0.64, 0.97) | 0.015 | -0.78 | 1.138 |
| Hexanylcarnitine | 0 (0, 0) | 0 (0, 0) | 0.015 | -0.64 | 1.074 |
| Hyocholic Acid Methyl Ester | 0.09 (0.05, 0.14) | 0.04 (0.04, 0.05) | 0.048 | 1 | 1.585 |
| Hyodeoxycholic Acid Methyl Ester | 0.09 (0.06, 0.13) | 0.05 (0.04, 0.06) | 0.048 | 0.95 | 1.577 |
| Itaconic acid | 0 (0, 0.01) | 0.01 (0.01, 0.01) | 0.01 | -1.14 | 1.081 |
| Ketoleucine | 0.04 (0.03, 0.05) | 0.14 (0.07, 0.29) | 0.02 | -2.28 | 1.097 |
| L-Acetylcarnitine | 0.01 (0, 0.01) | 0.02 (0.01, 0.03) | 0.027 | -1.43 | 1.038 |
| L-Glutamine | 0.54 (0.5, 0.66) | 2.13 (1.32, 2.76) | 0.005 | -1.8 | 1.814 |
| L-Homoserine | 0.21 (0.04, 0.5) | 0.74 (0.59, 1.28) | 0.027 | -1.56 | 1.14 |
| L-Pipecolic acid | 0.81 (0.72, 1.08) | 2.49 (1.14, 2.94) | 0.037 | -1.4 | 1.152 |
| L-Proline | 2.3 (1.71, 2.61) | 3.75 (2.91, 4.5) | 0.02 | -0.91 | 1.165 |
| L-Serine | 3.13 (2.04, 3.9) | 6.53 (4.22, 8.83) | 0.02 | -1.11 | 1.087 |
| L-Tryptophan | 9.76 (7.73, 11.78) | 19.98 (19.26, 22.08) | 0.048 | -0.81 | 1.176 |
| N-Methyl-D-aspartic acid | 0.69 (0.48, 0.76) | 1.69 (1.22, 2.24) | 0.002 | -1.58 | 1.415 |
| N-Phenylacetylphenylalanine | 0 (0, 0) | 0.01 (0, 0.01) | 0.02 | -1.09 | 1.193 |
| Nordeoxycholic Acid | 0 (0, 0) | 0 (0, 0) | 0.037 | -1.34 | 1.303 |
| Oxoadipic acid | 0.01 (0.01, 0.01) | 0.04 (0.03, 0.05) | 0.003 | -1.75 | 1.432 |
| Phosphoserine | 0 (0, 0) | 0.01 (0.01, 0.01) | 0.007 | -2.25 | 1.332 |
| Sarcosine | 0.51 (0.33, 0.65) | 1 (0.58, 1.42) | 0.027 | -1.09 | 1.167 |
| Sebacic acid | 0.04 (0.04, 0.07) | 0.18 (0.11, 0.23) | 0.027 | -1.13 | 1.326 |
| Succinic acid | 17.67 (8.53, 21.58) | 6.46 (3.87, 7.53) | 0.048 | 1.3 | 1.781 |
| Tauro-omega-Muricholic Acid | 0.89 (0.69, 0.94) | 1.32 (1, 1.67) | 0.048 | -0.88 | 1.077 |
| Tridecanoic acid | 0.04 (0.03, 0.04) | 0.11 (0.08, 0.19) | 0.048 | -1.67 | 1.076 |
| Vanillymandelic Acid | 0.11 (0.08, 0.13) | 0.35 (0.23, 0.51) | 0.01 | -1.84 | 1.218 |

**Table S12. Correlations between age and baseline concentration of potential metabolite markers in the CoronaVac group.**

| Feature | Spearman's Rho | P value |
| --- | --- | --- |
| 12-Dehydrocholic Acid Diacetate | 0.08 | 0.764 |
| 13C,16C-DOCOSADIENOIC ACID | 0.00 | 0.985 |
| 2-Aminoisobutyric acid | -0.04 | 0.866 |
| 23-Norcholic Acid Diacetate | -0.02 | 0.937 |
| 23-Nordeoxycholic Acid Diacetate | 0.04 | 0.892 |
| 3-Methyl-2-oxovaleric acid | -0.10 | 0.708 |
| 3-Nitrotyrosine | 0.08 | 0.764 |
| 3-Pyridylacetic acid | 0.32 | 0.216 |
| 3,4,5-Trimethoxycinnamic acid | 0.06 | 0.808 |
| Azelaic acid | 0.14 | 0.583 |
| Carnitine(C0) | 0.20 | 0.447 |
| Citrulline | -0.07 | 0.786 |
| Dehydrolithocholic Acid Methyl Ester | 0.20 | 0.442 |
| Dimethylglycine | 0.10 | 0.701 |
| Docosatrienoic acid | 0.19 | 0.474 |
| Etiadienic Acid 3-Acetate | 0.07 | 0.797 |
| Etienic Acid Acetate | 0.15 | 0.573 |
| Glutaric acid | 0.32 | 0.210 |
| Glyco-Lambda-Muricholic Acid | 0.17 | 0.510 |
| Hexanylcarnitine | 0.08 | 0.772 |
| Hyocholic Acid Methyl Ester | 0.13 | 0.615 |
| Hyodeoxycholic Acid Methyl Ester | 0.11 | 0.683 |
| Itaconic acid | 0.49 | **0.047** |
| Ketoleucine | -0.07 | 0.775 |
| L-Acetylcarnitine | -0.07 | 0.804 |
| L-Glutamine | 0.35 | 0.166 |
| L-Homoserine | 0.33 | 0.203 |
| L-Pipecolic acid | 0.11 | 0.687 |
| L-Proline | -0.03 | 0.896 |
| L-Serine | 0.19 | 0.462 |
| L-Tryptophan | 0.19 | 0.468 |
| N-Methyl-D-aspartic acid | 0.28 | 0.268 |
| N-Phenylacetylphenylalanine | 0.13 | 0.612 |
| Nordeoxycholic Acid | 0.01 | 0.966 |
| Oxoadipic acid | 0.30 | 0.235 |
| Phosphoserine | -0.25 | 0.333 |
| Sarcosine | 0.18 | 0.479 |
| Sebacic acid | -0.01 | 0.970 |
| Succinic acid | 0.00 | 0.989 |
| Tauro-omega-Muricholic Acid | 0.08 | 0.772 |
| Tridecanoic acid | -0.03 | 0.922 |
| Vanillymandelic Acid | 0.27 | 0.290 |

**Table S13. Correlations between the abundance of *Bifidobacterium adolescentis* and concentrations of metabolite markers at baseline.**

| Vaccine group | Metabolite | Spearman's Rho | P value | FDR |
| --- | --- | --- | --- | --- |
| BNT162b2 | Erythronic acid | 0.35 | 0.002 | **0.037** |
|  | m-Coumaric acid | 0.32 | 0.004 | **0.037** |
|  | Threonic acid | 0.32 | 0.004 | **0.037** |
|  | Glyceraldehyde | 0.28 | 0.013 | **0.063** |
|  | Bicine | 0.28 | 0.014 | **0.063** |
|  | S-Carboxymethyl-L-cysteine | 0.28 | 0.014 | **0.063** |
|  | Glycolic acid | 0.25 | 0.026 | **0.092** |
|  | N-Acetyl-L-aspartic acid | 0.25 | 0.027 | **0.092** |
|  | D-Maltose | 0.25 | 0.03 | **0.092** |
|  | Gamma-Aminobutyric acid | 0.24 | 0.038 | 0.106 |
|  | Fructose 6-phosphate | 0.23 | 0.045 | 0.108 |
|  | Carnosine | 0.23 | 0.046 | 0.108 |
|  | 4-Aminohippuric acid | 0.22 | 0.054 | 0.115 |
|  | Aminolevulinic acid | 0.19 | 0.101 | 0.202 |
|  | Nicotinic acid | 0.17 | 0.126 | 0.235 |
|  | 2-Aminoisobutyric acid | 0.16 | 0.152 | 0.266 |
|  | Dehydrolithocholic acid | 0.16 | 0.165 | 0.272 |
|  | Benzenebutanoic acid | -0.15 | 0.205 | 0.314 |
|  | Melibiose | 0.14 | 0.213 | 0.314 |
|  | 4-Acetamidobutanoic acid | 0.13 | 0.26 | 0.364 |
|  | Isovalerate | -0.11 | 0.329 | 0.438 |
|  | Dimethylglycine | 0.10 | 0.361 | 0.447 |
|  | N-Acetylproline | 0.10 | 0.367 | 0.447 |
|  | Maleic acid | -0.10 | 0.396 | 0.462 |
|  | Carnitine(C0) | 0.09 | 0.456 | 0.511 |
|  | Isobutyrate | 0.02 | 0.867 | 0.933 |
|  | Butyric acid | 0.01 | 0.928 | 0.936 |
|  | Fumaric acid | -0.01 | 0.936 | 0.936 |
| CoronaVac | L-Acetylcarnitine | 0.61 | 0.009 | 0.179 |
|  | 12-Dehydrocholic Acid Diacetate | 0.39 | 0.123 | 0.37 |
|  | L-Homoserine | 0.29 | 0.264 | 0.49 |
|  | 3,4,5-Trimethoxycinnamic acid | 0.16 | 0.533 | 0.687 |
|  | 3-Nitrotyrosine | 0.33 | 0.199 | 0.449 |
|  | 23-Nordeoxycholic Acid Diacetate | 0.49 | 0.047 | 0.259 |
|  | Glyco-Lambda-Muricholic Acid | 0.17 | 0.507 | 0.687 |
|  | Glutaric acid | 0.33 | 0.203 | 0.449 |
|  | N-Phenylacetylphenylalanine | 0.27 | 0.291 | 0.49 |
|  | 23-Norcholic Acid Diacetate | 0.44 | 0.078 | 0.276 |
|  | L-Serine | 0.34 | 0.185 | 0.449 |
|  | Tauro-omega-Muricholic Acid | 0.37 | 0.14 | 0.392 |
|  | Carnitine(C0) | 0.46 | 0.062 | 0.259 |
|  | 13C,16C-DOCOSADIENOIC ACID | 0.57 | 0.018 | 0.187 |
|  | Ketoleucine | 0.53 | 0.027 | 0.231 |
|  | 3-Pyridylacetic acid | 0.20 | 0.452 | 0.687 |
|  | 3-Methyl-2-oxovaleric acid | 0.60 | 0.011 | 0.179 |
|  | L-Pipecolic acid | 0.43 | 0.082 | 0.276 |
|  | Etiadienic Acid 3-Acetate | 0.52 | 0.034 | 0.239 |
|  | L-Tryptophan | 0.27 | 0.287 | 0.49 |
|  | Citrulline | 0.59 | 0.013 | 0.179 |
|  | Hexanylcarnitine | 0.29 | 0.26 | 0.49 |
|  | Nordeoxycholic Acid | 0.43 | 0.086 | 0.276 |
|  | Etienic Acid Acetate | 0.22 | 0.389 | 0.628 |
|  | Docosatrienoic acid | 0.28 | 0.278 | 0.49 |
|  | Sebacic acid | 0.46 | 0.06 | 0.259 |
|  | Sarcosine | 0.29 | 0.251 | 0.49 |
|  | Tridecanoic acid | 0.47 | 0.057 | 0.259 |
|  | L-Proline | 0.35 | 0.171 | 0.449 |
|  | Dehydrolithocholic Acid Methyl Ester | -0.16 | 0.533 | 0.687 |
|  | Hyodeoxycholic Acid Methyl Ester | -0.16 | 0.533 | 0.687 |
|  | Oxoadipic acid | 0.16 | 0.533 | 0.687 |
|  | Itaconic acid | 0.16 | 0.54 | 0.687 |
|  | Succinic acid | -0.15 | 0.566 | 0.699 |
|  | Azelaic acid | 0.14 | 0.593 | 0.7 |
|  | Hyocholic Acid Methyl Ester | -0.14 | 0.6 | 0.7 |
|  | N-Methyl-D-aspartic acid | 0.10 | 0.698 | 0.792 |
|  | 2-Aminoisobutyric acid | 0.08 | 0.749 | 0.807 |
|  | L-Glutamine | -0.08 | 0.749 | 0.807 |
|  | Vanillymandelic Acid | -0.01 | 0.969 | 0.992 |
|  | Dimethylglycine | 0.00 | 0.992 | 0.992 |
|  | Phosphoserine | 0.00 | 0.992 | 0.992 |

**Table S14. Correlations between the abundances of 1-month relative abundances of bacteria and sVNT levels at 6 months p.v. in the CoronaVac group.**

|  |  | 6-month sVNT | | | |  | Relative drop (from 1 month to 6 months) | | | |
| --- | --- | --- | --- | --- | --- | --- | --- | --- | --- | --- |
| Species | N (%) | Spearman's Rho | | P value | FDR |  | Spearman's Rho | | P value | FDR |
| s__Dorea_formicigenerans | 36 (92.3) | 0.62 | 0.001 | | 0.123 |  | -0.60 | 0.001 | | 0.123 |
| s__Eisenbergiella_massiliensis | 10 (25.6) | -0.59 | 0.002 | | 0.123 |  | 0.58 | 0.002 | | 0.128 |
| s__Anaerotruncus_colihominis | 16 (41) | -0.58 | 0.002 | | 0.123 |  | 0.60 | 0.001 | | 0.123 |
| s__Bilophila_wadsworthia | 38 (97.4) | -0.52 | 0.007 | | 0.360 |  | 0.51 | 0.008 | | 0.400 |
| s__Bacteroides_caccae | 26 (66.7) | -0.44 | 0.023 | | 0.520 |  | 0.41 | 0.038 | | 0.601 |
| s__Bacteroides_salyersiae | 14 (35.9) | 0.44 | 0.023 | | 0.520 |  | -0.35 | 0.076 | | 0.628 |
| s__Bacteroides_thetaiotaomicron | 39 (100) | -0.40 | 0.046 | | 0.520 |  | 0.38 | 0.056 | | 0.628 |
| s__Phocaeicola_dorei | 17 (43.6) | 0.42 | 0.034 | | 0.520 |  | -0.34 | 0.087 | | 0.628 |
| s__Christensenellaceae_bacterium | 17 (43.6) | -0.39 | 0.052 | | 0.520 |  | 0.41 | 0.036 | | 0.601 |
| s__Clostridium_phoceensis | 34 (87.2) | -0.42 | 0.035 | | 0.520 |  | 0.49 | 0.012 | | 0.403 |
| s__Hungatella_hathewayi | 18 (46.2) | -0.42 | 0.031 | | 0.520 |  | 0.41 | 0.038 | | 0.601 |
| s__Clostridiales_unclassified_SGB15145 | 21 (53.8) | 0.39 | 0.048 | | 0.520 |  | -0.40 | 0.041 | | 0.601 |
| s__Intestinimonas_butyriciproducens | 21 (53.8) | -0.47 | 0.015 | | 0.520 |  | 0.50 | 0.010 | | 0.400 |
| s__Coprococcus_catus | 21 (53.8) | 0.42 | 0.033 | | 0.520 |  | -0.33 | 0.101 | | 0.640 |
| s__Enterocloster_asparagiformis | 10 (25.6) | -0.44 | 0.023 | | 0.520 |  | 0.41 | 0.038 | | 0.601 |
| s__Enterocloster_citroniae | 24 (61.5) | -0.39 | 0.050 | | 0.520 |  | 0.41 | 0.039 | | 0.601 |
| s__Fusicatenibacter_saccharivorans | 36 (92.3) | 0.39 | 0.050 | | 0.520 |  | -0.34 | 0.085 | | 0.628 |
| s__GGB3746_SGB5089 | 20 (51.3) | 0.40 | 0.045 | | 0.520 |  | -0.38 | 0.057 | | 0.628 |
| s__Roseburia_hominis | 29 (74.4) | -0.38 | 0.054 | | 0.520 |  | 0.34 | 0.088 | | 0.628 |
| s__Phocea_massiliensis | 26 (66.7) | -0.44 | 0.024 | | 0.520 |  | 0.41 | 0.037 | | 0.601 |
| s__Parasutterella_excrementihominis | 23 (59) | -0.41 | 0.039 | | 0.520 |  | 0.39 | 0.051 | | 0.628 |
| s__Clostridiales_bacterium_KLE1615 | 35 (89.7) | -0.37 | 0.064 | | 0.550 |  | 0.35 | 0.080 | | 0.628 |
| s__Enterocloster_aldensis | 14 (35.9) | -0.36 | 0.068 | | 0.550 |  | 0.35 | 0.080 | | 0.628 |
| s__Ruminococcaceae_bacterium_AM07_15 | 14 (35.9) | 0.37 | 0.065 | | 0.550 |  | -0.34 | 0.090 | | 0.628 |
| s__Mesosutterella_multiformis | 15 (38.5) | -0.37 | 0.061 | | 0.550 |  | 0.33 | 0.100 | | 0.640 |
| s__Anaeromassilibacillus_sp_An250 | 10 (25.6) | -0.35 | 0.080 | | 0.628 |  | 0.41 | 0.039 | | 0.601 |
| s__Clostridium_sp_AM22_11AC | 30 (76.9) | 0.34 | 0.091 | | 0.668 |  | -0.35 | 0.078 | | 0.628 |
| s__Eubacterium_sp_NSJ_61 | 10 (25.6) | -0.33 | 0.097 | | 0.668 |  | 0.29 | 0.156 | | 0.699 |
| s__Flavonifractor_plautii | 36 (92.3) | -0.33 | 0.102 | | 0.668 |  | 0.38 | 0.057 | | 0.628 |
| s__Veillonella_parvula | 11 (28.2) | 0.33 | 0.100 | | 0.668 |  | -0.28 | 0.174 | | 0.699 |
| s__Sutterella_wadsworthensis | 20 (51.3) | 0.33 | 0.098 | | 0.668 |  | -0.34 | 0.090 | | 0.628 |
| s__Bacteroides_fragilis | 18 (46.2) | -0.32 | 0.110 | | 0.700 |  | 0.36 | 0.073 | | 0.628 |
| s__GGB9557_SGB14966 | 18 (46.2) | -0.31 | 0.121 | | 0.723 |  | 0.31 | 0.126 | | 0.699 |
| s__Anaerobutyricum_hallii | 38 (97.4) | -0.31 | 0.118 | | 0.723 |  | 0.33 | 0.096 | | 0.640 |
| s__Eggerthellaceae_unclassified_SGB14341 | 11 (28.2) | -0.29 | 0.157 | | 0.742 |  | 0.30 | 0.137 | | 0.699 |
| s__Gordonibacter_pamelaeae | 14 (35.9) | -0.31 | 0.129 | | 0.742 |  | 0.29 | 0.148 | | 0.699 |
| s__Bacteroides_intestinalis | 21 (53.8) | -0.30 | 0.142 | | 0.742 |  | 0.26 | 0.201 | | 0.720 |
| s__Streptococcus_sp_A12 | 17 (43.6) | 0.28 | 0.164 | | 0.742 |  | -0.26 | 0.199 | | 0.720 |
| s__Candidatus_Pararuminococcus_gallinarum | 11 (28.2) | -0.30 | 0.134 | | 0.742 |  | 0.26 | 0.203 | | 0.720 |
| s__Clostridia_unclassified_SGB4447 | 16 (41) | 0.29 | 0.151 | | 0.742 |  | -0.28 | 0.162 | | 0.699 |
| s__Blautia_glucerasea | 17 (43.6) | -0.28 | 0.164 | | 0.742 |  | 0.24 | 0.241 | | 0.740 |
| s__Eisenbergiella_tayi | 12 (30.8) | -0.30 | 0.139 | | 0.742 |  | 0.30 | 0.131 | | 0.699 |
| s__Intestinibacter_bartlettii | 12 (30.8) | -0.29 | 0.144 | | 0.742 |  | 0.35 | 0.080 | | 0.628 |
| s__Faecalibacterium_SGB15346 | 29 (74.4) | 0.28 | 0.159 | | 0.742 |  | -0.24 | 0.228 | | 0.740 |
| s__Ruminococcaceae_bacterium | 24 (61.5) | -0.29 | 0.156 | | 0.742 |  | 0.28 | 0.165 | | 0.699 |
| s__Ruminococcaceae_unclassified_SGB15265 | 19 (48.7) | -0.27 | 0.175 | | 0.770 |  | 0.30 | 0.141 | | 0.699 |
| s__Bacteroides_nordii | 25 (64.1) | -0.27 | 0.183 | | 0.775 |  | 0.27 | 0.179 | | 0.699 |
| s__Blautia_wexlerae | 39 (100) | -0.27 | 0.183 | | 0.775 |  | 0.27 | 0.179 | | 0.699 |
| s__Enterocloster_bolteae | 23 (59) | -0.26 | 0.195 | | 0.791 |  | 0.29 | 0.154 | | 0.699 |
| s__Megamonas_funiformis | 18 (46.2) | 0.26 | 0.192 | | 0.791 |  | -0.25 | 0.213 | | 0.720 |
| s__Ruminococcus_torques | 35 (89.7) | 0.26 | 0.202 | | 0.802 |  | -0.24 | 0.229 | | 0.740 |
| s__Bacteroides_cellulosilyticus | 23 (59) | -0.25 | 0.215 | | 0.807 |  | 0.30 | 0.141 | | 0.699 |
| s__Alistipes_indistinctus | 26 (66.7) | -0.26 | 0.208 | | 0.807 |  | 0.28 | 0.159 | | 0.699 |
| s__Clostridium_fessum | 37 (94.9) | 0.25 | 0.215 | | 0.807 |  | -0.20 | 0.331 | | 0.795 |
| s__Clostridium_sp_AM49_4BH | 12 (30.8) | -0.25 | 0.219 | | 0.807 |  | 0.22 | 0.283 | | 0.767 |
| s__Lacrimispora_amygdalina | 35 (89.7) | 0.25 | 0.225 | | 0.815 |  | -0.19 | 0.353 | | 0.796 |
| s__Bacteroides_finegoldii | 19 (48.7) | 0.24 | 0.230 | | 0.820 |  | -0.20 | 0.315 | | 0.790 |
| s__Eggerthella_lenta | 23 (59) | -0.24 | 0.240 | | 0.827 |  | 0.23 | 0.252 | | 0.746 |
| s__Ruminococcaceae_unclassified_SGB15260 | 17 (43.6) | -0.24 | 0.240 | | 0.827 |  | 0.19 | 0.359 | | 0.796 |
| s__Bifidobacterium_adolescentis | 21 (53.8) | 0.21 | 0.311 | | 0.847 |  | -0.18 | 0.388 | | 0.829 |
| s__Bifidobacterium_pseudocatenulatum | 30 (76.9) | 0.19 | 0.347 | | 0.847 |  | -0.13 | 0.531 | | 0.869 |
| s__Bacteroides_uniformis | 39 (100) | -0.23 | 0.267 | | 0.847 |  | 0.23 | 0.267 | | 0.753 |
| s__Alistipes_finegoldii | 20 (51.3) | -0.19 | 0.351 | | 0.847 |  | 0.24 | 0.238 | | 0.740 |
| s__Candidatus_Avimicrobium_caecorum | 28 (71.8) | -0.21 | 0.306 | | 0.847 |  | 0.23 | 0.254 | | 0.746 |
| s__Clostridia_bacterium | 38 (97.4) | -0.23 | 0.264 | | 0.847 |  | 0.28 | 0.164 | | 0.699 |
| s__Butyricicoccus_sp_AM29_23AC | 12 (30.8) | 0.19 | 0.349 | | 0.847 |  | -0.22 | 0.274 | | 0.758 |
| s__Clostridium_sp_AF20_17LB | 25 (64.1) | 0.21 | 0.300 | | 0.847 |  | -0.24 | 0.231 | | 0.740 |
| s__Clostridium_sp_AF27_2AA | 14 (35.9) | -0.20 | 0.325 | | 0.847 |  | 0.25 | 0.212 | | 0.720 |
| s__Clostridium_sp_AM33_3 | 29 (74.4) | 0.19 | 0.345 | | 0.847 |  | -0.26 | 0.203 | | 0.720 |
| s__Clostridium_sp_AM42_4 | 23 (59) | 0.21 | 0.303 | | 0.847 |  | -0.15 | 0.456 | | 0.850 |
| s__Clostridium_sp_AT4 | 12 (30.8) | 0.21 | 0.304 | | 0.847 |  | -0.14 | 0.505 | | 0.862 |
| s__Clostridiales_bacterium | 29 (74.4) | -0.23 | 0.259 | | 0.847 |  | 0.30 | 0.140 | | 0.699 |
| s__Evtepia_gabavorous | 25 (64.1) | 0.22 | 0.287 | | 0.847 |  | -0.20 | 0.327 | | 0.795 |
| s__Eubacterium_ramulus | 32 (82.1) | 0.22 | 0.283 | | 0.847 |  | -0.29 | 0.156 | | 0.699 |
| s__Coprococcus_comes | 28 (71.8) | -0.21 | 0.313 | | 0.847 |  | 0.12 | 0.556 | | 0.874 |
| s__Dorea_sp_AF24_7LB | 10 (25.6) | 0.22 | 0.273 | | 0.847 |  | -0.31 | 0.124 | | 0.699 |
| s__Dorea_sp_AF36_15AT | 16 (41) | 0.22 | 0.277 | | 0.847 |  | -0.26 | 0.198 | | 0.720 |
| s__Lachnospira_sp_NSJ_43 | 20 (51.3) | -0.20 | 0.331 | | 0.847 |  | 0.20 | 0.337 | | 0.795 |
| s__Mediterraneibacter_sp_gm002 | 12 (30.8) | 0.22 | 0.287 | | 0.847 |  | -0.27 | 0.176 | | 0.699 |
| s__Ruminococcus_lactaris | 15 (38.5) | -0.22 | 0.290 | | 0.847 |  | 0.24 | 0.242 | | 0.740 |
| s__Faecalibacterium_prausnitzii | 39 (100) | 0.19 | 0.339 | | 0.847 |  | -0.16 | 0.444 | | 0.843 |
| s__Ruthenibacterium_lactatiformans | 33 (84.6) | -0.23 | 0.265 | | 0.847 |  | 0.28 | 0.169 | | 0.699 |
| s__Megasphaera_sp_BL7 | 12 (30.8) | -0.20 | 0.335 | | 0.847 |  | 0.19 | 0.345 | | 0.796 |
| s__Haemophilus_parainfluenzae | 20 (51.3) | 0.20 | 0.336 | | 0.847 |  | -0.14 | 0.503 | | 0.862 |
| s__Parabacteroides_distasonis | 38 (97.4) | -0.19 | 0.362 | | 0.849 |  | 0.22 | 0.290 | | 0.775 |
| s__Lachnospiraceae_bacterium_WCA3_601_WT_6H | 29 (74.4) | -0.19 | 0.364 | | 0.849 |  | 0.13 | 0.522 | | 0.862 |
| s__GGB9501_SGB14898 | 10 (25.6) | -0.19 | 0.363 | | 0.849 |  | 0.16 | 0.433 | | 0.841 |
| s__Alistipes_onderdonkii | 22 (56.4) | 0.18 | 0.382 | | 0.861 |  | -0.26 | 0.208 | | 0.720 |
| s__Mediterraneibacter_butyricigenes | 10 (25.6) | 0.18 | 0.378 | | 0.861 |  | -0.21 | 0.315 | | 0.790 |
| s__Holdemania_massiliensis | 13 (33.3) | -0.18 | 0.375 | | 0.861 |  | 0.21 | 0.313 | | 0.790 |
| s__Collinsella_aerofaciens | 30 (76.9) | 0.14 | 0.507 | | 0.880 |  | -0.13 | 0.520 | | 0.862 |
| s__Collinsella_tanakaei | 12 (30.8) | 0.13 | 0.520 | | 0.880 |  | -0.18 | 0.387 | | 0.829 |
| s__Eggerthellaceae_bacterium | 11 (28.2) | 0.17 | 0.401 | | 0.880 |  | -0.23 | 0.263 | | 0.753 |
| s__Phocaeicola_plebeius | 17 (43.6) | 0.14 | 0.511 | | 0.880 |  | -0.16 | 0.442 | | 0.843 |
| s__Phocaeicola_vulgatus | 38 (97.4) | -0.15 | 0.463 | | 0.880 |  | 0.16 | 0.420 | | 0.841 |
| s__Paraprevotella_clara | 13 (33.3) | -0.13 | 0.523 | | 0.880 |  | 0.15 | 0.469 | | 0.850 |
| s__Parabacteroides_merdae | 28 (71.8) | 0.13 | 0.513 | | 0.880 |  | -0.19 | 0.361 | | 0.796 |
| s__GGB9581_SGB14999 | 32 (82.1) | 0.13 | 0.538 | | 0.880 |  | -0.12 | 0.556 | | 0.874 |
| s__Clostridia_bacterium_UC5_1_1D1 | 20 (51.3) | -0.13 | 0.526 | | 0.880 |  | 0.18 | 0.376 | | 0.821 |
| s__Clostridiaceae_bacterium_OM08_6BH | 20 (51.3) | 0.16 | 0.445 | | 0.880 |  | -0.19 | 0.349 | | 0.796 |
| s__Clostridiaceae_unclassified_SGB4769 | 11 (28.2) | -0.13 | 0.536 | | 0.880 |  | 0.10 | 0.629 | | 0.906 |
| s__Clostridium_sp_AF15_49 | 10 (25.6) | 0.13 | 0.527 | | 0.880 |  | -0.11 | 0.599 | | 0.901 |
| s__Anaerotignum_faecicola | 36 (92.3) | -0.17 | 0.401 | | 0.880 |  | 0.12 | 0.567 | | 0.879 |
| s__Blautia_obeum | 34 (87.2) | -0.14 | 0.502 | | 0.880 |  | 0.14 | 0.502 | | 0.862 |
| s__Blautia_SGB4815 | 21 (53.8) | 0.15 | 0.453 | | 0.880 |  | -0.17 | 0.415 | | 0.841 |
| s__Blautia_sp_AF19_10LB | 20 (51.3) | -0.15 | 0.461 | | 0.880 |  | 0.09 | 0.670 | | 0.944 |
| s__Faecalicatena_fissicatena | 23 (59) | 0.15 | 0.470 | | 0.880 |  | -0.17 | 0.404 | | 0.836 |
| s__Clostridium_scindens | 13 (33.3) | -0.15 | 0.456 | | 0.880 |  | 0.20 | 0.335 | | 0.795 |
| s__Clostridium_symbiosum | 13 (33.3) | -0.15 | 0.454 | | 0.880 |  | 0.17 | 0.400 | | 0.836 |
| s__Eubacterium_rectale | 31 (79.5) | 0.13 | 0.527 | | 0.880 |  | -0.19 | 0.356 | | 0.796 |
| s__Lachnospiraceae_bacterium | 39 (100) | 0.14 | 0.479 | | 0.880 |  | -0.13 | 0.535 | | 0.869 |
| s__Roseburia_intestinalis | 19 (48.7) | 0.17 | 0.420 | | 0.880 |  | -0.13 | 0.512 | | 0.862 |
| s__Dysosmobacter_sp_NSJ_60 | 27 (69.2) | -0.14 | 0.497 | | 0.880 |  | 0.20 | 0.332 | | 0.795 |
| s__Dysosmobacter_welbionis | 37 (94.9) | -0.13 | 0.521 | | 0.880 |  | 0.23 | 0.267 | | 0.753 |
| s__Oscillibacter_sp_ER4 | 26 (66.7) | -0.14 | 0.498 | | 0.880 |  | 0.14 | 0.508 | | 0.862 |
| s__Peptococcaceae_bacterium | 10 (25.6) | 0.13 | 0.520 | | 0.880 |  | -0.05 | 0.801 | | 0.986 |
| s__Agathobaculum_butyriciproducens | 38 (97.4) | 0.13 | 0.533 | | 0.880 |  | -0.11 | 0.585 | | 0.898 |
| s__Gemmiger_SGB15295 | 13 (33.3) | 0.15 | 0.462 | | 0.880 |  | -0.17 | 0.418 | | 0.841 |
| s__GGB9619_SGB15067 | 22 (56.4) | -0.16 | 0.433 | | 0.880 |  | 0.22 | 0.276 | | 0.758 |
| s__GGB9627_SGB15081 | 10 (25.6) | 0.17 | 0.411 | | 0.880 |  | -0.24 | 0.244 | | 0.740 |
| s__GGB9699_SGB15216 | 28 (71.8) | -0.14 | 0.481 | | 0.880 |  | 0.21 | 0.300 | | 0.790 |
| s__Hydrogeniiclostidium_mannosilyticum | 10 (25.6) | -0.16 | 0.435 | | 0.880 |  | 0.21 | 0.313 | | 0.790 |
| s__Allisonella_histaminiformans | 14 (35.9) | 0.14 | 0.486 | | 0.880 |  | -0.16 | 0.426 | | 0.841 |
| s__Fusobacterium_mortiferum | 11 (28.2) | 0.16 | 0.431 | | 0.880 |  | -0.08 | 0.694 | | 0.963 |
| s__Streptococcus_parasanguinis | 14 (35.9) | -0.12 | 0.550 | | 0.893 |  | 0.15 | 0.452 | | 0.850 |
| s__GGB9633_SGB15091 | 15 (38.5) | -0.12 | 0.573 | | 0.908 |  | 0.07 | 0.727 | | 0.967 |
| s__Holdemania_filiformis | 24 (61.5) | -0.12 | 0.565 | | 0.908 |  | 0.15 | 0.463 | | 0.850 |
| s__Firmicutes_bacterium_AF16_15 | 30 (76.9) | -0.12 | 0.569 | | 0.908 |  | 0.17 | 0.401 | | 0.836 |
| s__Streptococcus_salivarius | 36 (92.3) | 0.11 | 0.596 | | 0.921 |  | -0.16 | 0.433 | | 0.841 |
| s__GGB51441_SGB71759 | 15 (38.5) | 0.11 | 0.599 | | 0.921 |  | -0.11 | 0.589 | | 0.898 |
| s__Lacrimispora_celerecrescens | 16 (41) | 0.11 | 0.598 | | 0.921 |  | -0.11 | 0.598 | | 0.901 |
| s__GGB9615_SGB15052 | 14 (35.9) | -0.11 | 0.588 | | 0.921 |  | 0.10 | 0.630 | | 0.906 |
| s__Barnesiella_intestinihominis | 17 (43.6) | -0.11 | 0.607 | | 0.923 |  | 0.10 | 0.626 | | 0.906 |
| s__Butyricimonas_faecihominis | 10 (25.6) | -0.10 | 0.614 | | 0.923 |  | 0.12 | 0.558 | | 0.874 |
| s__Clostridium_innocuum | 11 (28.2) | -0.10 | 0.610 | | 0.923 |  | 0.14 | 0.509 | | 0.862 |
| s__Bacteroides_ovatus | 39 (100) | -0.09 | 0.659 | | 0.924 |  | 0.03 | 0.880 | | 0.999 |
| s__Alistipes_ihumii | 13 (33.3) | -0.09 | 0.646 | | 0.924 |  | 0.08 | 0.695 | | 0.963 |
| s__Alistipes_shahii | 29 (74.4) | -0.09 | 0.650 | | 0.924 |  | 0.09 | 0.666 | | 0.944 |
| s__Alistipes_sp_AF17_16 | 23 (59) | -0.10 | 0.642 | | 0.924 |  | 0.08 | 0.698 | | 0.963 |
| s__Eubacterium_ventriosum | 29 (74.4) | -0.09 | 0.664 | | 0.924 |  | 0.05 | 0.811 | | 0.986 |
| s__Blautia_stercoris | 18 (46.2) | 0.09 | 0.664 | | 0.924 |  | -0.16 | 0.435 | | 0.841 |
| s__Lachnospira_eligens | 33 (84.6) | 0.10 | 0.635 | | 0.924 |  | -0.07 | 0.733 | | 0.967 |
| s__Roseburia_inulinivorans | 33 (84.6) | 0.09 | 0.653 | | 0.924 |  | -0.06 | 0.785 | | 0.984 |
| s__Ruminococcus_bicirculans | 20 (51.3) | -0.09 | 0.645 | | 0.924 |  | 0.15 | 0.468 | | 0.850 |
| s__Dialister_hominis | 15 (38.5) | -0.10 | 0.623 | | 0.924 |  | 0.10 | 0.622 | | 0.906 |
| s__Klebsiella_pneumoniae | 19 (48.7) | -0.09 | 0.646 | | 0.924 |  | 0.13 | 0.542 | | 0.873 |
| s__GGB3571_SGB4778 | 17 (43.6) | -0.09 | 0.679 | | 0.938 |  | 0.08 | 0.708 | | 0.967 |
| s__Clostridium_sp_SN20 | 18 (46.2) | 0.08 | 0.687 | | 0.942 |  | -0.06 | 0.773 | | 0.984 |
| s__Bacteroides_eggerthii | 11 (28.2) | 0.06 | 0.768 | | 0.948 |  | -0.03 | 0.880 | | 0.999 |
| s__Alistipes_communis | 19 (48.7) | 0.07 | 0.719 | | 0.948 |  | 0.06 | 0.766 | | 0.984 |
| s__Alistipes_putredinis | 29 (74.4) | 0.07 | 0.719 | | 0.948 |  | 0.02 | 0.935 | | 0.999 |
| s__Clostridia_unclassified_SGB6276 | 11 (28.2) | 0.08 | 0.710 | | 0.948 |  | -0.14 | 0.481 | | 0.862 |
| s__GGB9758_SGB15368 | 12 (30.8) | -0.07 | 0.736 | | 0.948 |  | 0.13 | 0.514 | | 0.862 |
| s__Clostridium_sp_AF36_4 | 10 (25.6) | -0.06 | 0.771 | | 0.948 |  | 0.03 | 0.880 | | 0.999 |
| s__Dorea_longicatena | 33 (84.6) | -0.07 | 0.745 | | 0.948 |  | 0.03 | 0.896 | | 0.999 |
| s__Mediterraneibacter_glycyrrhizinilyticus | 11 (28.2) | 0.07 | 0.751 | | 0.948 |  | -0.02 | 0.932 | | 0.999 |
| s__Ruminococcus_gnavus | 19 (48.7) | -0.07 | 0.752 | | 0.948 |  | 0.10 | 0.636 | | 0.909 |
| s__Roseburia_sp_AF02_12 | 15 (38.5) | 0.06 | 0.756 | | 0.948 |  | -0.06 | 0.765 | | 0.984 |
| s__Candidatus_Cibiobacter_qucibialis | 30 (76.9) | 0.06 | 0.771 | | 0.948 |  | 0.01 | 0.946 | | 0.999 |
| s__GGB9614_SGB15049 | 31 (79.5) | 0.08 | 0.700 | | 0.948 |  | -0.05 | 0.814 | | 0.986 |
| s__GGB9615_SGB15053 | 30 (76.9) | -0.07 | 0.746 | | 0.948 |  | 0.05 | 0.820 | | 0.986 |
| s__GGB9730_SGB15291 | 20 (51.3) | 0.06 | 0.758 | | 0.948 |  | -0.12 | 0.560 | | 0.874 |
| s__Eubacterium_siraeum | 12 (30.8) | -0.08 | 0.705 | | 0.948 |  | -0.05 | 0.810 | | 0.986 |
| s__Ruminococcaceae_unclassified_SGB15234 | 10 (25.6) | -0.06 | 0.775 | | 0.948 |  | 0.01 | 0.979 | | 0.999 |
| s__Parasutterella_SGB9260 | 12 (30.8) | 0.06 | 0.755 | | 0.948 |  | 0.06 | 0.780 | | 0.984 |
| s__Akkermansia_muciniphila | 13 (33.3) | -0.07 | 0.729 | | 0.948 |  | 0.01 | 0.977 | | 0.999 |
| s__Phocaeicola_massiliensis | 18 (46.2) | 0.06 | 0.781 | | 0.949 |  | -0.02 | 0.907 | | 0.999 |
| s__Rothia_mucilaginosa | 13 (33.3) | 0.04 | 0.839 | | 0.950 |  | -0.07 | 0.732 | | 0.967 |
| s__Adlercreutzia_equolifaciens | 26 (66.7) | 0.05 | 0.800 | | 0.950 |  | 0.04 | 0.829 | | 0.986 |
| s__Bacteroides_xylanisolvens | 34 (87.2) | 0.04 | 0.856 | | 0.950 |  | -0.07 | 0.732 | | 0.967 |
| s__Butyricimonas_virosa | 24 (61.5) | -0.04 | 0.838 | | 0.950 |  | 0.11 | 0.609 | | 0.906 |
| s__Candidatus_Saccharibacteria_unclassified_SGB19850 | 12 (30.8) | 0.04 | 0.856 | | 0.950 |  | 0.01 | 0.948 | | 0.999 |
| s__GGB35068_SGB47850 | 11 (28.2) | 0.05 | 0.822 | | 0.950 |  | -0.10 | 0.623 | | 0.906 |
| s__Clostridia_unclassified_SGB4121 | 25 (64.1) | 0.04 | 0.832 | | 0.950 |  | -0.01 | 0.963 | | 0.999 |
| s__Eubacteriaceae_bacterium | 23 (59) | 0.04 | 0.851 | | 0.950 |  | -0.05 | 0.795 | | 0.986 |
| s__Blautia_faecis | 38 (97.4) | -0.05 | 0.807 | | 0.950 |  | -0.01 | 0.963 | | 0.999 |
| s__Lachnospira_pectinoschiza | 17 (43.6) | 0.05 | 0.805 | | 0.950 |  | -0.02 | 0.939 | | 0.999 |
| s__Lachnospiraceae_bacterium_NSJ_29 | 10 (25.6) | -0.05 | 0.797 | | 0.950 |  | 0.00 | 0.995 | | 0.999 |
| s__GGB9632_SGB15089 | 33 (84.6) | 0.04 | 0.828 | | 0.950 |  | 0.01 | 0.952 | | 0.999 |
| s__Ruminococcus_bromii | 21 (53.8) | 0.04 | 0.846 | | 0.950 |  | -0.07 | 0.720 | | 0.967 |
| s__Faecalibacillus_intestinalis | 34 (87.2) | 0.04 | 0.828 | | 0.950 |  | -0.07 | 0.734 | | 0.967 |
| s__GGB41458_SGB58520 | 10 (25.6) | -0.04 | 0.849 | | 0.950 |  | 0.03 | 0.884 | | 0.999 |
| s__Escherichia_coli | 29 (74.4) | 0.04 | 0.846 | | 0.950 |  | -0.02 | 0.914 | | 0.999 |
| s__Raoultibacter_timonensis | 11 (28.2) | 0.03 | 0.882 | | 0.954 |  | -0.02 | 0.927 | | 0.999 |
| s__Odoribacter_splanchnicus | 28 (71.8) | 0.03 | 0.898 | | 0.954 |  | -0.05 | 0.824 | | 0.986 |
| s__GGB2982_SGB3964 | 12 (30.8) | 0.02 | 0.905 | | 0.954 |  | -0.03 | 0.893 | | 0.999 |
| s__Clostridiaceae_bacterium | 39 (100) | -0.03 | 0.894 | | 0.954 |  | 0.04 | 0.838 | | 0.990 |
| s__Clostridium_sp_AF34_10BH | 37 (94.9) | 0.03 | 0.871 | | 0.954 |  | 0.00 | 0.996 | | 0.999 |
| s__Anaerosacchariphilus_sp_NSJ_68 | 12 (30.8) | 0.03 | 0.880 | | 0.954 |  | -0.14 | 0.506 | | 0.862 |
| s__Roseburia_faecis | 25 (64.1) | -0.03 | 0.900 | | 0.954 |  | 0.00 | 0.982 | | 0.999 |
| s__Romboutsia_timonensis | 18 (46.2) | 0.02 | 0.905 | | 0.954 |  | -0.06 | 0.761 | | 0.984 |
| s__Gemmiger_formicilis | 25 (64.1) | -0.02 | 0.909 | | 0.954 |  | -0.03 | 0.890 | | 0.999 |
| s__GGB9705_SGB15225 | 12 (30.8) | -0.02 | 0.911 | | 0.954 |  | 0.03 | 0.868 | | 0.999 |
| s__Negativibacillus_massiliensis | 16 (41) | 0.03 | 0.898 | | 0.954 |  | 0.00 | 0.988 | | 0.999 |
| s__Anaerostipes_hadrus | 37 (94.9) | 0.02 | 0.919 | | 0.957 |  | -0.04 | 0.831 | | 0.986 |
| s__Eubacterium_sp_AF34_35BH | 14 (35.9) | -0.02 | 0.936 | | 0.970 |  | -0.01 | 0.955 | | 0.999 |
| s__Phascolarctobacterium_faecium | 33 (84.6) | -0.01 | 0.946 | | 0.974 |  | 0.00 | 0.999 | | 0.999 |
| s__Bacteroides_stercoris | 32 (82.1) | 0.01 | 0.962 | | 0.976 |  | -0.06 | 0.756 | | 0.984 |
| s__Parabacteroides_goldsteinii | 14 (35.9) | 0.01 | 0.961 | | 0.976 |  | -0.03 | 0.899 | | 0.999 |
| s__Clostridium_leptum | 26 (66.7) | 0.01 | 0.953 | | 0.976 |  | -0.01 | 0.964 | | 0.999 |
| s__Bifidobacterium_longum | 31 (79.5) | -0.01 | 0.969 | | 0.979 |  | -0.01 | 0.948 | | 0.999 |
| s__Clostridium_SGB4751 | 14 (35.9) | -0.01 | 0.974 | | 0.979 |  | -0.06 | 0.784 | | 0.984 |
| s__Blautia_massiliensis | 32 (82.1) | 0.00 | 0.988 | | 0.988 |  | 0.00 | 0.997 | | 0.999 |

**Table S15. Correlations between the abundances of 1-month relative abundances of bacteria and sVNT levels at 6 months p.v. in the BNT162b2 group.**

|  |  | 6-month sVNT | | |  | Relative drop (from 1 month to 6 months) | | |
| --- | --- | --- | --- | --- | --- | --- | --- | --- |
| Species | N (%) | Spearman's Rho | P value | FDR |  | Spearman's Rho | P value | FDR |
| s__Parasutterella_excrementihominis | 69 (58.5) | 0.21 | 0.033 | 0.982 |  | -0.21 | 0.034 | 0.976 |
| s__Alistipes_ihumii | 36 (30.5) | -0.20 | 0.043 | 0.982 |  | 0.18 | 0.078 | 0.976 |
| s__Butyricimonas_paravirosa | 35 (29.7) | -0.20 | 0.047 | 0.982 |  | 0.19 | 0.056 | 0.976 |
| s__Ruminococcaceae_bacterium_AM07_15 | 45 (38.1) | -0.18 | 0.072 | 0.982 |  | 0.18 | 0.078 | 0.976 |
| s__Clostridium_fessum | 97 (82.2) | 0.18 | 0.078 | 0.982 |  | -0.16 | 0.107 | 0.976 |
| s__Bacteroides_salyersiae | 38 (32.2) | -0.17 | 0.086 | 0.982 |  | 0.17 | 0.099 | 0.976 |
| s__Lachnospiraceae_bacterium_BX3 | 34 (28.8) | -0.17 | 0.090 | 0.982 |  | 0.18 | 0.072 | 0.976 |
| s__Butyricicoccus_sp_AM29_23AC | 37 (31.4) | 0.17 | 0.095 | 0.982 |  | -0.15 | 0.131 | 0.976 |
| s__Blautia_stercoris | 40 (33.9) | 0.17 | 0.099 | 0.982 |  | -0.17 | 0.094 | 0.976 |
| s__Dorea_sp_AF36_15AT | 45 (38.1) | 0.16 | 0.113 | 0.982 |  | -0.16 | 0.101 | 0.976 |
| s__Clostridium_sp_AM22_11AC | 88 (74.6) | 0.16 | 0.116 | 0.982 |  | -0.14 | 0.165 | 0.976 |
| s__Streptococcus_parasanguinis | 46 (39) | 0.15 | 0.124 | 0.982 |  | -0.16 | 0.114 | 0.976 |
| s__Enterocloster_aldensis | 39 (33.1) | 0.15 | 0.124 | 0.982 |  | -0.15 | 0.144 | 0.976 |
| s__Bifidobacterium_adolescentis | 88 (74.6) | 0.15 | 0.125 | 0.982 |  | -0.15 | 0.123 | 0.976 |
| s__Alistipes_finegoldii | 44 (37.3) | 0.15 | 0.134 | 0.982 |  | -0.16 | 0.107 | 0.976 |
| s__Parabacteroides_distasonis | 117 (99.2) | 0.15 | 0.139 | 0.982 |  | -0.17 | 0.095 | 0.976 |
| s__GGB9627_SGB15081 | 41 (34.7) | -0.14 | 0.149 | 0.982 |  | 0.14 | 0.169 | 0.976 |
| s__GGB9581_SGB14999 | 92 (78) | -0.14 | 0.150 | 0.982 |  | 0.14 | 0.162 | 0.976 |
| s__GGB2982_SGB3964 | 47 (39.8) | 0.14 | 0.150 | 0.982 |  | -0.16 | 0.105 | 0.976 |
| s__Dorea_longicatena | 96 (81.4) | 0.14 | 0.155 | 0.982 |  | -0.14 | 0.176 | 0.976 |
| s__Anaerobutyricum_hallii | 106 (89.8) | -0.14 | 0.160 | 0.982 |  | 0.14 | 0.155 | 0.976 |
| s__Eggerthellaceae_bacterium | 34 (28.8) | -0.14 | 0.176 | 0.982 |  | 0.12 | 0.228 | 0.976 |
| s__Lacrimispora_amygdalina | 101 (85.6) | 0.13 | 0.184 | 0.982 |  | -0.13 | 0.200 | 0.976 |
| s__Streptococcus_salivarius | 94 (79.7) | 0.13 | 0.184 | 0.982 |  | -0.14 | 0.177 | 0.976 |
| s__Megamonas_funiformis | 37 (31.4) | -0.13 | 0.187 | 0.982 |  | 0.13 | 0.207 | 0.976 |
| s__Coprococcus_comes | 79 (66.9) | 0.13 | 0.199 | 0.982 |  | -0.12 | 0.213 | 0.976 |
| s__Bacteroides_eggerthii | 31 (26.3) | 0.13 | 0.204 | 0.982 |  | -0.13 | 0.198 | 0.976 |
| s__Alistipes_communis | 58 (49.2) | -0.13 | 0.210 | 0.982 |  | 0.12 | 0.230 | 0.976 |
| s__Bifidobacterium_longum | 102 (86.4) | -0.13 | 0.210 | 0.982 |  | 0.13 | 0.179 | 0.976 |
| s__Lachnospira_sp_NSJ_43 | 47 (39.8) | 0.12 | 0.216 | 0.982 |  | -0.12 | 0.230 | 0.976 |
| s__Holdemania_filiformis | 63 (53.4) | -0.12 | 0.222 | 0.982 |  | 0.12 | 0.214 | 0.976 |
| s__Roseburia_sp_AF02_12 | 38 (32.2) | -0.12 | 0.224 | 0.982 |  | 0.13 | 0.188 | 0.976 |
| s__Clostridium_SGB4751 | 45 (38.1) | 0.12 | 0.227 | 0.982 |  | -0.12 | 0.227 | 0.976 |
| s__Bacteroides_thetaiotaomicron | 115 (97.5) | 0.12 | 0.233 | 0.982 |  | -0.10 | 0.318 | 0.976 |
| s__GGB9707_SGB15229 | 37 (31.4) | 0.12 | 0.234 | 0.982 |  | -0.13 | 0.211 | 0.976 |
| s__Blautia_wexlerae | 116 (98.3) | 0.12 | 0.247 | 0.982 |  | -0.10 | 0.326 | 0.976 |
| s__Clostridium_sp_AF27_2AA | 41 (34.7) | 0.11 | 0.252 | 0.982 |  | -0.11 | 0.266 | 0.976 |
| s__Clostridium_leptum | 69 (58.5) | 0.11 | 0.257 | 0.982 |  | -0.12 | 0.243 | 0.976 |
| s__Clostridium_innocuum | 34 (28.8) | 0.11 | 0.273 | 0.982 |  | -0.11 | 0.262 | 0.976 |
| s__Phocaeicola_vulgatus | 100 (84.7) | 0.11 | 0.274 | 0.982 |  | -0.11 | 0.259 | 0.976 |
| s__Eubacterium_ventriosum | 83 (70.3) | -0.11 | 0.275 | 0.982 |  | 0.11 | 0.265 | 0.976 |
| s__Alistipes_onderdonkii | 70 (59.3) | -0.11 | 0.277 | 0.982 |  | 0.10 | 0.323 | 0.976 |
| s__GGB9705_SGB15225 | 34 (28.8) | -0.11 | 0.278 | 0.982 |  | 0.11 | 0.270 | 0.976 |
| s__Clostridium_sp_AM33_3 | 68 (57.6) | -0.11 | 0.284 | 0.982 |  | 0.12 | 0.251 | 0.976 |
| s__GGB51441_SGB71759 | 33 (28) | 0.11 | 0.285 | 0.982 |  | -0.11 | 0.292 | 0.976 |
| s__Ruminococcus_SGB4421 | 31 (26.3) | -0.11 | 0.289 | 0.982 |  | 0.11 | 0.295 | 0.976 |
| s__Clostridium_sp_AF20_17LB | 53 (44.9) | 0.11 | 0.291 | 0.982 |  | -0.09 | 0.355 | 0.976 |
| s__Alistipes_indistinctus | 70 (59.3) | 0.10 | 0.303 | 0.982 |  | -0.12 | 0.242 | 0.976 |
| s__GGB9557_SGB14966 | 41 (34.7) | -0.10 | 0.305 | 0.982 |  | 0.10 | 0.314 | 0.976 |
| s__Clostridium_sp_AF36_4 | 32 (27.1) | 0.10 | 0.312 | 0.982 |  | -0.09 | 0.363 | 0.976 |
| s__Hungatella_hathewayi | 51 (43.2) | -0.10 | 0.314 | 0.982 |  | 0.09 | 0.363 | 0.976 |
| s__Allisonella_histaminiformans | 38 (32.2) | -0.10 | 0.324 | 0.982 |  | 0.10 | 0.307 | 0.976 |
| s__Clostridiaceae_bacterium | 109 (92.4) | 0.10 | 0.332 | 0.982 |  | -0.08 | 0.443 | 0.976 |
| s__Clostridium_phoceensis | 100 (84.7) | -0.10 | 0.335 | 0.982 |  | 0.10 | 0.341 | 0.976 |
| s__Clostridiales_bacterium_KLE1615 | 102 (86.4) | 0.10 | 0.335 | 0.982 |  | -0.08 | 0.450 | 0.976 |
| s__Clostridium_sp_SN20 | 54 (45.8) | -0.10 | 0.337 | 0.982 |  | 0.10 | 0.317 | 0.976 |
| s__Collinsella_aerofaciens | 98 (83.1) | -0.10 | 0.342 | 0.982 |  | 0.08 | 0.403 | 0.976 |
| s__Anaerotignum_faecicola | 80 (67.8) | -0.09 | 0.347 | 0.982 |  | 0.09 | 0.382 | 0.976 |
| s__GGB9730_SGB15291 | 55 (46.6) | -0.09 | 0.351 | 0.982 |  | 0.08 | 0.428 | 0.976 |
| s__Lacrimispora_celerecrescens | 43 (36.4) | -0.09 | 0.352 | 0.982 |  | 0.09 | 0.389 | 0.976 |
| s__GGB58485_SGB80143 | 32 (27.1) | 0.09 | 0.358 | 0.982 |  | -0.10 | 0.314 | 0.976 |
| s__GGB9619_SGB15067 | 71 (60.2) | 0.09 | 0.366 | 0.982 |  | -0.09 | 0.375 | 0.976 |
| s__Clostridium_sp_AM49_4BH | 35 (29.7) | 0.09 | 0.368 | 0.982 |  | -0.10 | 0.329 | 0.976 |
| s__Blautia_faecis | 114 (96.6) | 0.09 | 0.370 | 0.982 |  | -0.08 | 0.446 | 0.976 |
| s__Eubacterium_rectale | 90 (76.3) | 0.09 | 0.372 | 0.982 |  | -0.08 | 0.428 | 0.976 |
| s__Bacteroides_nordii | 68 (57.6) | 0.09 | 0.374 | 0.982 |  | -0.09 | 0.375 | 0.976 |
| s__Ruminococcus_lactaris | 38 (32.2) | 0.09 | 0.396 | 0.982 |  | -0.07 | 0.507 | 0.976 |
| s__Clostridiales_bacterium | 76 (64.4) | -0.08 | 0.409 | 0.982 |  | 0.07 | 0.481 | 0.976 |
| s__Bacteroides_ovatus | 108 (91.5) | 0.08 | 0.417 | 0.982 |  | -0.08 | 0.415 | 0.976 |
| s__GGB9633_SGB15091 | 39 (33.1) | -0.08 | 0.417 | 0.982 |  | 0.08 | 0.412 | 0.976 |
| s__Evtepia_gabavorous | 46 (39) | 0.08 | 0.419 | 0.982 |  | -0.10 | 0.326 | 0.976 |
| s__Firmicutes_bacterium_AF16_15 | 82 (69.5) | -0.08 | 0.420 | 0.982 |  | 0.07 | 0.464 | 0.976 |
| s__Veillonella_parvula | 44 (37.3) | 0.08 | 0.429 | 0.982 |  | -0.08 | 0.431 | 0.976 |
| s__Clostridium_sp_AF15_49 | 32 (27.1) | 0.08 | 0.432 | 0.982 |  | -0.09 | 0.396 | 0.976 |
| s__Haemophilus_parainfluenzae | 59 (50) | 0.08 | 0.440 | 0.982 |  | -0.08 | 0.431 | 0.976 |
| s__Clostridium_symbiosum | 38 (32.2) | 0.08 | 0.448 | 0.982 |  | -0.07 | 0.485 | 0.976 |
| s__Acidaminococcus_intestini | 31 (26.3) | -0.08 | 0.449 | 0.982 |  | 0.06 | 0.560 | 0.976 |
| s__Alistipes_shahii | 78 (66.1) | -0.08 | 0.452 | 0.982 |  | 0.06 | 0.536 | 0.976 |
| s__Dysosmobacter_sp_NSJ_60 | 73 (61.9) | -0.07 | 0.461 | 0.982 |  | 0.07 | 0.480 | 0.976 |
| s__Christensenellaceae_bacterium | 53 (44.9) | 0.07 | 0.468 | 0.982 |  | -0.07 | 0.477 | 0.976 |
| s__Roseburia_hominis | 87 (73.7) | 0.07 | 0.468 | 0.982 |  | -0.07 | 0.458 | 0.976 |
| s__Alistipes_sp_AF17_16 | 61 (51.7) | 0.07 | 0.478 | 0.982 |  | -0.09 | 0.394 | 0.976 |
| s__Klebsiella_pneumoniae | 77 (65.3) | -0.07 | 0.486 | 0.982 |  | 0.05 | 0.622 | 0.976 |
| s__Blautia_obeum | 100 (84.7) | -0.07 | 0.492 | 0.982 |  | 0.05 | 0.596 | 0.976 |
| s__Clostridia_bacterium | 106 (89.8) | 0.07 | 0.503 | 0.982 |  | -0.06 | 0.526 | 0.976 |
| s__Blautia_massiliensis | 99 (83.9) | -0.07 | 0.504 | 0.982 |  | 0.06 | 0.519 | 0.976 |
| s__Enterocloster_bolteae | 70 (59.3) | 0.07 | 0.507 | 0.982 |  | -0.06 | 0.526 | 0.976 |
| s__Parabacteroides_goldsteinii | 58 (49.2) | -0.07 | 0.511 | 0.982 |  | 0.07 | 0.510 | 0.976 |
| s__Clostridiales_unclassified_SGB15145 | 45 (38.1) | -0.07 | 0.516 | 0.982 |  | 0.06 | 0.583 | 0.976 |
| s__Mesosutterella_multiformis | 43 (36.4) | 0.07 | 0.517 | 0.982 |  | -0.07 | 0.467 | 0.976 |
| s__Bacteroides_stercoris | 74 (62.7) | 0.06 | 0.525 | 0.982 |  | -0.09 | 0.391 | 0.976 |
| s__Lachnospira_eligens | 88 (74.6) | -0.06 | 0.526 | 0.982 |  | 0.06 | 0.555 | 0.976 |
| s__GGB3571_SGB4778 | 54 (45.8) | -0.06 | 0.527 | 0.982 |  | 0.06 | 0.530 | 0.976 |
| s__Faecalibacillus_intestinalis | 89 (75.4) | -0.06 | 0.527 | 0.982 |  | 0.07 | 0.497 | 0.976 |
| s__Bacteroides_caccae | 84 (71.2) | 0.06 | 0.540 | 0.982 |  | -0.06 | 0.522 | 0.976 |
| s__GGB9758_SGB15368 | 38 (32.2) | 0.06 | 0.545 | 0.982 |  | -0.07 | 0.477 | 0.976 |
| s__Candidatus_Avimicrobium_caecorum | 77 (65.3) | -0.06 | 0.548 | 0.982 |  | 0.07 | 0.508 | 0.976 |
| s__Lachnospiraceae_bacterium_WCA3_601_WT_6H | 82 (69.5) | -0.06 | 0.553 | 0.982 |  | 0.06 | 0.582 | 0.976 |
| s__Phocaeicola_dorei | 67 (56.8) | -0.06 | 0.554 | 0.982 |  | 0.06 | 0.531 | 0.976 |
| s__Bacteroides_cellulosilyticus | 77 (65.3) | 0.06 | 0.557 | 0.982 |  | -0.05 | 0.597 | 0.976 |
| s__Intestinibacter_bartlettii | 32 (27.1) | 0.06 | 0.559 | 0.982 |  | -0.06 | 0.529 | 0.976 |
| s__Clostridia_unclassified_SGB4447 | 47 (39.8) | -0.06 | 0.560 | 0.982 |  | 0.07 | 0.496 | 0.976 |
| s__GGB9615_SGB15053 | 69 (58.5) | -0.06 | 0.562 | 0.982 |  | 0.05 | 0.618 | 0.976 |
| s__Odoribacter_splanchnicus | 83 (70.3) | 0.06 | 0.566 | 0.982 |  | -0.07 | 0.514 | 0.976 |
| s__Bacteroides_xylanisolvens | 94 (79.7) | -0.06 | 0.567 | 0.982 |  | 0.06 | 0.533 | 0.976 |
| s__Gemmiger_formicilis | 82 (69.5) | -0.06 | 0.568 | 0.982 |  | 0.05 | 0.589 | 0.976 |
| s__Ruminococcaceae_unclassified_SGB15260 | 44 (37.3) | -0.06 | 0.576 | 0.982 |  | 0.06 | 0.523 | 0.976 |
| s__Clostridium_scindens | 37 (31.4) | -0.05 | 0.587 | 0.982 |  | 0.07 | 0.515 | 0.976 |
| s__Eubacteriaceae_bacterium | 63 (53.4) | 0.05 | 0.589 | 0.982 |  | -0.04 | 0.678 | 0.978 |
| s__Clostridia_unclassified_SGB4121 | 93 (78.8) | -0.05 | 0.601 | 0.982 |  | 0.06 | 0.549 | 0.976 |
| s__Lachnospiraceae_bacterium | 115 (97.5) | 0.05 | 0.603 | 0.982 |  | -0.04 | 0.686 | 0.978 |
| s__Ruthenibacterium_lactatiformans | 110 (93.2) | -0.05 | 0.607 | 0.982 |  | 0.06 | 0.568 | 0.976 |
| s__Anaeromassilibacillus_sp_An250 | 44 (37.3) | 0.05 | 0.607 | 0.982 |  | -0.04 | 0.683 | 0.978 |
| s__GGB9614_SGB15049 | 81 (68.6) | -0.05 | 0.608 | 0.982 |  | 0.05 | 0.629 | 0.976 |
| s__Enterocloster_asparagiformis | 31 (26.3) | -0.05 | 0.610 | 0.982 |  | 0.05 | 0.608 | 0.976 |
| s__Agathobaculum_butyriciproducens | 109 (92.4) | -0.05 | 0.617 | 0.982 |  | 0.07 | 0.488 | 0.976 |
| s__Ruminococcus_bicirculans | 69 (58.5) | 0.05 | 0.618 | 0.982 |  | -0.05 | 0.621 | 0.976 |
| s__Lachnospiraceae_unclassified_SGB4894 | 30 (25.4) | 0.05 | 0.618 | 0.982 |  | -0.06 | 0.523 | 0.976 |
| s__Faecalicatena_fissicatena | 64 (54.2) | 0.05 | 0.618 | 0.982 |  | -0.06 | 0.580 | 0.976 |
| s__Bacteroides_uniformis | 115 (97.5) | -0.05 | 0.631 | 0.982 |  | 0.04 | 0.726 | 0.978 |
| s__Phocaeicola_plebeius | 35 (29.7) | -0.05 | 0.631 | 0.982 |  | 0.04 | 0.720 | 0.978 |
| s__Bilophila_wadsworthia | 110 (93.2) | 0.05 | 0.633 | 0.982 |  | -0.06 | 0.541 | 0.976 |
| s__Clostridium_sp_AM42_4 | 46 (39) | 0.05 | 0.635 | 0.982 |  | -0.05 | 0.646 | 0.978 |
| s__Roseburia_inulinivorans | 104 (88.1) | 0.05 | 0.642 | 0.982 |  | -0.04 | 0.708 | 0.978 |
| s__Enterocloster_citroniae | 68 (57.6) | 0.05 | 0.642 | 0.982 |  | -0.05 | 0.625 | 0.976 |
| s__Ruminococcaceae_bacterium | 83 (70.3) | -0.05 | 0.644 | 0.982 |  | 0.05 | 0.604 | 0.976 |
| s__Blautia_glucerasea | 52 (44.1) | -0.05 | 0.647 | 0.982 |  | 0.07 | 0.460 | 0.976 |
| s__Barnesiella_intestinihominis | 38 (32.2) | -0.05 | 0.651 | 0.982 |  | 0.03 | 0.743 | 0.982 |
| s__Fusicatenibacter_saccharivorans | 107 (90.7) | 0.05 | 0.653 | 0.982 |  | -0.03 | 0.729 | 0.978 |
| s__Phocea_massiliensis | 56 (47.5) | 0.04 | 0.658 | 0.982 |  | -0.06 | 0.523 | 0.976 |
| s__Ruminococcus_torques | 98 (83.1) | 0.04 | 0.664 | 0.982 |  | -0.04 | 0.723 | 0.978 |
| s__GGB35068_SGB47850 | 39 (33.1) | -0.04 | 0.670 | 0.982 |  | 0.04 | 0.671 | 0.978 |
| s__Escherichia_coli | 106 (89.8) | 0.04 | 0.679 | 0.982 |  | -0.05 | 0.601 | 0.976 |
| s__Coprococcus_eutactus | 35 (29.7) | 0.04 | 0.686 | 0.982 |  | -0.05 | 0.627 | 0.976 |
| s__Oscillibacter_sp_ER4 | 70 (59.3) | -0.04 | 0.687 | 0.982 |  | 0.04 | 0.709 | 0.978 |
| s__Eubacterium_siraeum | 31 (26.3) | -0.04 | 0.693 | 0.982 |  | 0.04 | 0.662 | 0.978 |
| s__Parabacteroides_merdae | 72 (61) | -0.04 | 0.698 | 0.982 |  | 0.03 | 0.755 | 0.983 |
| s__GGB9644_SGB15121 | 34 (28.8) | -0.04 | 0.705 | 0.982 |  | 0.05 | 0.588 | 0.976 |
| s__GGB9699_SGB15216 | 77 (65.3) | -0.04 | 0.715 | 0.982 |  | 0.03 | 0.729 | 0.978 |
| s__Alistipes_putredinis | 73 (61.9) | 0.04 | 0.717 | 0.982 |  | -0.05 | 0.601 | 0.976 |
| s__Clostridium_sp_AT4 | 31 (26.3) | -0.04 | 0.719 | 0.982 |  | 0.04 | 0.667 | 0.978 |
| s__Roseburia_faecis | 91 (77.1) | 0.04 | 0.723 | 0.982 |  | -0.04 | 0.717 | 0.978 |
| s__Dorea_sp_AF24_7LB | 34 (28.8) | -0.03 | 0.756 | 0.982 |  | 0.02 | 0.836 | 0.987 |
| s__Ruminococcaceae_unclassified_SGB15265 | 55 (46.6) | 0.03 | 0.759 | 0.982 |  | -0.03 | 0.731 | 0.978 |
| s__Intestinimonas_butyriciproducens | 48 (40.7) | -0.03 | 0.767 | 0.982 |  | 0.03 | 0.754 | 0.983 |
| s__Roseburia_intestinalis | 58 (49.2) | 0.03 | 0.768 | 0.982 |  | -0.01 | 0.912 | 0.987 |
| s__Faecalibacterium_SGB15346 | 85 (72) | -0.03 | 0.777 | 0.982 |  | 0.04 | 0.723 | 0.978 |
| s__Romboutsia_timonensis | 62 (52.5) | 0.03 | 0.782 | 0.982 |  | -0.04 | 0.727 | 0.978 |
| s__Dorea_formicigenerans | 89 (75.4) | 0.03 | 0.782 | 0.982 |  | -0.01 | 0.926 | 0.987 |
| s__Clostridium_sp_AF34_10BH | 107 (90.7) | -0.03 | 0.787 | 0.982 |  | 0.01 | 0.890 | 0.987 |
| s__Bittarella_massiliensis | 32 (27.1) | 0.03 | 0.796 | 0.982 |  | -0.01 | 0.945 | 0.987 |
| s__Ruminococcus_bromii | 57 (48.3) | -0.03 | 0.801 | 0.982 |  | 0.04 | 0.724 | 0.978 |
| s__Streptococcus_sp_A12 | 45 (38.1) | -0.02 | 0.808 | 0.982 |  | 0.04 | 0.708 | 0.978 |
| s__Clostridia_bacterium_UC5_1_1D1 | 89 (75.4) | 0.02 | 0.810 | 0.982 |  | -0.01 | 0.940 | 0.987 |
| s__GGB9602_SGB15031 | 31 (26.3) | 0.02 | 0.817 | 0.982 |  | -0.03 | 0.770 | 0.987 |
| s__Candidatus_Pararuminococcus_gallinarum | 32 (27.1) | -0.02 | 0.821 | 0.982 |  | 0.01 | 0.893 | 0.987 |
| s__Adlercreutzia_equolifaciens | 81 (68.6) | -0.02 | 0.826 | 0.982 |  | 0.03 | 0.790 | 0.987 |
| s__GGB3746_SGB5089 | 68 (57.6) | -0.02 | 0.834 | 0.982 |  | 0.03 | 0.767 | 0.987 |
| s__Phascolarctobacterium_faecium | 100 (84.7) | 0.02 | 0.835 | 0.982 |  | -0.03 | 0.776 | 0.987 |
| s__Coprococcus_catus | 73 (61.9) | 0.02 | 0.855 | 0.982 |  | 0.00 | 0.977 | 0.987 |
| s__Ruminococcus_gnavus | 78 (66.1) | -0.02 | 0.856 | 0.982 |  | 0.02 | 0.830 | 0.987 |
| s__Bacteroides_fragilis | 64 (54.2) | 0.02 | 0.857 | 0.982 |  | -0.02 | 0.859 | 0.987 |
| s__Blautia_sp_AF19_10LB | 65 (55.1) | -0.02 | 0.866 | 0.982 |  | 0.01 | 0.888 | 0.987 |
| s__Candidatus_Allochristensenella_caecavium | 32 (27.1) | -0.02 | 0.870 | 0.982 |  | 0.02 | 0.858 | 0.987 |
| s__Phocaeicola_massiliensis | 41 (34.7) | -0.02 | 0.870 | 0.982 |  | 0.01 | 0.954 | 0.987 |
| s__Sutterella_wadsworthensis | 52 (44.1) | -0.02 | 0.871 | 0.982 |  | 0.03 | 0.744 | 0.982 |
| s__Flavonifractor_plautii | 116 (98.3) | 0.02 | 0.872 | 0.982 |  | 0.00 | 0.987 | 0.992 |
| s__Ruminococcaceae_unclassified_SGB15234 | 34 (28.8) | -0.02 | 0.873 | 0.982 |  | 0.01 | 0.926 | 0.987 |
| s__Eubacterium_ramulus | 74 (62.7) | -0.02 | 0.874 | 0.982 |  | 0.01 | 0.937 | 0.987 |
| s__Butyricimonas_virosa | 65 (55.1) | -0.02 | 0.876 | 0.982 |  | 0.02 | 0.876 | 0.987 |
| s__Eggerthella_lenta | 69 (58.5) | -0.02 | 0.879 | 0.982 |  | 0.02 | 0.825 | 0.987 |
| s__Dysosmobacter_welbionis | 106 (89.8) | -0.02 | 0.881 | 0.982 |  | 0.02 | 0.833 | 0.987 |
| s__Dialister_hominis | 58 (49.2) | 0.01 | 0.889 | 0.982 |  | -0.02 | 0.810 | 0.987 |
| s__Mediterraneibacter_glycyrrhizinilyticus | 36 (30.5) | 0.01 | 0.892 | 0.982 |  | -0.01 | 0.903 | 0.987 |
| s__Gordonibacter_pamelaeae | 38 (32.2) | 0.01 | 0.895 | 0.982 |  | 0.00 | 0.965 | 0.987 |
| s__Bacteroides_finegoldii | 45 (38.1) | -0.01 | 0.897 | 0.982 |  | 0.00 | 0.972 | 0.987 |
| s__Bacteroides_intestinalis | 66 (55.9) | 0.01 | 0.899 | 0.982 |  | -0.02 | 0.850 | 0.987 |
| s__GGB9667_SGB15164 | 32 (27.1) | 0.01 | 0.901 | 0.982 |  | -0.02 | 0.832 | 0.987 |
| s__Akkermansia_muciniphila | 39 (33.1) | 0.01 | 0.924 | 0.989 |  | -0.01 | 0.953 | 0.987 |
| s__Lachnospira_SGB5076 | 32 (27.1) | -0.01 | 0.934 | 0.989 |  | 0.02 | 0.820 | 0.987 |
| s__Candidatus_Cibiobacter_qucibialis | 83 (70.3) | 0.01 | 0.938 | 0.989 |  | 0.00 | 0.965 | 0.987 |
| s__GGB9632_SGB15089 | 92 (78) | -0.01 | 0.940 | 0.989 |  | 0.02 | 0.879 | 0.987 |
| s__Anaerotruncus_colihominis | 57 (48.3) | 0.01 | 0.944 | 0.989 |  | 0.01 | 0.947 | 0.987 |
| s__Anaerotruncus_rubiinfantis | 32 (27.1) | -0.01 | 0.946 | 0.989 |  | 0.00 | 0.974 | 0.987 |
| s__Negativibacillus_massiliensis | 55 (46.6) | 0.01 | 0.949 | 0.989 |  | 0.01 | 0.906 | 0.987 |
| s__Faecalibacterium_prausnitzii | 112 (94.9) | -0.01 | 0.953 | 0.989 |  | 0.02 | 0.815 | 0.987 |
| s__Clostridiaceae_bacterium_OM08_6BH | 57 (48.3) | 0.01 | 0.958 | 0.989 |  | 0.00 | 0.975 | 0.987 |
| s__Rothia_mucilaginosa | 31 (26.3) | -0.01 | 0.958 | 0.989 |  | 0.01 | 0.917 | 0.987 |
| s__Lachnospira_pectinoschiza | 52 (44.1) | 0.00 | 0.967 | 0.993 |  | 0.00 | 0.992 | 0.992 |
| s__Megasphaera_sp_BL7 | 34 (28.8) | 0.00 | 0.980 | 0.994 |  | -0.01 | 0.926 | 0.987 |
| s__Blautia_SGB4815 | 65 (55.1) | 0.00 | 0.985 | 0.994 |  | 0.00 | 0.966 | 0.987 |
| s__Bifidobacterium_pseudocatenulatum | 90 (76.3) | 0.00 | 0.987 | 0.994 |  | 0.01 | 0.917 | 0.987 |
| s__Veillonella_rogosae | 31 (26.3) | 0.00 | 0.989 | 0.994 |  | -0.01 | 0.917 | 0.987 |
| s__Anaerostipes_hadrus | 107 (90.7) | 0.00 | 0.998 | 0.998 |  | 0.01 | 0.894 | 0.987 |

**Table S16. Differentially abundant species between baseline and 1 month p.v. shared by BNT162b2 and CoronaVac vaccinees.**

|  |  | BNT162b2 | | | |  | CoronaVac | | | |
| --- | --- | --- | --- | --- | --- | --- | --- | --- | --- | --- |
| Phylum | Species | Baseline abundance | 1m p.v. abundance | Direction of enrichment | FDR |  | Baseline abundance | 1m p.v. abundance | Direction of enrichment | FDR |
| Actinomycetota | *Bifidobacterium pseudocatenulatum* | 0.31 (0, 1.41) | 0.16 (0, 0.66) | -0.00295814 | 0.002 |  | 0.28 (0.08, 1.39) | 0.17 (0.02, 0.61) | -0.0043102 | 0.022 |
| Actinomycetota | *Collinsella aerofaciens* | 0.47 (0.13, 1.08) | 0.24 (0.07, 0.72) | -0.00191811 | 0.017 |  | 0.31 (0.02, 0.66) | 0.07 (0.01, 0.31) | -0.0016681 | 0.039 |
| Actinomycetota | *Adlercreutzia equolifaciens* | 0.1 (0, 0.37) | 0.05 (0, 0.2) | -0.00088538 | 0.009 |  | 0.25 (0.03, 0.5) | 0.03 (0, 0.16) | -0.0021521 | <0.001 |
| Bacteroidota | *Bacteroides cellulosilyticus* | 0.01 (0, 0.1) | 0.02 (0, 0.2) | 0.000380106 | 0.096 |  | 0.12 (0, 0.38) | 0.15 (0, 0.66) | 0.0022712 | 0.005 |
| Bacteroidota | *Parabacteroides merdae* | 0.11 (0, 0.31) | 0.17 (0, 0.45) | 0.000965613 | 0.065 |  | 0.01 (0, 0.07) | 0.01 (0, 0.18) | 0.0006308 | 0.059 |
| Bacteroidota | *Bacteroides caccae* | 0.06 (0, 0.32) | 0.13 (0, 0.57) | 0.001362146 | 0.003 |  | 0.11 (0, 0.28) | 0.22 (0, 0.48) | 0.0015477 | 0.028 |
| Bacteroidota | *Blautia wexlerae* | 1.83 (0.72, 3.18) | 0.79 (0.27, 1.55) | -0.01018727 | <0.001 | | 0.09 (0.03, 0.3) | 0.04 (0.01, 0.1) | -0.0010938 | 0.027 |
| Bacteroidota | *Anaerobutyricum hallii* | 0.65 (0.31, 1.15) | 0.3 (0.14, 0.6) | -0.00327848 | <0.001 | | 0 (0, 0.27) | 0 (0, 0.12) | -0.003782 | 0.039 |
| Bacteroidota | *Anaerostipes hadrus* | 0.47 (0.21, 1.22) | 0.3 (0.1, 0.79) | -0.00318613 | <0.001 | | 0.65 (0.24, 1.16) | 0.21 (0.07, 0.54) | -0.0035358 | 0.001 |
| Bacteroidota | *Fusicatenibacter saccharivorans* | 0.72 (0.35, 1.44) | 0.51 (0.17, 0.93) | -0.00288075 | <0.001 | | 0.13 (0, 0.41) | 0.06 (0, 0.11) | -0.0021803 | 0.013 |
| Bacteroidota | *Blautia massiliensis* | 0.3 (0.06, 0.78) | 0.1 (0.02, 0.3) | -0.00259085 | <0.001 | | 0.15 (0.05, 0.37) | 0.09 (0.03, 0.19) | -0.0011167 | 0.045 |
| Bacteroidota | *Dorea longicatena* | 0.25 (0.08, 0.68) | 0.17 (0.05, 0.31) | -0.00240329 | <0.001 | | 0.1 (0, 0.22) | 0.03 (0, 0.09) | -0.0009774 | 0.051 |
| Bacteroidota | *Clostridiaceae bacterium* | 0.32 (0.13, 0.86) | 0.17 (0.06, 0.39) | -0.00237834 | <0.001 | | 0.66 (0.44, 1.15) | 0.24 (0.14, 0.44) | -0.0039056 | 0.013 |
| Bacteroidota | *Ruminococcus torques* | 0.31 (0.06, 0.87) | 0.15 (0.05, 0.46) | -0.00219176 | <0.001 | | 1.03 (0.19, 1.79) | 0.23 (0.04, 0.65) | -0.0077165 | <0.001 |
| Bacteroidota | *Dorea formicigenerans* | 0.16 (0.03, 0.5) | 0.06 (0, 0.18) | -0.00203939 | <0.001 | | 0.2 (0.1, 0.39) | 0.09 (0.04, 0.18) | -0.0012347 | 0.005 |
| Bacteroidota | *Clostridia unclassified SGB4447* | 0 (0, 0.24) | 0 (0, 0.12) | -0.00190528 | 0.047 |  | 0.24 (0.04, 0.84) | 0.08 (0.02, 0.2) | -0.0034103 | 0.002 |
| Bacteroidota | *Coprococcus comes* | 0.1 (0, 0.31) | 0.07 (0, 0.18) | -0.00148324 | <0.001 | | 0.13 (0.07, 0.32) | 0.02 (0.01, 0.06) | -0.0012744 | <0.001 |
| Bacteroidota | *Evtepia gabavorous* | 0 (0, 0.22) | 0 (0, 0.07) | -0.00143755 | 0.004 |  | 2.59 (0.72, 4.26) | 0.59 (0.29, 1.34) | -0.0169023 | <0.001 |
| Bacteroidota | *Blautia obeum* | 0.14 (0.03, 0.36) | 0.04 (0.01, 0.11) | -0.00117783 | <0.001 | | 0.19 (0, 0.64) | 0.06 (0, 0.15) | -0.0027573 | 0.001 |
| Bacteroidota | *Streptococcus salivarius* | 0.11 (0.03, 0.29) | 0.02 (0, 0.1) | -0.0011594 | <0.001 | | 0.26 (0.12, 0.54) | 0.11 (0.03, 0.16) | -0.0020391 | <0.001 |
| Bacteroidota | *Blautia faecis* | 0.15 (0.08, 0.38) | 0.07 (0.04, 0.14) | -0.00113241 | <0.001 | | 0.32 (0.1, 0.75) | 0.13 (0.04, 0.33) | -0.0031893 | <0.001 |
| Bacteroidota | *Agathobaculum butyriciproducens* | 0.34 (0.13, 0.63) | 0.21 (0.1, 0.47) | -0.00096293 | 0.007 |  | 0.14 (0, 0.79) | 0.04 (0, 0.31) | -0.0036053 | 0.015 |
| Bacteroidota | *Eubacterium ramulus* | 0.11 (0, 0.37) | 0.06 (0, 0.19) | -0.00095712 | 0.024 |  | 0.94 (0.55, 1.57) | 0.45 (0.28, 0.62) | -0.0046719 | 0.011 |
| Bacteroidota | *Faecalicatena fissicatena* | 0.03 (0, 0.23) | 0.01 (0, 0.17) | -0.00076656 | 0.013 |  | 0 (0, 0.35) | 0 (0, 0.15) | -0.0014578 | 0.07 |
| Bacteroidota | *Ruminococcus gnavus* | 0.04 (0, 0.34) | 0.02 (0, 0.14) | -0.00067558 | 0.012 |  | 0.46 (0.12, 1.28) | 0.24 (0.05, 0.65) | -0.0023535 | 0.026 |
| Bacteroidota | *Eubacterium ventriosum* | 0.07 (0, 0.22) | 0.04 (0, 0.13) | -0.0005025 | 0.025 |  | 0.47 (0.23, 0.83) | 0.18 (0.06, 0.42) | -0.002801 | 0.017 |
| Pseudomonadota | *Sutterella wadsworthensis* | 0 (0, 0.58) | 0 (0, 0.81) | 3.27E-03 | 0.025 |  | 0.06 (0, 0.74) | 0.15 (0, 1.25) | 4.06E-03 | 0.027 |

Direction of enrichment: <0, enriched at baseline; >0, enriched at 1 month post vaccination (p.v.).

**Table S17. Correlations between age and alpha diversity indices, and baseline relative abundances and fold changes in relative abundance of the instable species.**

|  | **BNT162b2** | | |  | **CoronaVac** | | |
| --- | --- | --- | --- | --- | --- | --- | --- |
|  | Feature | Spearman's Rho | P value |  | Feature | Spearman's Rho | P value |
| Alpha diversity indices | Bacterial Shannon diversity: Baseline - M1 | 0.08 | 0.368 |  | Bacterial Shannon diversity: Baseline - M1 | 0.10 | 0.534 |
|  | Bacterial Shannon diversity: Baseline - M6 | 0.20 | **0.044** |  | Bacterial Shannon diversity: Baseline - M6 | -0.19 | 0.353 |
|  | Viral Shannon diversity: Baseline - M1 | 0.01 | 0.872 |  | Viral Shannon diversity: Baseline - M1 | -0.06 | 0.701 |
|  | Viral Shannon diversity: Baseline - M6 | 0.13 | 0.208 |  | Viral Shannon diversity: Baseline - M6 | 0.07 | 0.746 |
|  | Viral abundance: Baseline - M1 | -0.17 | 0.070 |  | Viral abundance: Baseline - M1 | -0.12 | 0.460 |
|  | Viral abundance: Baseline - M6 | -0.13 | 0.213 |  | Viral abundance: Baseline - M6 | 0.11 | 0.597 |
| Baseline relative abundance of the instable species | Blautia wexlerae | 0.16 | 0.117 |  | Blautia wexlerae | -0.08 | 0.713 |
|  | Anaerobutyricum hallii | 0.06 | 0.555 |  | Anaerobutyricum hallii | 0.22 | 0.278 |
|  | Anaerostipes hadrus | 0.06 | 0.538 |  | Anaerostipes hadrus | 0.04 | 0.844 |
|  | Bifidobacterium pseudocatenulatum | 0.10 | 0.322 |  | Bifidobacterium pseudocatenulatum | -0.47 | 0.015 |
|  | Fusicatenibacter saccharivorans | 0.05 | 0.621 |  | Fusicatenibacter saccharivorans | -0.04 | 0.832 |
|  | Blautia massiliensis | 0.00 | 0.985 |  | Blautia massiliensis | -0.16 | 0.430 |
|  | Ruminococcus torques | 0.01 | 0.889 |  | Ruminococcus torques | -0.15 | 0.460 |
|  | Collinsella aerofaciens | 0.19 | 0.059 |  | Collinsella aerofaciens | -0.16 | 0.446 |
|  | Clostridia unclassified SGB4447 | 0.08 | 0.460 |  | Clostridia unclassified SGB4447 | 0.06 | 0.786 |
|  | Evtepia gabavorous | -0.02 | 0.883 |  | Evtepia gabavorous | -0.32 | 0.116 |
|  | Blautia obeum | 0.06 | 0.532 |  | Blautia obeum | -0.03 | 0.886 |
|  | Streptococcus salivarius | -0.06 | 0.589 |  | Streptococcus salivarius | 0.05 | 0.806 |
|  | Blautia faecis | -0.03 | 0.774 |  | Blautia faecis | 0.19 | 0.352 |
|  | Agathobaculum butyriciproducens | 0.01 | 0.946 |  | Agathobaculum butyriciproducens | -0.15 | 0.458 |
|  | Eubacterium ramulus | 0.08 | 0.455 |  | Eubacterium ramulus | 0.06 | 0.753 |
|  | Bacteroides cellulosilyticus | -0.03 | 0.738 |  | Bacteroides cellulosilyticus | 0.11 | 0.591 |
| Fold change (M6/Baseline) of the instable species | Blautia wexlerae | -0.18 | 0.073 |  | Blautia wexlerae | 0.08 | 0.710 |
|  | Anaerobutyricum hallii | -0.20 | 0.050 |  | Anaerobutyricum hallii | -0.12 | 0.575 |
|  | Anaerostipes hadrus | -0.03 | 0.754 |  | Anaerostipes hadrus | -0.20 | 0.316 |
|  | Bifidobacterium pseudocatenulatum | -0.10 | 0.333 |  | Bifidobacterium pseudocatenulatum | 0.23 | 0.253 |
|  | Fusicatenibacter saccharivorans | -0.20 | **0.047** |  | Fusicatenibacter saccharivorans | 0.06 | 0.771 |
|  | Blautia massiliensis | -0.03 | 0.787 |  | Blautia massiliensis | 0.10 | 0.626 |
|  | Ruminococcus torques | -0.10 | 0.313 |  | Ruminococcus torques | 0.25 | 0.223 |
|  | Collinsella aerofaciens | -0.04 | 0.690 |  | Collinsella aerofaciens | 0.20 | 0.321 |
|  | Clostridia unclassified SGB4447 | -0.21 | **0.037** |  | Clostridia unclassified SGB4447 | -0.14 | 0.484 |
|  | Evtepia gabavorous | 0.11 | 0.277 |  | Evtepia gabavorous | 0.07 | 0.719 |
|  | Blautia obeum | -0.08 | 0.407 |  | Blautia obeum | -0.02 | 0.921 |
|  | Streptococcus salivarius | -0.11 | 0.269 |  | Streptococcus salivarius | -0.08 | 0.705 |
|  | Blautia faecis | -0.10 | 0.344 |  | Blautia faecis | 0.02 | 0.917 |
|  | Agathobaculum butyriciproducens | -0.11 | 0.261 |  | Agathobaculum butyriciproducens | -0.25 | 0.210 |
|  | Eubacterium ramulus | -0.09 | 0.355 |  | Eubacterium ramulus | -0.15 | 0.460 |
|  | Bacteroides cellulosilyticus | 0.01 | 0.896 |  | Bacteroides cellulosilyticus | -0.09 | 0.650 |

**Table S18. Differentially abundant microbial funcitonal pathways between baseline and 1 month p.v. in the BNT162b2 group.**

|  | Relative abundance (%) | | |  | Baseline vs. 1 month | |  | Baseline vs. 6 months | |  | Sig. diff. between baseline and 1 month in CoronaVac |
| --- | --- | --- | --- | --- | --- | --- | --- | --- | --- | --- | --- |
| Pathway | Baseline | 1 month p.v. | 6 months p.v. |  | Direction of enrichment | FDR |  | Direction of enrichment | FDR |  |  |
| HSERMETANA-PWY: L-methionine biosynthesis III | 0.56 (0.5, 0.65) | 0.5 (0.44, 0.59) | 0.52 (0.44, 0.6) |  | -0.00056399 | <0.001 |  | -0.00043533 | **0.023** |  | Y |
| PWY-6147: 6-hydroxymethyl-dihydropterin diphosphate biosynthesis I | 0.32 (0.23, 0.48) | 0.37 (0.28, 0.52) | 0.37 (0.28, 0.46) |  | 0.00043789 | 0.059 |  | 0.00050213 | **0.094** |  | Y |
| PWY-6936: seleno-amino acid biosynthesis (plants) | 0.48 (0.37, 0.6) | 0.41 (0.32, 0.48) | 0.37 (0.28, 0.45) |  | -0.00084474 | <0.001 |  | -0.00113218 | **<0.001** | | Y |
| HISDEG-PWY: L-histidine degradation I | 0.21 (0.13, 0.32) | 0.28 (0.19, 0.41) | 0.32 (0.21, 0.43) |  | 0.00058039 | <0.001 |  | 0.00081754 | **<0.001** | | Y |
| PWY-7111: pyruvate fermentation to isobutanol (engineered) | 0.76 (0.64, 0.86) | 0.68 (0.57, 0.8) | 0.68 (0.58, 0.77) |  | -0.00085322 | <0.001 |  | -0.00091815 | **<0.001** | | Y |
| ARGININE-SYN4-PWY: L-ornithine biosynthesis II | 0.24 (0.12, 0.38) | 0.31 (0.19, 0.44) | 0.35 (0.21, 0.46) |  | 0.00042972 | 0.034 |  | 0.00058096 | **0.021** |  | Y |
| PWY-702: L-methionine biosynthesis II | 0.18 (0.11, 0.25) | 0.17 (0.09, 0.22) | 0.13 (0.1, 0.19) |  | -0.00025256 | 0.046 |  | -0.0004739 | **0.003** |  | Y |
| PWY-7199: pyrimidine deoxyribonucleosides salvage | 0.66 (0.56, 0.75) | 0.72 (0.6, 0.8) | 0.72 (0.63, 0.83) |  | 0.00049885 | 0.02 |  | 0.00059549 | **0.012** |  | Y |
| PWY-8178: pentose phosphate pathway (non-oxidative branch) II | 0.74 (0.64, 0.87) | 0.68 (0.6, 0.75) | 0.69 (0.62, 0.79) |  | -0.00072945 | <0.001 |  | -0.00059015 | **0.02** |  | Y |
| CITRULBIO-PWY: L-citrulline biosynthesis | 0.21 (0.12, 0.35) | 0.27 (0.18, 0.36) | 0.28 (0.17, 0.38) |  | 0.00038832 | 0.02 |  | 0.00049154 | **0.018** |  | Y |
| PWY-5030: L-histidine degradation III | 0.16 (0.1, 0.29) | 0.21 (0.14, 0.33) | 0.24 (0.16, 0.33) |  | 0.00039474 | 0.013 |  | 0.00054484 | **0.008** |  | Y |
| NONOXIPENT-PWY: pentose phosphate pathway (non-oxidative branch) I | 0.65 (0.53, 0.76) | 0.57 (0.47, 0.67) | 0.58 (0.49, 0.7) |  | -0.00062604 | 0.001 |  | -0.00058959 | **0.014** |  | Y |
| PWY-5695: inosine 5 -phosphate degradation | 0.84 (0.76, 0.94) | 0.89 (0.8, 0.97) | 0.91 (0.82, 0.99) |  | 0.00032803 | 0.073 |  | 0.00072133 | **0.001** |  | Y |
| PWY-1269: CMP-3-deoxy-D-manno-octulosonate biosynthesis | 0.2 (0.11, 0.26) | 0.22 (0.16, 0.31) | 0.21 (0.16, 0.29) |  | 0.0003778 | 0.019 |  | 0.00042268 | **0.008** |  | Y |
| PWY-7237: myo-, chiro- and scyllo-inositol degradation | 0.57 (0.44, 0.65) | 0.49 (0.36, 0.62) | 0.48 (0.34, 0.61) |  | -0.00053777 | 0.028 |  | -0.00076295 | **0.012** |  | Y |
| ARGSYN-PWY: L-arginine biosynthesis I (via L-ornithine) | 0.88 (0.75, 1.04) | 0.83 (0.66, 0.99) | 0.87 (0.69, 0.97) |  | -0.00066716 | 0.009 |  | -0.00052324 | **0.05** |  | Y |
| PWY-6305: superpathway of putrescine biosynthesis | 0.11 (0.07, 0.17) | 0.14 (0.09, 0.19) | 0.14 (0.09, 0.18) |  | 0.00023134 | 0.017 |  | 0.00026559 | **0.011** |  | Y |
| PYRIDOXSYN-PWY: pyridoxal 5 -phosphate biosynthesis I | 0.15 (0.09, 0.26) | 0.2 (0.14, 0.31) | 0.24 (0.14, 0.33) |  | 0.00028688 | 0.041 |  | 0.0005211 | **0.012** |  | Y |
| PWY-4984: urea cycle | 0.14 (0.09, 0.26) | 0.18 (0.11, 0.27) | 0.2 (0.12, 0.3) |  | 0.00020163 | 0.092 |  | 0.00040858 | **0.017** |  | Y |
| NAGLIPASYN-PWY: lipid IVA biosynthesis (E. coli) | 0.11 (0.07, 0.21) | 0.17 (0.1, 0.24) | 0.18 (0.12, 0.24) |  | 0.00037055 | 0.001 |  | 0.00045204 | **0.001** |  | Y |
| PWY-8073: lipid IVA biosynthesis (P. putida) | 0.11 (0.07, 0.21) | 0.17 (0.1, 0.24) | 0.18 (0.12, 0.24) |  | 0.00037055 | 0.001 |  | 0.00045204 | **0.001** |  | Y |
| PWY0-845: superpathway of pyridoxal 5 -phosphate biosynthesis and salvage | 0.19 (0.12, 0.31) | 0.24 (0.17, 0.34) | 0.28 (0.16, 0.37) |  | 0.00028542 | 0.043 |  | 0.00052762 | **0.012** |  | Y |
| PWY-7560: methylerythritol phosphate pathway II | 0.64 (0.56, 0.75) | 0.58 (0.53, 0.67) | 0.6 (0.51, 0.7) |  | -0.00060087 | 0.001 |  | -0.00042307 | **0.078** |  | Y |
| BIOTIN-BIOSYNTHESIS-PWY: biotin biosynthesis I | 0.17 (0.11, 0.24) | 0.19 (0.14, 0.28) | 0.19 (0.12, 0.27) |  | 0.00037012 | 0.002 |  | 0.00022233 | **0.055** |  | Y |
| PWY-6519: 8-amino-7-oxononanoate biosynthesis I | 0.17 (0.1, 0.23) | 0.19 (0.12, 0.28) | 0.18 (0.11, 0.26) |  | 0.00041848 | 0.001 |  | 0.00024415 | **0.073** |  | Y |
| PWY0-1477: ethanolamine utilization | 0.12 (0.07, 0.19) | 0.17 (0.1, 0.3) | 0.13 (0.09, 0.23) |  | 0.00058779 | <0.001 |  | 0.00032127 | **0.074** |  | Y |
| GLYCOGENSYNTH-PWY: glycogen biosynthesis I (from ADP-D-Glucose) | 1.04 (0.87, 1.25) | 0.93 (0.7, 1.11) | 0.9 (0.8, 1.11) |  | -0.00127725 | <0.001 |  | -0.00127839 | **<0.001** | | Y |
| P41-PWY: pyruvate fermentation to acetate and (S)-lactate I | 0.47 (0.38, 0.57) | 0.39 (0.3, 0.5) | 0.41 (0.34, 0.49) |  | -0.00081479 | <0.001 |  | -0.00081805 | **<0.001** | | Y |
| PWY-1861: formaldehyde assimilation II (assimilatory RuMP Cycle) | 0.1 (0.06, 0.19) | 0.05 (0.02, 0.1) | 0.07 (0.04, 0.15) |  | -0.00050921 | <0.001 |  | -0.00031612 | **0.004** |  | Y |
| PWY-5100: pyruvate fermentation to acetate and lactate II | 0.48 (0.4, 0.58) | 0.4 (0.32, 0.51) | 0.41 (0.36, 0.49) |  | -0.00078345 | <0.001 |  | -0.00074244 | **<0.001** | | Y |
| PWY-5941: glycogen degradation II | 1.04 (0.84, 1.23) | 0.89 (0.73, 1.07) | 0.91 (0.79, 1.05) |  | -0.00128569 | <0.001 |  | -0.00135468 | **<0.001** | | Y |
| PWY-6270: isoprene biosynthesis I | 0.64 (0.55, 0.73) | 0.58 (0.49, 0.65) | 0.57 (0.49, 0.69) |  | -0.00070651 | <0.001 |  | -0.00061213 | **0.002** |  | Y |
| PWY-6317: D-galactose degradation I (Leloir pathway) | 0.65 (0.53, 0.74) | 0.55 (0.45, 0.67) | 0.58 (0.49, 0.65) |  | -0.00087214 | <0.001 |  | -0.0007851 | **<0.001** | | Y |
| PWY-6527: stachyose degradation | 0.53 (0.42, 0.61) | 0.43 (0.33, 0.51) | 0.44 (0.35, 0.54) |  | -0.0009247 | <0.001 |  | -0.00072792 | **0.001** |  | Y |
| PWY-6823: molybdopterin biosynthesis | 0.41 (0.31, 0.52) | 0.35 (0.26, 0.45) | 0.34 (0.26, 0.43) |  | -0.00049389 | 0.014 |  | -0.00064884 | **0.003** |  | Y |
| P124-PWY: Bifidobacterium shunt | 0.11 (0.04, 0.2) | 0.07 (0.03, 0.15) | 0.07 (0.03, 0.14) |  | -0.0002158 | 0.056 |  | -0.00028026 | **0.006** |  | N/A |
| PWY-6549: L-glutamine biosynthesis III | 0.11 (0.08, 0.16) | 0.1 (0.07, 0.14) | 0.09 (0.07, 0.13) |  | -0.00012923 | 0.07 |  | -0.00019569 | **0.019** |  | N/A |
| PWY0-1261: anhydromuropeptides recycling I | 0.13 (0.09, 0.19) | 0.16 (0.11, 0.22) | 0.17 (0.11, 0.21) |  | 0.00031324 | 0.004 |  | 0.00026109 | **0.02** |  | N |
| PWY-7282: 4-amino-2-methyl-5-diphosphomethylpyrimidine biosynthesis II | 0.3 (0.23, 0.39) | 0.33 (0.27, 0.4) | 0.34 (0.27, 0.43) |  | 0.0002381 | 0.082 |  | 0.00051131 | **0.006** |  | N |
| PWY66-409: superpathway of purine nucleotide salvage | 0.17 (0.12, 0.24) | 0.14 (0.08, 0.21) | 0.13 (0.1, 0.21) |  | -0.0002855 | 0.021 |  | -0.00036398 | **0.012** |  | N |
| GLUCOSE1PMETAB-PWY: glucose and glucose-1-phosphate degradation | 0.49 (0.37, 0.6) | 0.37 (0.29, 0.52) | 0.36 (0.26, 0.47) |  | -0.00071669 | 0.005 |  | -0.00132798 | **<0.001** | | N |
| PWY-6906: chitin derivatives degradation | 0.07 (0.02, 0.12) | 0.09 (0.04, 0.15) | 0.08 (0.04, 0.15) |  | 0.00022102 | 0.008 |  | 0.00025396 | **0.021** |  | N |
| RIBOSYN2-PWY: flavin biosynthesis I (bacteria and plants) | 0.62 (0.56, 0.71) | 0.59 (0.49, 0.7) | 0.6 (0.51, 0.66) |  | -0.00035851 | 0.062 |  | -0.00034076 | **0.033** |  | N |
| PWY-7383: anaerobic energy metabolism (invertebrates, cytosol) | 0.12 (0.07, 0.18) | 0.13 (0.07, 0.22) | 0.15 (0.07, 0.22) |  | 0.00021813 | 0.062 |  | 0.00020059 | **0.049** |  | N |
| PWY-6151: S-adenosyl-L-methionine salvage I | 0.94 (0.84, 1.04) | 0.89 (0.77, 0.98) | 0.92 (0.8, 1.02) |  | -0.00052273 | 0.021 |  | -0.00035275 | **0.097** |  | N |
| CALVIN-PWY: Calvin-Benson-Bassham cycle | 0.86 (0.78, 0.94) | 0.79 (0.72, 0.86) | 0.82 (0.76, 0.92) |  | -0.00055902 | <0.001 |  | -0.00028327 | 0.137 |  | Y |
| PWY0-1296: purine ribonucleosides degradation | 0.75 (0.63, 0.88) | 0.71 (0.54, 0.87) | 0.74 (0.62, 0.87) |  | -0.00055665 | 0.021 |  | -0.00029061 | 0.204 |  | Y |
| GLUTORN-PWY: L-ornithine biosynthesis I | 0.79 (0.67, 0.93) | 0.73 (0.57, 0.89) | 0.75 (0.64, 0.9) |  | -0.00058694 | 0.021 |  | -0.00031917 | 0.207 |  | Y |
| PWY-6470: peptidoglycan biosynthesis V (&beta;-lactam resistance) | 0.15 (0.1, 0.22) | 0.11 (0.07, 0.18) | 0.14 (0.08, 0.21) |  | -0.00036412 | <0.001 |  | -0.00014795 | 0.18 |  | Y |
| PWY-7238: sucrose biosynthesis II | 1.3 (1.06, 1.58) | 1.25 (0.96, 1.49) | 1.28 (1.05, 1.48) |  | -0.00085299 | 0.027 |  | -0.000655 | 0.106 |  | Y |
| ANAGLYCOLYSIS-PWY: glycolysis III (from glucose) | 0.94 (0.86, 0.99) | 0.88 (0.78, 0.97) | 0.95 (0.86, 1.01) |  | -0.0004986 | 0.001 |  | -8.97E-05 | 0.675 |  | Y |
| ARGSYNBSUB-PWY: L-arginine biosynthesis II (acetyl cycle) | 0.89 (0.75, 1.06) | 0.85 (0.68, 1.02) | 0.87 (0.69, 1.05) |  | -0.00055498 | 0.063 |  | -0.00037166 | 0.218 |  | Y |
| COMPLETE-ARO-PWY: superpathway of aromatic amino acid biosynthesis | 0.96 (0.87, 1.02) | 0.93 (0.83, 1.01) | 0.96 (0.87, 1.01) |  | -0.0004274 | 0.022 |  | -0.00015238 | 0.241 |  | Y |
| PWY-7357: thiamine phosphate formation from pyrithiamine and oxythiamine (yeast) | 0.7 (0.6, 0.78) | 0.64 (0.55, 0.73) | 0.65 (0.56, 0.74) |  | -0.00042903 | 0.02 |  | -0.00032927 | 0.106 |  | Y |
| PWY-6168: flavin biosynthesis III (fungi) | 0.11 (0.05, 0.21) | 0.15 (0.08, 0.25) | 0.12 (0.06, 0.23) |  | 0.00034058 | 0.056 |  | 0.00013496 | 0.457 |  | Y |
| **DTDPRHAMSYN-PWY: dTDP-&beta;-L-rhamnose biosynthesis** | 1.18 (1.02, 1.32) | 1.14 (0.9, 1.28) | 1.14 (1.01, 1.31) |  | -0.00055866 | 0.085 |  | -0.00010879 | 0.838 |  | Y |
| FASYN-ELONG-PWY: fatty acid elongation -- saturated | 0.19 (0.12, 0.27) | 0.23 (0.14, 0.33) | 0.2 (0.13, 0.29) |  | 0.00042062 | 0.002 |  | 0.00025523 | 0.106 |  | Y |
| PWY-7664: oleate biosynthesis IV (anaerobic) | 0.17 (0.11, 0.25) | 0.21 (0.12, 0.32) | 0.18 (0.11, 0.27) |  | 0.00041847 | 0.003 |  | 0.00023078 | 0.13 |  | Y |
| SER-GLYSYN-PWY: superpathway of L-serine and glycine biosynthesis I | 0.6 (0.52, 0.68) | 0.56 (0.49, 0.64) | 0.6 (0.5, 0.66) |  | -0.00036224 | 0.003 |  | -0.00025957 | 0.113 |  | Y |
| PWY0-862: (5Z)-dodecenoate biosynthesis I | 0.16 (0.1, 0.23) | 0.19 (0.11, 0.3) | 0.16 (0.1, 0.25) |  | 0.00040301 | 0.003 |  | 0.00021131 | 0.153 |  | Y |
| P164-PWY: purine nucleobases degradation I (anaerobic) | 0.11 (0.07, 0.15) | 0.09 (0.07, 0.12) | 0.1 (0.07, 0.15) |  | -0.00020453 | 0.002 |  | -0.00011763 | 0.153 |  | Y |
| PWY-5188: tetrapyrrole biosynthesis I (from glutamate) | 0.29 (0.24, 0.35) | 0.26 (0.21, 0.32) | 0.26 (0.21, 0.33) |  | -0.0003018 | 0.019 |  | -0.0001877 | 0.202 |  | Y |
| PWY-6282: palmitoleate biosynthesis I (from (5Z)-dodec-5-enoate) | 0.15 (0.09, 0.23) | 0.19 (0.11, 0.3) | 0.16 (0.1, 0.24) |  | 0.00041448 | 0.003 |  | 0.00021253 | 0.133 |  | N |
| PWY-5103: L-isoleucine biosynthesis III | 0.84 (0.76, 0.95) | 0.79 (0.69, 0.92) | 0.82 (0.73, 0.92) |  | -0.00056369 | 0.015 |  | -0.00011123 | 0.724 |  | N |
| PWY-6731: starch degradation III | 0.28 (0.21, 0.35) | 0.26 (0.19, 0.32) | 0.31 (0.24, 0.38) |  | -0.00023241 | 0.043 |  | 0.00013725 | 0.36 |  | N |
| ILEUSYN-PWY: L-isoleucine biosynthesis I (from threonine) | 0.95 (0.87, 1.04) | 0.91 (0.81, 1.03) | 0.94 (0.86, 1.03) |  | -0.00044688 | 0.028 |  | -6.00E-05 | 0.847 |  | N |
| PWY-6606: guanosine nucleotides degradation II | 0.1 (0.07, 0.14) | 0.09 (0.06, 0.12) | 0.09 (0.07, 0.12) |  | -0.00014043 | 0.074 |  | -7.70E-05 | 0.153 |  | N |
| PWY-5989: stearate biosynthesis II (bacteria and plants) | 0.15 (0.1, 0.22) | 0.18 (0.11, 0.3) | 0.16 (0.1, 0.24) |  | 0.00036999 | 0.004 |  | 0.0002206 | 0.153 |  | N |
| BRANCHED-CHAIN-AA-SYN-PWY: superpathway of branched chain amino acid biosynthesis | 0.89 (0.82, 0.99) | 0.86 (0.76, 0.97) | 0.89 (0.8, 0.99) |  | -0.0004889 | 0.022 |  | -4.23E-05 | 0.945 |  | N |
| PWY0-162: superpathway of pyrimidine ribonucleotides de novo biosynthesis | 0.3 (0.27, 0.38) | 0.33 (0.29, 0.42) | 0.35 (0.27, 0.43) |  | 0.00018536 | 0.043 |  | 0.00017212 | 0.132 |  | N |
| PWY-6353: purine nucleotides degradation II (aerobic) | 0.18 (0.13, 0.25) | 0.16 (0.11, 0.23) | 0.16 (0.13, 0.22) |  | -0.00018411 | 0.082 |  | -0.00011798 | 0.201 |  | N |
| PWY-1042: glycolysis IV | 1.18 (1.08, 1.3) | 1.13 (0.99, 1.21) | 1.17 (1.06, 1.29) |  | -0.00071239 | 0.003 |  | -0.00016549 | 0.626 |  | N |
| PWY-2942: L-lysine biosynthesis III | 0.83 (0.78, 0.89) | 0.82 (0.72, 0.88) | 0.85 (0.78, 0.93) |  | -0.00023163 | 0.084 |  | 0.00016975 | 0.331 |  | N |
| PWY-6969: TCA cycle V (2-oxoglutarate synthase) | 0.11 (0.08, 0.14) | 0.13 (0.09, 0.18) | 0.11 (0.08, 0.15) |  | 0.00024488 | <0.001 |  | 0.00012317 | 0.114 |  | N |
| 1CMET2-PWY: folate transformations III (E. coli) | 0.78 (0.7, 0.86) | 0.77 (0.67, 0.84) | 0.8 (0.74, 0.88) |  | -0.0002451 | 0.082 |  | 0.00018236 | 0.39 |  | N |

**Table S19. Differentially abundant microbial funcitonal pathways between baseline and 1 month p.v. in the CoronaVac group.**

|  | Relative abundance (%) | | |  | Baseline vs. 1 month | |  | Baseline vs. 6 months | |  | Sig. diff. between baseline and 1 month in BNT162b2 |
| --- | --- | --- | --- | --- | --- | --- | --- | --- | --- | --- | --- |
| Pathway | Baseline | 1 month p.v. | 6 months p.v. |  | Direction of enrichment | FDR |  | Direction of enrichment | FDR |  |  |
| PWY-6147: 6-hydroxymethyl-dihydropterin diphosphate biosynthesis I | 0.31 (0.24, 0.41) | 0.47 (0.36, 0.59) | 0.44 (0.3, 0.53) |  | 0.001372335 | 0.001 |  | 0.001321183 | **0.016** |  | Y |
| HSERMETANA-PWY: L-methionine biosynthesis III | 0.54 (0.49, 0.69) | 0.5 (0.35, 0.55) | 0.5 (0.38, 0.61) |  | -1.03E-03 | 0.001 |  | -8.26E-04 | **0.016** |  | Y |
| PWY-6936: seleno-amino acid biosynthesis (plants) | 0.44 (0.33, 0.55) | 0.31 (0.23, 0.45) | 0.35 (0.27, 0.43) |  | -0.001040594 | 0.001 |  | -0.00115271 | **0.015** |  | Y |
| HISDEG-PWY: L-histidine degradation I | 0.21 (0.15, 0.31) | 0.33 (0.25, 0.43) | 0.38 (0.28, 0.5) |  | 9.84E-04 | 0.002 |  | 0.001522516 | **0.002** |  | Y |
| PWY-7111: pyruvate fermentation to isobutanol (engineered) | 0.72 (0.63, 0.82) | 0.59 (0.45, 0.7) | 0.62 (0.53, 0.73) |  | -0.001240879 | 0.002 |  | -0.001036757 | **0.028** |  | Y |
| PWY-7199: pyrimidine deoxyribonucleosides salvage | 0.66 (0.58, 0.74) | 0.78 (0.67, 0.84) | 0.75 (0.69, 0.79) |  | 0.000916228 | 0.004 |  | 0.001106763 | **0.017** |  | Y |
| ARGININE-SYN4-PWY: L-ornithine biosynthesis II | 0.29 (0.18, 0.4) | 0.35 (0.26, 0.56) | 0.37 (0.31, 0.52) |  | 0.000936847 | 0.004 |  | 0.001322868 | **0.017** |  | Y |
| PWY-702: L-methionine biosynthesis II | 0.16 (0.13, 0.22) | 0.12 (0.07, 0.17) | 0.11 (0.09, 0.13) |  | -0.00048766 | 0.004 |  | -7.46E-04 | **0.015** |  | Y |
| PWY-8178: pentose phosphate pathway (non-oxidative branch) II | 0.77 (0.69, 0.88) | 0.66 (0.57, 0.76) | 0.68 (0.57, 0.75) |  | -0.00113485 | 0.004 |  | -9.16E-04 | **0.063** |  | Y |
| PWY-5030: L-histidine degradation III | 0.2 (0.11, 0.28) | 0.27 (0.19, 0.38) | 0.32 (0.22, 0.42) |  | 7.45E-04 | 0.005 |  | 1.38E-03 | **0.002** |  | Y |
| CITRULBIO-PWY: L-citrulline biosynthesis | 0.21 (0.14, 0.29) | 0.27 (0.2, 0.35) | 0.32 (0.17, 0.38) |  | 0.000745692 | 0.005 |  | 0.000713862 | **0.061** |  | Y |
| NONOXIPENT-PWY: pentose phosphate pathway (non-oxidative branch) I | 0.67 (0.56, 0.76) | 0.55 (0.43, 0.65) | 0.51 (0.44, 0.59) |  | -0.001059927 | 0.007 |  | -0.001337832 | **0.039** |  | Y |
| PWY-5695: inosine 5 -phosphate degradation | 0.85 (0.78, 0.93) | 0.94 (0.87, 1.01) | 0.96 (0.84, 1.02) |  | 0.00085812 | 0.008 |  | 1.10E-03 | **0.017** |  | Y |
| GLUTORN-PWY: L-ornithine biosynthesis I | 0.84 (0.73, 0.96) | 0.71 (0.63, 0.84) | 0.78 (0.6, 0.9) |  | -1.09E-03 | 0.009 |  | -1.18E-03 | **0.047** |  | Y |
| PWY-7238: sucrose biosynthesis II | 1.41 (1.25, 1.55) | 1.21 (0.98, 1.45) | 1.3 (1.14, 1.43) |  | -0.001706142 | 0.009 |  | -0.001444971 | **0.03** |  | Y |
| PWY-1269: CMP-3-deoxy-D-manno-octulosonate biosynthesis | 0.18 (0.13, 0.25) | 0.22 (0.18, 0.36) | 0.21 (0.15, 0.32) |  | 6.13E-04 | 0.011 |  | 6.95E-04 | **0.017** |  | Y |
| ARGSYN-PWY: L-arginine biosynthesis I (via L-ornithine) | 0.92 (0.78, 1) | 0.77 (0.66, 0.94) | 0.85 (0.71, 0.99) |  | -0.000962674 | 0.015 |  | -0.001081173 | **0.035** |  | Y |
| ARGSYNBSUB-PWY: L-arginine biosynthesis II (acetyl cycle) | 0.95 (0.8, 1.04) | 0.8 (0.64, 0.96) | 0.88 (0.71, 1.01) |  | -0.001160075 | 0.016 |  | -0.001212561 | **0.059** |  | Y |
| COMPLETE-ARO-PWY: superpathway of aromatic amino acid biosynthesis | 1.01 (0.95, 1.05) | 0.96 (0.88, 1) | 0.94 (0.89, 0.99) |  | -0.000638558 | 0.019 |  | -0.000615091 | **0.03** |  | Y |
| PYRIDOXSYN-PWY: pyridoxal 5 -phosphate biosynthesis I | 0.2 (0.12, 0.28) | 0.25 (0.16, 0.36) | 0.26 (0.16, 0.34) |  | 0.000574978 | 0.021 |  | 0.000853196 | **0.03** |  | Y |
| PWY-4984: urea cycle | 0.15 (0.09, 0.22) | 0.22 (0.13, 0.28) | 0.23 (0.14, 0.29) |  | 0.000553267 | 0.022 |  | 0.000608095 | **0.08** |  | Y |
| NAGLIPASYN-PWY: lipid IVA biosynthesis (E. coli) | 0.13 (0.08, 0.2) | 0.17 (0.1, 0.24) | 0.18 (0.12, 0.21) |  | 0.000416115 | 0.03 |  | 0.000470823 | **0.065** |  | Y |
| PWY-8073: lipid IVA biosynthesis (P. putida) | 0.13 (0.08, 0.2) | 0.17 (0.1, 0.24) | 0.18 (0.12, 0.21) |  | 0.000416115 | 0.03 |  | 0.000470823 | **0.065** |  | Y |
| PWY0-845: superpathway of pyridoxal 5 -phosphate biosynthesis and salvage | 0.24 (0.15, 0.33) | 0.3 (0.19, 0.39) | 0.3 (0.19, 0.38) |  | 5.31E-04 | 0.041 |  | 0.000808044 | **0.042** |  | Y |
| PWY-6519: 8-amino-7-oxononanoate biosynthesis I | 0.14 (0.09, 0.2) | 0.19 (0.12, 0.25) | 0.22 (0.15, 0.31) |  | 0.000359031 | 0.056 |  | 0.00066688 | **0.018** |  | Y |
| BIOTIN-BIOSYNTHESIS-PWY: biotin biosynthesis I | 0.15 (0.11, 0.22) | 0.21 (0.13, 0.26) | 0.24 (0.17, 0.33) |  | 0.000358918 | 0.056 |  | 0.000729853 | **0.015** |  | Y |
| FASYN-ELONG-PWY: fatty acid elongation -- saturated | 0.16 (0.1, 0.23) | 0.21 (0.12, 0.3) | 0.25 (0.17, 0.37) |  | 0.00037358 | 0.06 |  | 0.000788956 | **0.017** |  | Y |
| PWY-7664: oleate biosynthesis IV (anaerobic) | 0.15 (0.09, 0.22) | 0.2 (0.11, 0.29) | 0.23 (0.16, 0.35) |  | 3.48E-04 | 0.076 |  | 0.000720277 | **0.025** |  | Y |
| PWY0-862: (5Z)-dodecenoate biosynthesis I | 0.14 (0.08, 0.21) | 0.18 (0.1, 0.27) | 0.21 (0.14, 0.34) |  | 0.000311341 | 0.098 |  | 6.48E-04 | **0.03** |  | Y |
| P164-PWY: purine nucleobases degradation I (anaerobic) | 0.14 (0.1, 0.17) | 0.09 (0.06, 0.12) | 0.09 (0.05, 0.12) |  | -0.000440296 | <0.001 |  | -0.000561409 | **0.003** |  | Y |
| PWY-6823: molybdopterin biosynthesis | 0.45 (0.33, 0.57) | 0.33 (0.23, 0.41) | 0.38 (0.28, 0.44) |  | -1.16E-03 | <0.001 |  | -9.60E-04 | **0.077** |  | Y |
| PWY-5188: tetrapyrrole biosynthesis I (from glutamate) | 0.33 (0.26, 0.39) | 0.22 (0.18, 0.28) | 0.24 (0.17, 0.3) |  | -9.04E-04 | <0.001 |  | -1.03E-03 | **0.003** |  | Y |
| GLYCOGENSYNTH-PWY: glycogen biosynthesis I (from ADP-D-Glucose) | 1.13 (0.91, 1.25) | 0.92 (0.75, 1.08) | 0.95 (0.78, 1.09) |  | -0.001774112 | <0.001 |  | -0.001867867 | **0.002** |  | Y |
| P41-PWY: pyruvate fermentation to acetate and (S)-lactate I | 0.5 (0.41, 0.6) | 0.36 (0.27, 0.49) | 0.37 (0.31, 0.42) |  | -0.001165387 | <0.001 |  | -0.001115407 | **0.003** |  | Y |
| PWY-5100: pyruvate fermentation to acetate and lactate II | 0.5 (0.41, 0.6) | 0.38 (0.3, 0.49) | 0.37 (0.31, 0.45) |  | -0.001132585 | <0.001 |  | -0.001118429 | **0.003** |  | Y |
| PWY-5941: glycogen degradation II | 1.1 (0.89, 1.22) | 0.86 (0.71, 0.98) | 0.95 (0.76, 1.02) |  | -1.91E-03 | <0.001 |  | -1.88E-03 | **0.006** |  | Y |
| PWY-6317: D-galactose degradation I (Leloir pathway) | 0.66 (0.6, 0.79) | 0.56 (0.49, 0.66) | 0.6 (0.46, 0.66) |  | -0.00099737 | <0.001 |  | -0.001092018 | **0.002** |  | Y |
| PWY-6527: stachyose degradation | 0.6 (0.44, 0.68) | 0.47 (0.32, 0.55) | 0.49 (0.35, 0.57) |  | -0.001139895 | <0.001 |  | -0.000872667 | **0.01** |  | Y |
| METH-ACETATE-PWY: methanogenesis from acetate | 0.15 (0.07, 0.19) | 0.05 (0.04, 0.09) | 0.08 (0.06, 0.12) |  | -0.000733815 | <0.001 |  | -4.82E-04 | **0.039** |  | N/A |
| ASPASN-PWY: superpathway of L-aspartate and L-asparagine biosynthesis | 0.44 (0.36, 0.52) | 0.51 (0.41, 0.63) | 0.48 (0.43, 0.61) |  | 7.80E-04 | 0.004 |  | 0.001078186 | **0.011** |  | N |
| PWY-I9: L-cysteine biosynthesis VI (from L-methionine) | 0.19 (0.16, 0.28) | 0.13 (0.09, 0.21) | 0.12 (0.1, 0.17) |  | -5.72E-04 | 0.005 |  | -8.50E-04 | **0.016** |  | N |
| PWY-7663: gondoate biosynthesis (anaerobic) | 0.49 (0.34, 0.61) | 0.61 (0.48, 0.71) | 0.62 (0.51, 0.75) |  | 0.00109032 | 0.006 |  | 0.001844577 | **0.009** |  | N |
| PWY-5973: cis-vaccenate biosynthesis | 0.55 (0.39, 0.67) | 0.67 (0.54, 0.77) | 0.68 (0.58, 0.82) |  | 0.001148704 | 0.007 |  | 0.001961092 | **0.013** |  | N |
| PWY-7220: adenosine deoxyribonucleotides de novo biosynthesis II | 0.23 (0.18, 0.34) | 0.3 (0.24, 0.46) | 0.32 (0.24, 0.46) |  | 7.40E-04 | 0.009 |  | 0.001095126 | **0.013** |  | N |
| PWY-7222: guanosine deoxyribonucleotides de novo biosynthesis II | 0.23 (0.18, 0.34) | 0.3 (0.24, 0.46) | 0.32 (0.24, 0.46) |  | 0.00074023 | 0.009 |  | 0.001095126 | **0.013** |  | N |
| PWY-7228: superpathway of guanosine nucleotides de novo biosynthesis I | 0.39 (0.31, 0.5) | 0.47 (0.38, 0.64) | 0.51 (0.4, 0.64) |  | 0.000907699 | 0.011 |  | 0.001316859 | **0.015** |  | N |
| PWY-6126: superpathway of adenosine nucleotides de novo biosynthesis II | 0.44 (0.36, 0.56) | 0.55 (0.44, 0.7) | 0.58 (0.45, 0.72) |  | 0.000986275 | 0.012 |  | 0.001395603 | **0.017** |  | N |
| PWY-6125: superpathway of guanosine nucleotides de novo biosynthesis II | 0.34 (0.26, 0.45) | 0.41 (0.33, 0.58) | 0.44 (0.35, 0.58) |  | 0.000867912 | 0.013 |  | 0.001262111 | **0.013** |  | N |
| PWY-7229: superpathway of adenosine nucleotides de novo biosynthesis I | 0.58 (0.49, 0.7) | 0.71 (0.58, 0.83) | 0.74 (0.58, 0.87) |  | 1.04E-03 | 0.014 |  | 0.001357227 | **0.023** |  | N |
| PWY-4041: &gamma;-glutamyl cycle | 0.15 (0.09, 0.2) | 0.12 (0.07, 0.17) | 0.12 (0.11, 0.15) |  | -0.00028305 | 0.021 |  | -0.000368296 | **0.061** |  | N |
| PWY-841: superpathway of purine nucleotides de novo biosynthesis I | 0.5 (0.4, 0.59) | 0.57 (0.49, 0.69) | 0.62 (0.48, 0.7) |  | 7.27E-04 | 0.024 |  | 0.000976272 | **0.02** |  | N |
| PWY-7392: taxadiene biosynthesis (engineered) | 0.17 (0.08, 0.23) | 0.17 (0.11, 0.3) | 0.26 (0.12, 0.34) |  | 0.000322171 | 0.025 |  | 0.000675475 | **0.003** |  | N |
| PWY-6628: superpathway of L-phenylalanine biosynthesis | 0.43 (0.37, 0.55) | 0.38 (0.3, 0.5) | 0.38 (0.29, 0.45) |  | -0.000793417 | 0.03 |  | -0.000955731 | **0.024** |  | N |
| PWY-241: C4 photosynthetic carbon assimilation cycle, NADP-ME type | 0.11 (0.07, 0.2) | 0.08 (0.03, 0.17) | 0.06 (0.05, 0.14) |  | -0.000346444 | 0.041 |  | -0.000684718 | **0.027** |  | N |
| DAPLYSINESYN-PWY: L-lysine biosynthesis I | 0.19 (0.1, 0.24) | 0.12 (0.07, 0.23) | 0.12 (0.08, 0.2) |  | -4.38E-04 | 0.047 |  | -0.000617996 | **0.063** |  | N |
| PWY-7117: C4 photosynthetic carbon assimilation cycle, PEPCK type | 0.12 (0.08, 0.22) | 0.1 (0.04, 0.18) | 0.07 (0.06, 0.16) |  | -0.000338423 | 0.059 |  | -0.000698495 | **0.022** |  | N |
| PWY-5154: L-arginine biosynthesis III (via N-acetyl-L-citrulline) | 0.43 (0.3, 0.58) | 0.52 (0.43, 0.59) | 0.52 (0.46, 0.6) |  | 0.000538323 | 0.066 |  | 0.001378251 | **0.024** |  | N |
| PWY0-1061: superpathway of L-alanine biosynthesis | 0.11 (0.04, 0.21) | 0.07 (0.03, 0.12) | 0.06 (0.03, 0.09) |  | -0.000324238 | 0.085 |  | -0.000392323 | **0.039** |  | N |
| PWY-7208: superpathway of pyrimidine nucleobases salvage | 0.31 (0.26, 0.4) | 0.38 (0.29, 0.45) | 0.38 (0.31, 0.46) |  | 0.000447353 | 0.085 |  | 0.000619414 | **0.056** |  | N |
| CALVIN-PWY: Calvin-Benson-Bassham cycle | 0.89 (0.8, 0.96) | 0.81 (0.7, 0.9) | 0.79 (0.73, 0.89) |  | -0.000768664 | 0.005 |  | -0.000533983 | 0.164 |  | Y |
| PWY0-1296: purine ribonucleosides degradation | 0.81 (0.7, 0.93) | 0.72 (0.57, 0.83) | 0.73 (0.66, 0.84) |  | -9.10E-04 | 0.007 |  | -9.20E-04 | 0.141 |  | Y |
| PWY-6470: peptidoglycan biosynthesis V (&beta;-lactam resistance) | 0.15 (0.11, 0.23) | 0.11 (0.06, 0.15) | 0.13 (0.1, 0.18) |  | -0.000431789 | 0.009 |  | -0.000241415 | 0.322 |  | Y |
| PWY-7237: myo-, chiro- and scyllo-inositol degradation | 0.61 (0.51, 0.69) | 0.52 (0.4, 0.59) | 0.52 (0.36, 0.67) |  | -0.000815699 | 0.011 |  | -0.000557647 | 0.3 |  | Y |
| PWY-6305: superpathway of putrescine biosynthesis | 0.11 (0.08, 0.15) | 0.15 (0.1, 0.18) | 0.13 (0.08, 0.17) |  | 0.000328152 | 0.015 |  | 3.44E-05 | 0.864 |  | Y |
| ANAGLYCOLYSIS-PWY: glycolysis III (from glucose) | 0.96 (0.87, 1.02) | 0.9 (0.81, 0.94) | 0.88 (0.82, 0.98) |  | -0.000610934 | 0.016 |  | -3.93E-04 | 0.333 |  | Y |
| PWY-7357: thiamine phosphate formation from pyrithiamine and oxythiamine (yeast) | 0.71 (0.65, 0.77) | 0.61 (0.55, 0.71) | 0.64 (0.58, 0.74) |  | -0.000787108 | 0.025 |  | -0.000558309 | 0.239 |  | Y |
| PWY-6168: flavin biosynthesis III (fungi) | 0.12 (0.06, 0.19) | 0.16 (0.08, 0.28) | 0.15 (0.06, 0.21) |  | 0.00042531 | 0.035 |  | 0.000220638 | 0.259 |  | Y |
| PWY-7560: methylerythritol phosphate pathway II | 0.67 (0.58, 0.77) | 0.58 (0.47, 0.68) | 0.6 (0.49, 0.66) |  | -0.000733665 | 0.049 |  | -0.000550985 | 0.131 |  | Y |
| **DTDPRHAMSYN-PWY: dTDP-&beta;-L-rhamnose biosynthesis** | 1.13 (0.99, 1.29) | 1.23 (1.06, 1.34) | 1.17 (1.11, 1.31) |  | 0.000984377 | 0.059 |  | 0.000989792 | 0.148 |  | Y |
| PWY0-1477: ethanolamine utilization | 0.1 (0.06, 0.16) | 0.13 (0.09, 0.2) | 0.11 (0.07, 0.16) |  | 0.000346288 | 0.084 |  | -0.000145389 | 0.556 |  | Y |
| SER-GLYSYN-PWY: superpathway of L-serine and glycine biosynthesis I | 0.61 (0.56, 0.65) | 0.57 (0.49, 0.65) | 0.59 (0.51, 0.69) |  | -3.64E-04 | 0.09 |  | 0.000117954 | 0.81 |  | Y |
| PWY-1861: formaldehyde assimilation II (assimilatory RuMP Cycle) | 0.13 (0.04, 0.2) | 0.05 (0.02, 0.09) | 0.06 (0.03, 0.13) |  | -0.000578964 | <0.001 |  | -0.000485232 | 0.141 |  | Y |
| PWY-6270: isoprene biosynthesis I | 0.67 (0.56, 0.73) | 0.56 (0.44, 0.63) | 0.62 (0.52, 0.66) |  | -9.37E-04 | <0.001 |  | -0.000458372 | 0.172 |  | Y |
| PWY-3001: superpathway of L-isoleucine biosynthesis I | 0.71 (0.64, 0.74) | 0.64 (0.58, 0.69) | 0.67 (0.63, 0.7) |  | -5.80E-04 | 0.004 |  | -2.60E-04 | 0.193 |  | N |
| COBALSYN-PWY: superpathway of adenosylcobalamin salvage from cobinamide I | 0.44 (0.34, 0.52) | 0.33 (0.25, 0.44) | 0.35 (0.28, 0.4) |  | -0.000746529 | 0.008 |  | -0.000726257 | 0.178 |  | N |
| PWY-6507: 4-deoxy-L-threo-hex-4-enopyranuronate degradation | 0.12 (0.09, 0.16) | 0.15 (0.12, 0.2) | 0.15 (0.11, 0.18) |  | 0.000258362 | 0.02 |  | 8.06E-05 | 0.732 |  | N |
| THISYNARA-PWY: superpathway of thiamine diphosphate biosynthesis III (eukaryotes) | 0.46 (0.4, 0.52) | 0.39 (0.34, 0.48) | 0.4 (0.36, 0.47) |  | -0.000447834 | 0.041 |  | -0.000305904 | 0.3 |  | N |
| GALACTUROCAT-PWY: D-galacturonate degradation I | 0.14 (0.1, 0.18) | 0.16 (0.13, 0.22) | 0.17 (0.12, 0.19) |  | 2.31E-04 | 0.042 |  | 4.07E-05 | 0.858 |  | N |
| PWY-6703: preQ0 biosynthesis | 0.56 (0.39, 0.65) | 0.6 (0.5, 0.71) | 0.58 (0.49, 0.67) |  | 0.000750592 | 0.048 |  | 0.000813046 | 0.239 |  | N |
| OANTIGEN-PWY: O-antigen building blocks biosynthesis (E. coli) | 0.6 (0.45, 0.67) | 0.53 (0.42, 0.61) | 0.54 (0.42, 0.61) |  | -0.000584423 | 0.05 |  | -0.00070965 | 0.22 |  | N |
| GLUCUROCAT-PWY: superpathway of &beta;-D-glucuronosides degradation | 0.17 (0.12, 0.21) | 0.2 (0.17, 0.26) | 0.2 (0.16, 0.23) |  | 0.000286695 | 0.05 |  | 4.77E-05 | 0.81 |  | N |
| UDPNAGSYN-PWY: UDP-N-acetyl-D-glucosamine biosynthesis I | 0.46 (0.31, 0.53) | 0.36 (0.3, 0.46) | 0.38 (0.32, 0.47) |  | -5.87E-04 | 0.053 |  | -8.64E-04 | 0.128 |  | N |
| PWY-6608: guanosine nucleotides degradation III | 0.21 (0.18, 0.27) | 0.19 (0.11, 0.23) | 0.17 (0.12, 0.22) |  | -0.000441302 | 0.061 |  | -0.000357777 | 0.138 |  | N |
| PWY-724: superpathway of L-lysine, L-threonine and L-methionine biosynthesis II | 0.8 (0.75, 0.84) | 0.77 (0.69, 0.82) | 0.79 (0.72, 0.84) |  | -0.000337095 | 0.066 |  | 3.63E-05 | 0.864 |  | N |
| PWY-621: sucrose degradation III (sucrose invertase) | 0.25 (0.16, 0.31) | 0.23 (0.16, 0.29) | 0.26 (0.18, 0.34) |  | -0.000253351 | 0.066 |  | -0.000276955 | 0.322 |  | N |
| RHAMCAT-PWY: L-rhamnose degradation I | 0.34 (0.28, 0.43) | 0.38 (0.3, 0.44) | 0.4 (0.31, 0.46) |  | 0.000337352 | 0.078 |  | 0.000452573 | 0.217 |  | N |
| PWY-7323: superpathway of GDP-mannose-derived O-antigen building blocks biosynthesis | 0.2 (0.16, 0.25) | 0.22 (0.18, 0.3) | 0.21 (0.14, 0.28) |  | 0.000171985 | 0.084 |  | -4.33E-06 | 0.976 |  | N |
| ARO-PWY: chorismate biosynthesis I | 1.07 (1.03, 1.12) | 1.08 (0.95, 1.11) | 1.05 (0.98, 1.13) |  | -0.000558025 | 0.098 |  | -0.000292359 | 0.781 |  | N |
